# Supplementary material for: Safe eradication of large established tumors using neovasculature‐targeted tumor necrosis factor‐based therapies
Source: EMBO Mol Med. 2020 Jan 8;12(2):e11223. doi: 10.15252/emmm.201911223 (PMC7709889; doi:10.15252/emmm.201911223)
Supplement: Supplementary file 1 — Appendix [file EMMM-12-e11223-s001.pdf]

Safe eradication of large established tumors using neovasculature-targeted Tumor Necrosis Factor-based therapies

Leander Huyghe, Alexander Van Parys, Anje Cauwels, Sandra Van Lint, Stijn De Munter, Jennyfer Bultinck, Lennart Zabeau, Jeroen Hostens, An Goethals, Nele Vanderroost, Annick Verhee, Gilles Uzé, Niko Kley, Frank Peelman, Bart Vandekerckhove, Peter Brouckaert & Jan Tavernier

Appendix

Appendix Table S1

Table S1. Multiplicity adjusted p-values

Figure	Comparison	Statistical test	Adjusted p-value
2D, 20 ng/ml	ctrl vs. Y87F	One-way ANOVA + Bonferroni multiple comparison	>0.9999
	ctrl vs. AFR		0.0006
	AFR vs. Y87F		0.0016
	AFR vs. WT		0.0004
2D, 0.16 ng/ml	ctrl vs. Y87F	One-way ANOVA + Bonferroni multiple comparison	>0.9999
	ctrl vs. AFR		0.0043
	AFR vs. Y87F		0.0056
	AFR vs. WT		<0.0001
3B	Ctrl vs. sc mTNF WT 35µg	One-way ANOVA + Bonferroni multiple comparison	0.0034
	Ctrl vs. sc mTNF Y86F 200µg		>0.9999
3D, ICAM-1	PBS vs. mTNF	One-way ANOVA + Bonferroni multiple comparison	0.0018
	PBS vs. AFR		0.0056
	mTNF vs. AFR		>0.9999
3D, E-selectin	PBS vs. mTNF	One-way ANOVA + Bonferroni multiple comparison	<0.0001
	PBS vs. AFR		0.0436
	mTNF vs. AFR		0.0016
3D, VEGF-R2	PBS vs. mTNF	One-way ANOVA + Bonferroni multiple comparison	<0.0001
	PBS vs. AFR		<0.0001
	mTNF vs. AFR		>0.9999
3E, TSI	PBS vs. CD13-AFR	Two-way ANOVA + Bonferroni multiple comparison	<0.0001
3G	PBS vs. Wortmannin	One-way ANOVA + Bonferroni multiple comparison	0.1652
	PBS vs. Birinapant		>0.9999
	PBS vs. CD13-AFR		0.0438
	PBS vs. Wm + CD13-AFR		<0.0001
	PBS vs. Bir + CD13-AFR		<0.0001
	CD13-AFR vs. Wm + CD13-AFR		0.0005
	CD13-AFR vs. Bir + CD13-AFR		0.0001
	Wortmannin vs. Wm + CD13-AFR		0.0004
	Birinapant vs. Bir + CD13-AFR		<0.0001
4B	CAR T: PBS vs. CAR T: AFR	Two-way ANOVA + Bonferroni multiple comparison	<0.0001
	PBS vs. CD13-AFR		0.1300
	PBS vs. CAR T: PBS		>0.9999
4F	PBS vs. CD8-AFN	One-way ANOVA + Bonferroni multiple comparison	0.7084
	PBS vs. CD13-AFR		0.0157
	CD8-AFN vs. CD13-AFR + CD8-AFN		<0,0001
	CD13-AFR vs. CD13-AFR + CD8-AFN		0.0002
5A	hTNF vs. hTNF + IFN-γ, day 19	One-way ANOVA + Bonferroni multiple comparison	0.0019
5B	hTNF vs. hTNF + IFN-γ, day 19	One-way ANOVA + Bonferroni multiple comparison	0.2684
5C, 20 ng/ml	Y87F - vs. +	One-way ANOVA + Bonferroni multiple comparison	>0.9999
	AFR - vs. +		0.0006
	WT - vs. +		<0.0001
	AFR + vs. WT -		>0.9999

5C, 0.8 ng/ml	Y87F - vs. +<br>AFR - vs. +<br>WT - vs. +<br>AFR + vs. WT -	One-way ANOVA + Bonferroni multiple comparison	>0.9999<br>0.0042<br><0.0001<br>>0.9999
EV3, TNF-R1	Ctrl vs. IFN-γ 2h<br>Ctrl vs. IFN-γ 6h<br>Ctrl vs. IFN-γ 24h	One-way ANOVA + Bonferroni multiple comparison	0.0813<br>0.0123<br>0.0628
