# Supplementary material for: Safe eradication of large established tumors using neovasculature‐targeted tumor necrosis factor‐based therapies
Source: EMBO Mol Med. 2020 Jan 8;12(2):e11223. doi: 10.15252/emmm.201911223 (PMC7709889; doi:10.15252/emmm.201911223)
Supplement: Supplementary file 2 — Expanded View Figures PDF [file EMMM-12-e11223-s002.pdf]

Expanded View Figures

Figure EV1. mCD13-AFR increases CAR T-cell infiltration in SKOV3 tumors, and antigen specificity of hCD70 CAR T and mCD13-AFR combination therapy.

A Gating strategy for flow cytometric analysis of PBMCs in SKOV3 tumors. First, cell debris and bulk SKOV3 cells were gated out ("non-debris"), then, PI-positive dead cells were removed ("live"), and finally, doublets were gated out ("single cells").

B, C NSG mice were i.v. injected with 2.8×10^6 CAR T cells on day 14 after tumor inoculation and treated p.l. with PBS (B) or 50 µg CD13-AFR (C) on days 14, 15, 16, 25, 26, and 28 (n = 5). Flow cytometric analysis was performed on day 43. Human PBMCs were identified as hCD45 and hCD3 double positive cells (upper panel). The presence of the CAR transgene in human PBMCs was confirmed by eGFP expression (lower panel).

D RL tumor growth in NSG mice after i.v. injection of 6×10^6 hCD70 CAR T cells on day 14 and p.l. treatment with PBS or 50 µg mCD13-AFR (↑). Tumor growth is shown as mean TSI, and error bars are SEM (n = 5).

Source data are available online for this figure.

 © 2020 The Authors

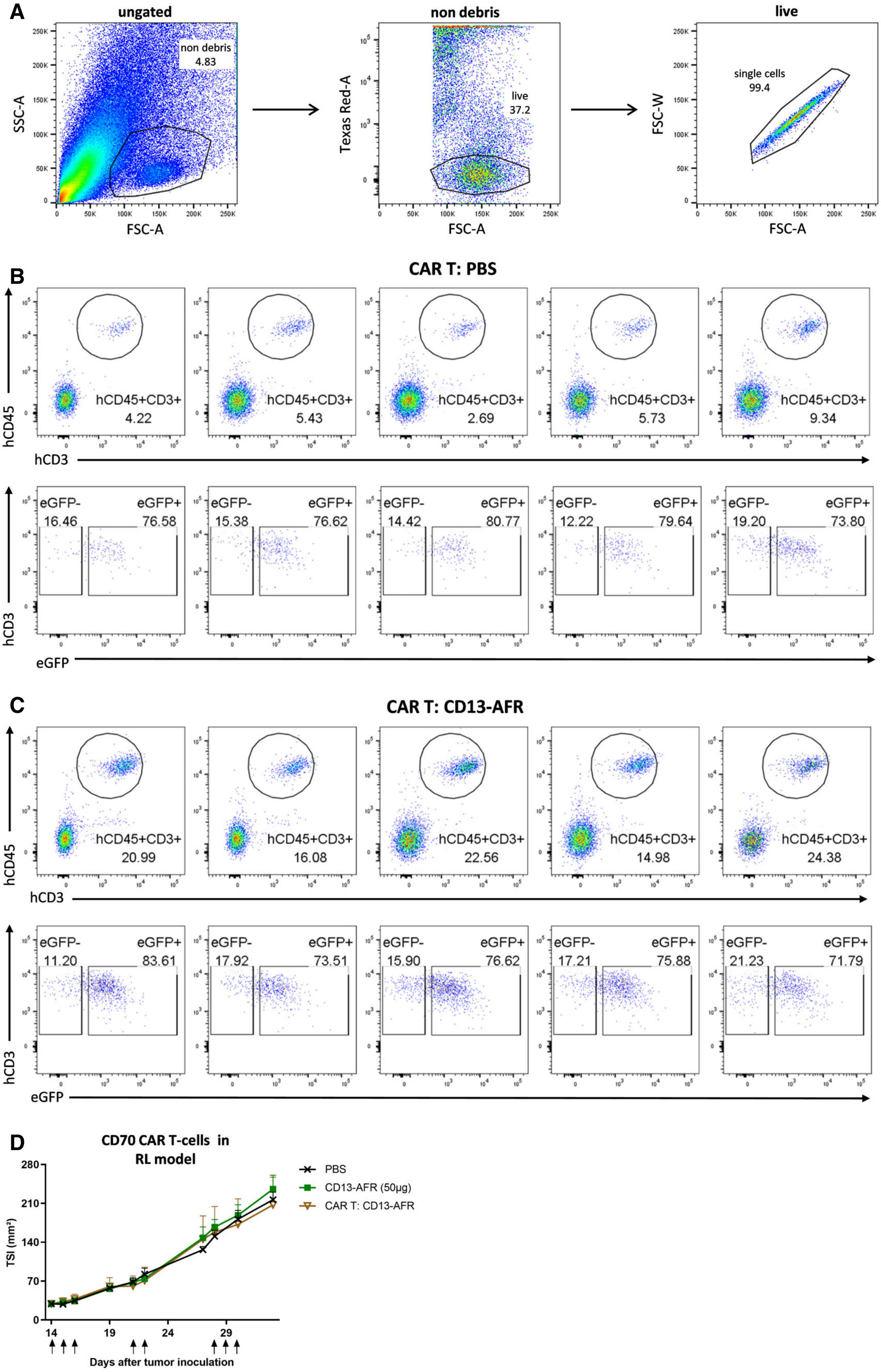

A
ungated
non debris
4.83
SSC-A
FSC-A
non debris
Texas Red-A
live
37.2
FSC-A
live
FSC-W
single cells
99.4
FSC-A
B
CAR T: PBS
hCD45+CD3+
4.22
5.43
2.69
5.73
9.34
hCD45
hCD3
eGFP-
eGFP+
16.46
76.58
15.38
76.62
14.42
80.77
12.22
79.64
19.20
73.80
hCD3
eGFP
C
CAR T: CD13-AFR
hCD45+CD3+
20.99
16.08
22.56
14.98
24.38
hCD45
hCD3
eGFP-
eGFP+
11.20
83.61
17.92
73.51
15.90
76.62
17.21
75.88
21.23
71.79
hCD3
eGFP
D
CD70 CAR T-cells in RL model
PBS
CD13-AFR (50µg)
CAR T: CD13-AFR
TSI (mm²)
Days after tumor inoculation

Figure EV1.

Figure EV2. The synergistic antitumor effect of TNF and IFN-γ depends on expression of IFN-γR on host cells, and endothelial expression of the dnIFN-γR1 transgene in Flk1 dnIFN-γR$^{tg/tg}$ mice.

A, B TSI of B16-dnIFN-γR tumor in wt C57BL/6 mice (A) or B16Bl6 parental tumor in IFN-γR$^{-/-}$ mice (B). In wt mice, the growth rate of a B16-dnIFN-γR tumor was similar to B16Bl6 parental tumor. Mice were treated daily via p.l. injection of 7 μg mTNF, 30 μg hTNF, or a combination of 30 μg hTNF and 10 000 IU mIFN-γ. Data are shown as mean TSI + SEM (n = 7). The line under the graph represents the treatment period.

C Cryosections of Flk1 dnIFN-γR$^{tg/tg}$ embryos on day E10.5 were stained with anti-c-Myc Ab, followed by chromogenic detection with alkaline phosphatase-conjugated anti-mIgG Ab and BCIP/NBT substrate. Nuclei were stained with hematoxylin. Expression was visible in endothelial cells of large veins (v), sinus venosus (sv), and aorta (ao). Scale bars are 50 μm.

D, E Cryosections of B16Bl6 tumor, liver, and spleen of adult Flk1 dnIFN-γR$^{tg/tg}$ mice were stained for c-Myc and PECAM-1 (D) or CD45 (E). Nuclei were stained with Hoechst. Endothelial cells in tumor and liver showed positive staining for the transgene, while immune cells were mostly negative. Scale bars are 50 μm.

Source data are available online for this figure.

© 2020 The Authors

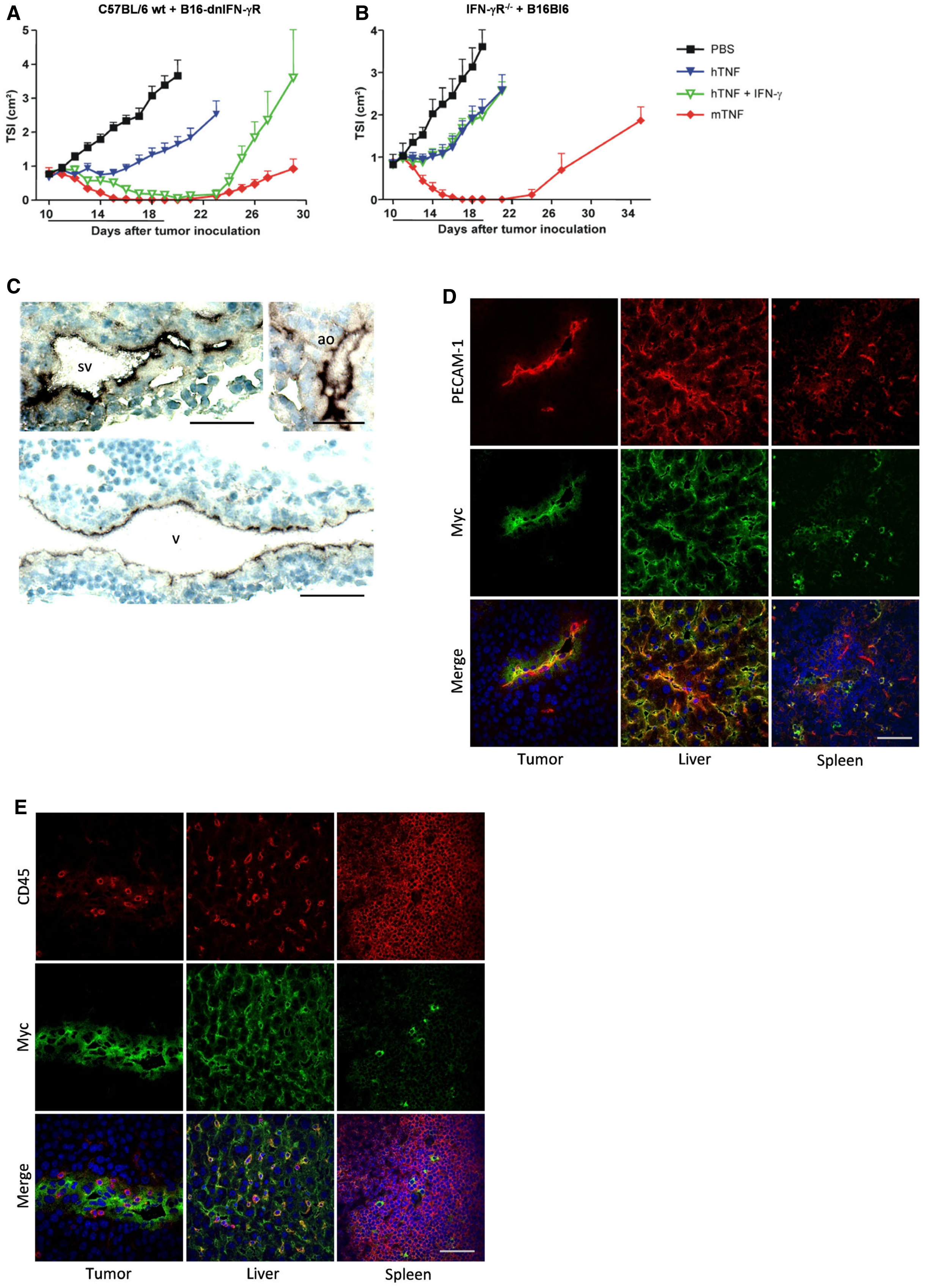
A
C57BL/6 wt + B16-dnIFN-γR
TSI (cm²)
Days after tumor inoculation
B
IFN-γR-/- + B16Bl6
TSI (cm²)
Days after tumor inoculation
PBS
hTNF
hTNF + IFN-γ
mTNF
C
sv
ao
v
D
PECAM-1
Myc
Merge
Tumor
Liver
Spleen
E
CD45
Myc
Merge
Tumor
Liver
Spleen

Figure EV2.

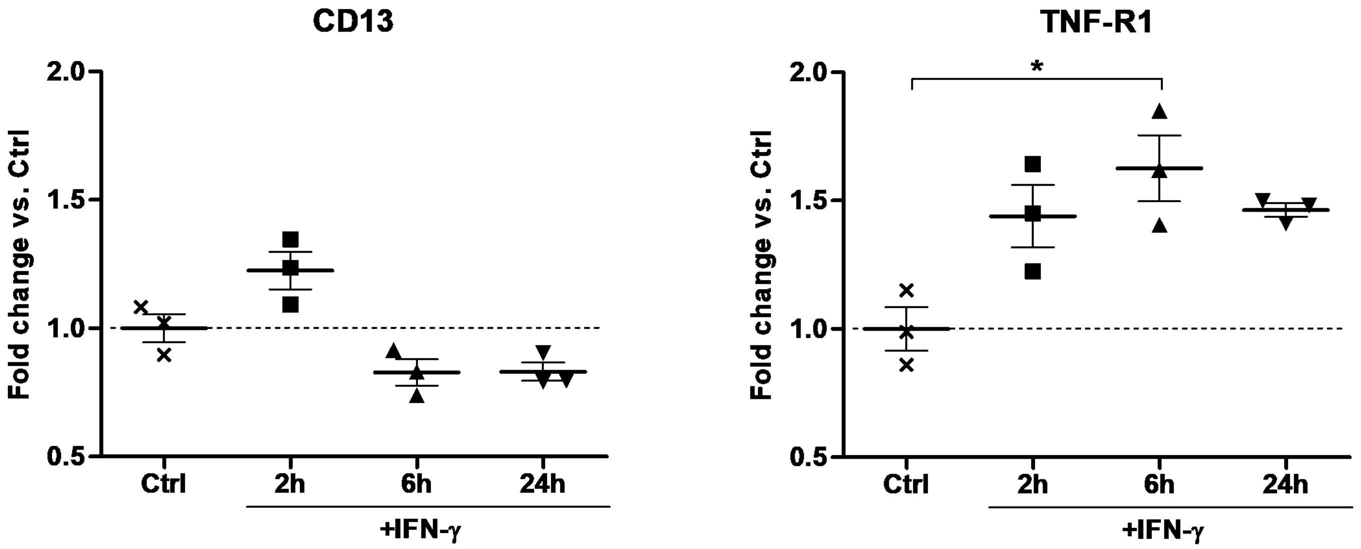

Figure EV3. IFN-γ increases TNF-R1 expression in HUVECs.

qPCR analysis for TNF-R1 and CD13 expression on RNA isolated from HUVECs after stimulation with 100 ng/ml hIFN-γ. Individual values are shown. The horizontal lines indicate the mean per condition. Error bars are SEM. *$P < 0.05$ by one-way ANOVA with Bonferroni's multiple comparison test.

Source data are available online for this figure.

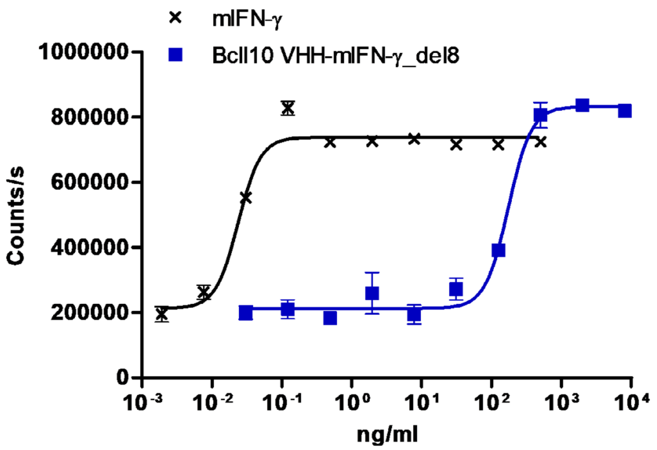

Figure EV4. Bioactivity of mIFN-γ del8 mutant in the EMCV cytopathic effect assay in L929 cells.

L929 cells were stimulated for 24 h with wt mIFN-γ or del8 mutant before virus (~ 99% CPE) was added. One day later, cell viability was measured via an ATP luminescence assay.

Source data are available online for this figure.

© 2020 The Authors

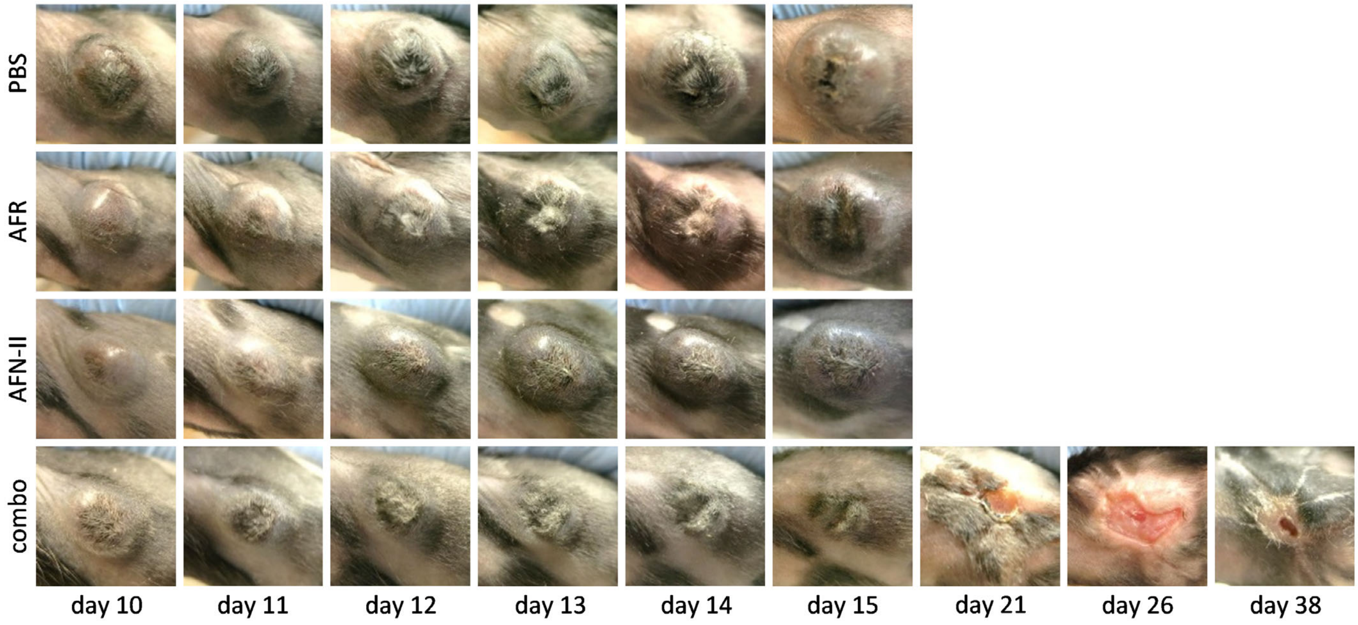

Figure EV5. CD13-AFR/CD13-AFN-II combination therapy induces tumor necrosis.

Representative images of B16Bl6 tumors treated p.l. with 50 μg mCD13-AFR and/or 23.6 μg mCD13-AFN-II, at indicated day after tumor inoculation, are shown. Mice were treated daily from day 10 to day 14.

Source data are available online for this figure.
