# Supplementary figures and images for: Safe eradication of large established tumors using neovasculature‐targeted tumor necrosis factor‐based therapies
Source: EMBO Mol Med. 2020 Jan 8;12(2):e11223. doi: 10.15252/emmm.201911223 (PMC7709889; doi:10.15252/emmm.201911223)

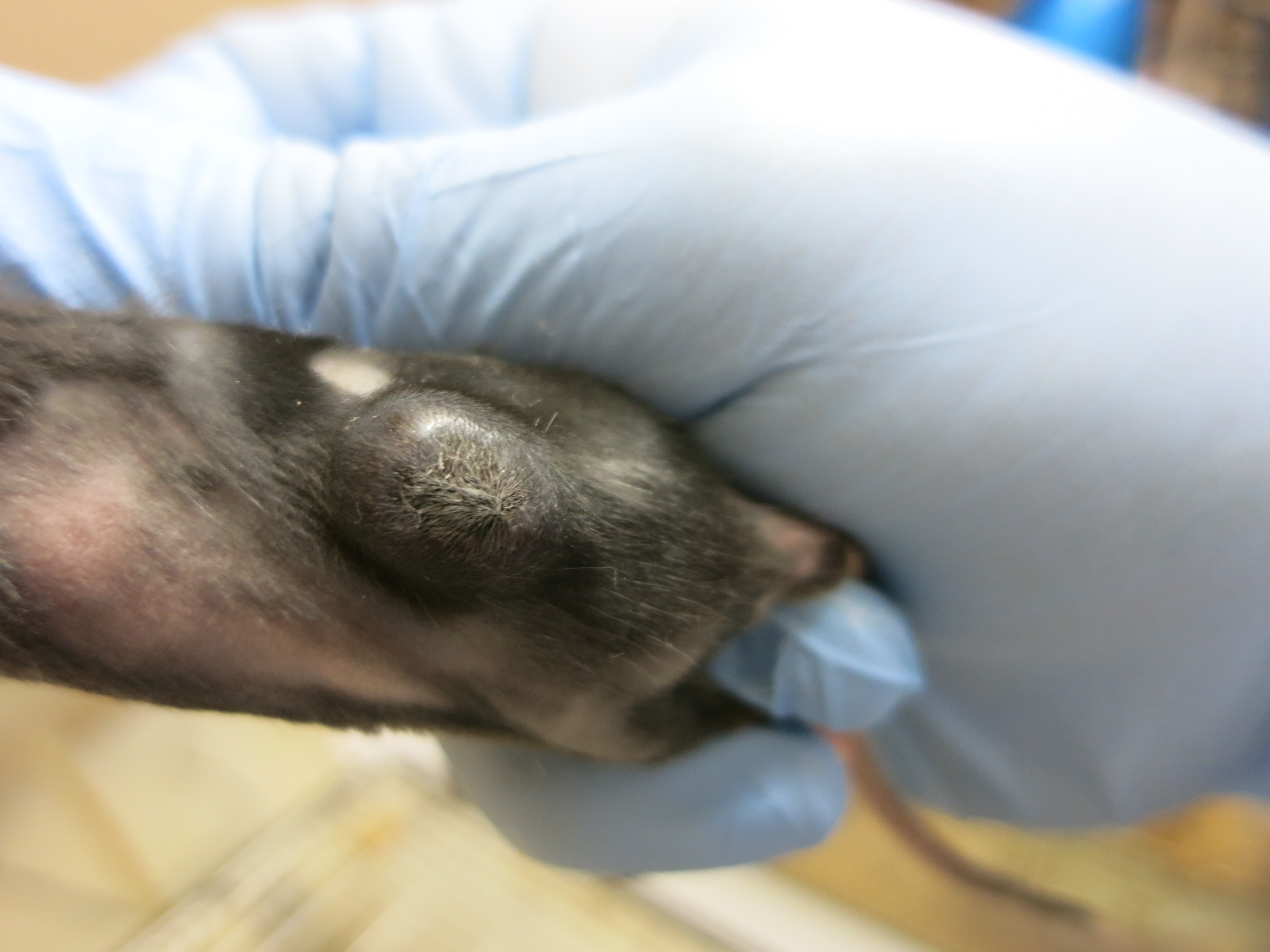

Supplement: Supplementary file 3 — Source Data for Expanded View [file EMMM-12-e11223-s009.zip › EV_source-data/Fig.EV5/AFN-II d14.JPG]

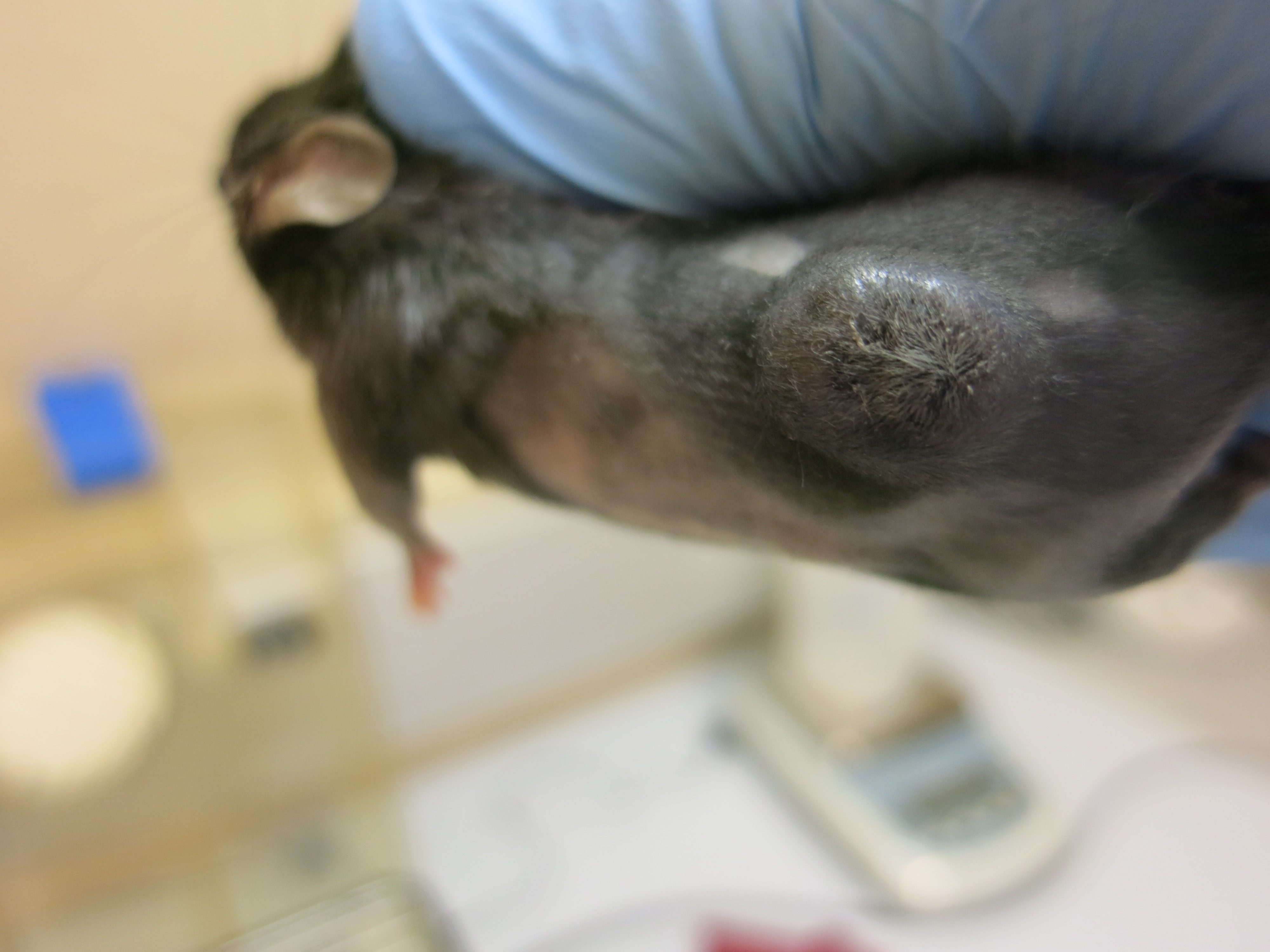

Supplement: Supplementary file 3 — Source Data for Expanded View [file EMMM-12-e11223-s009.zip › EV_source-data/Fig.EV5/AFN-II d15.JPG]

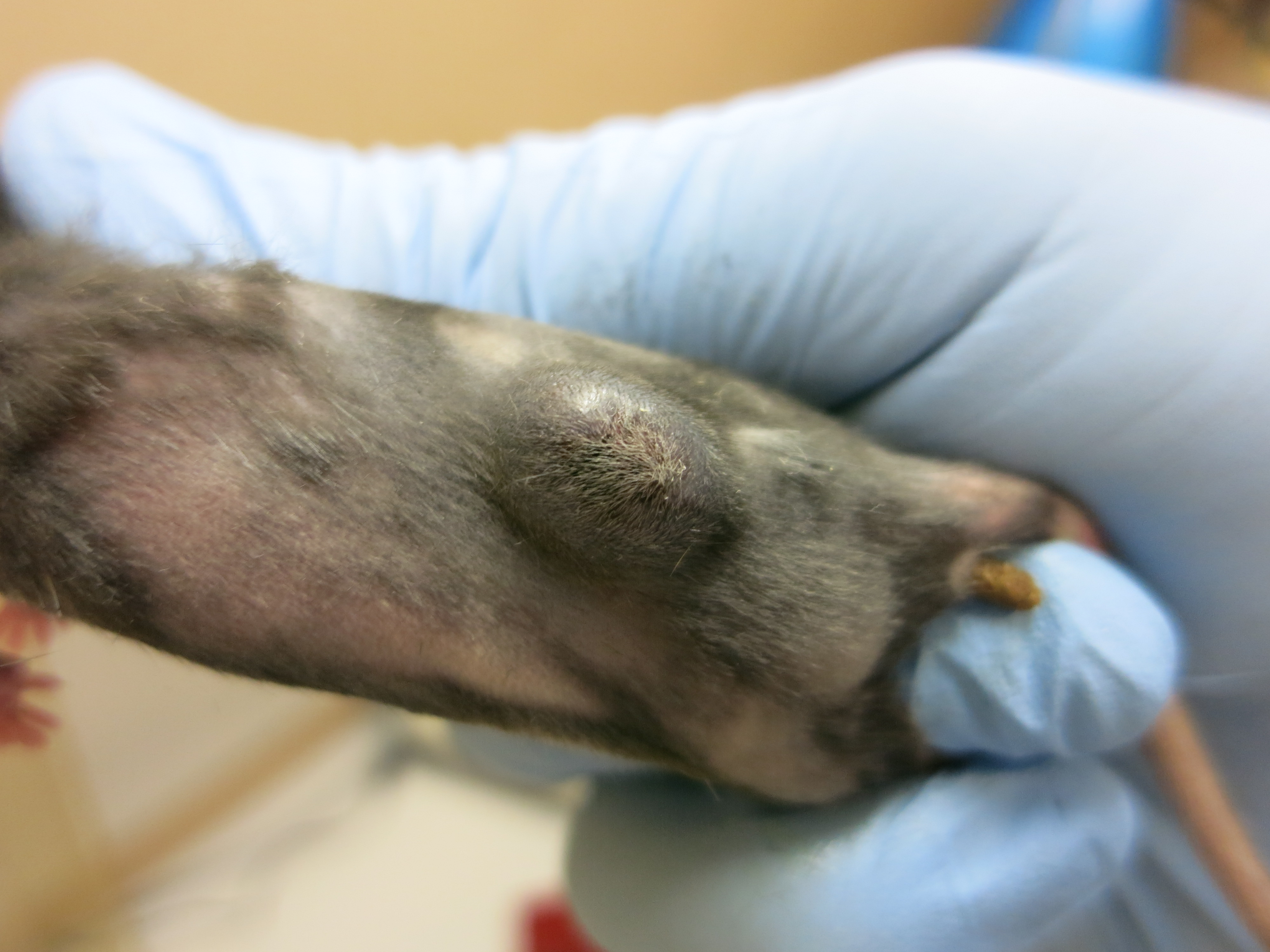

Supplement: Supplementary file 3 — Source Data for Expanded View [file EMMM-12-e11223-s009.zip › EV_source-data/Fig.EV5/AFN-II d12.JPG]

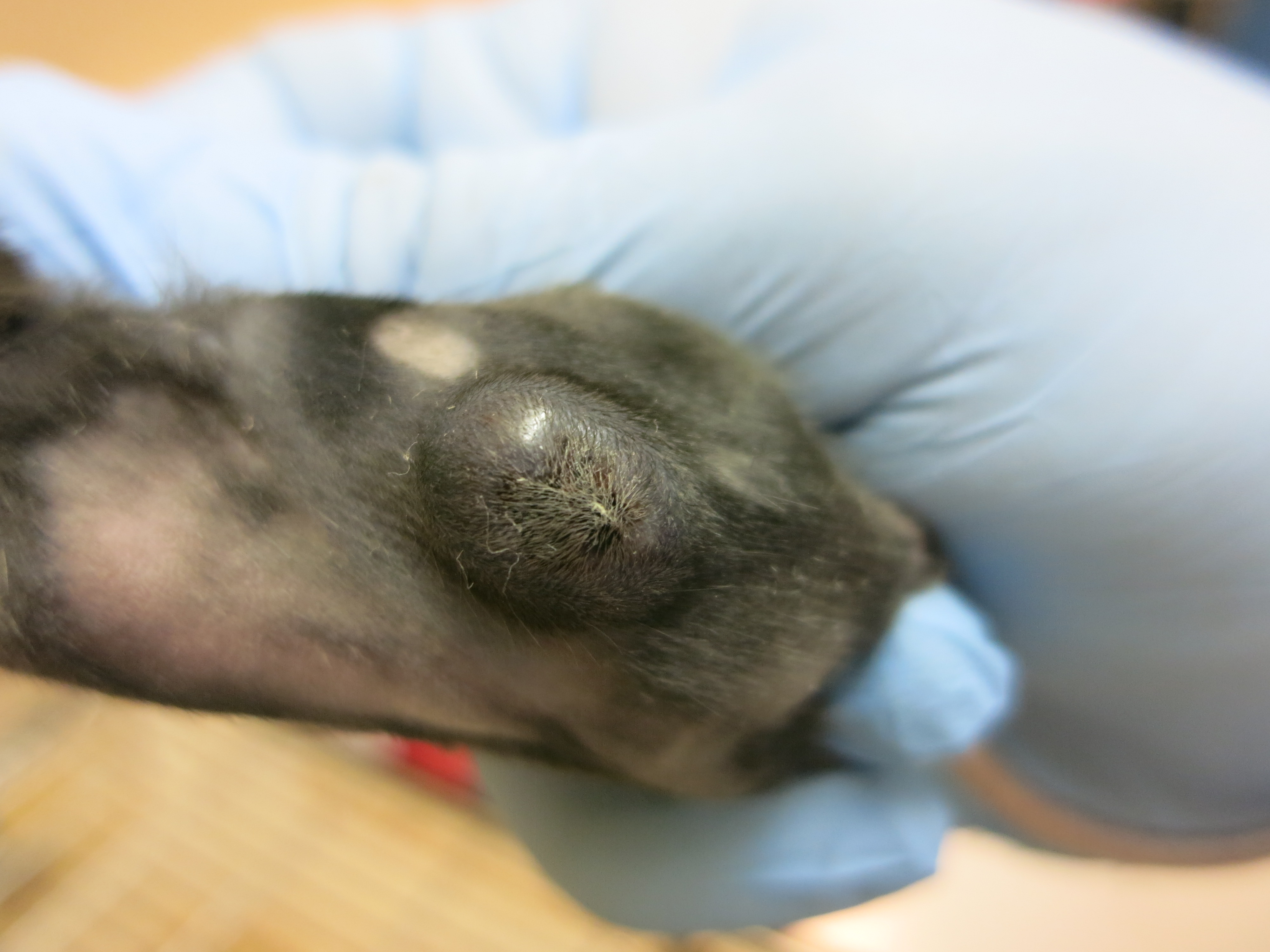

Supplement: Supplementary file 3 — Source Data for Expanded View [file EMMM-12-e11223-s009.zip › EV_source-data/Fig.EV5/AFN-II d13.JPG]

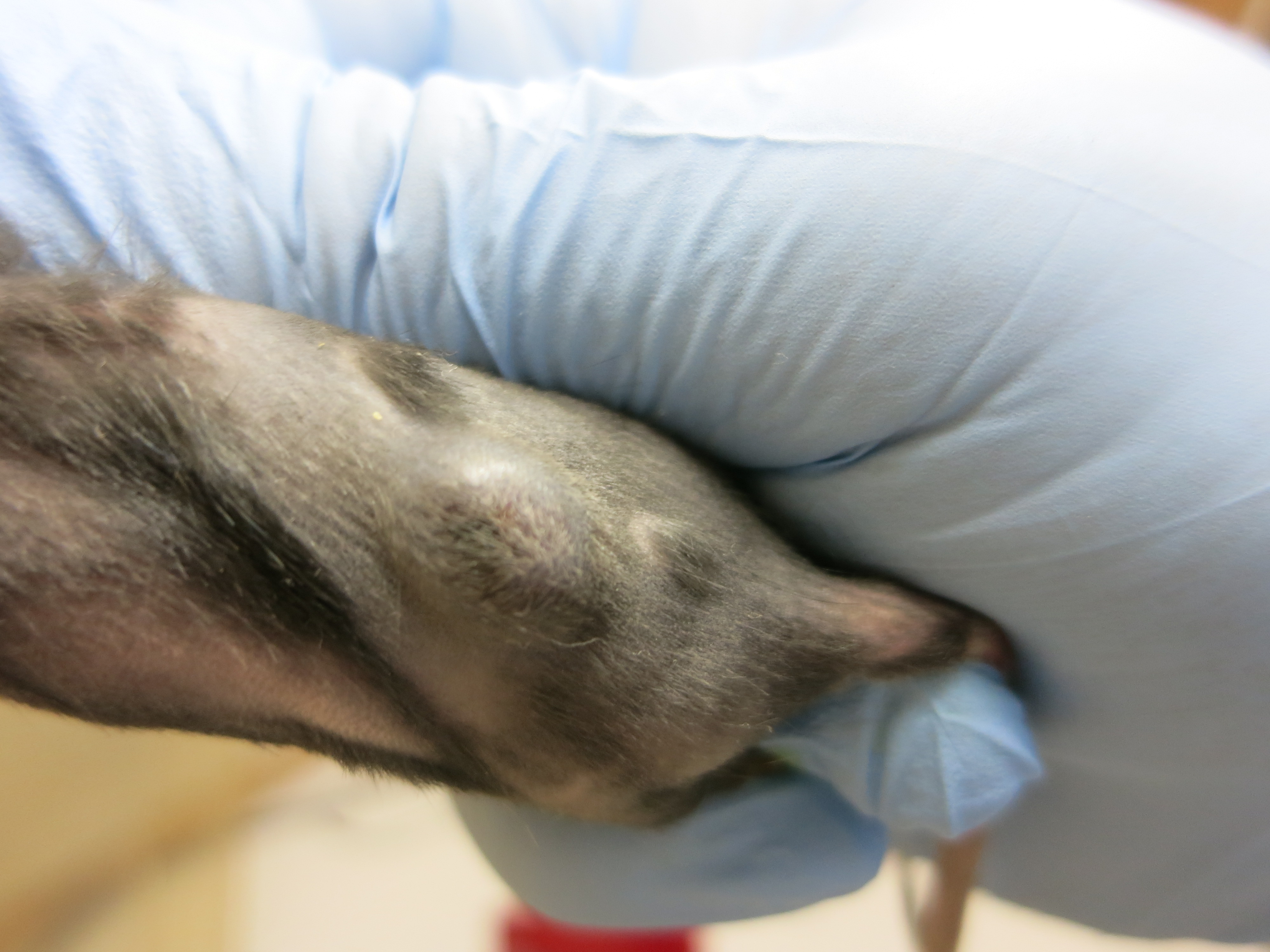

Supplement: Supplementary file 3 — Source Data for Expanded View [file EMMM-12-e11223-s009.zip › EV_source-data/Fig.EV5/AFN-II d11.JPG]

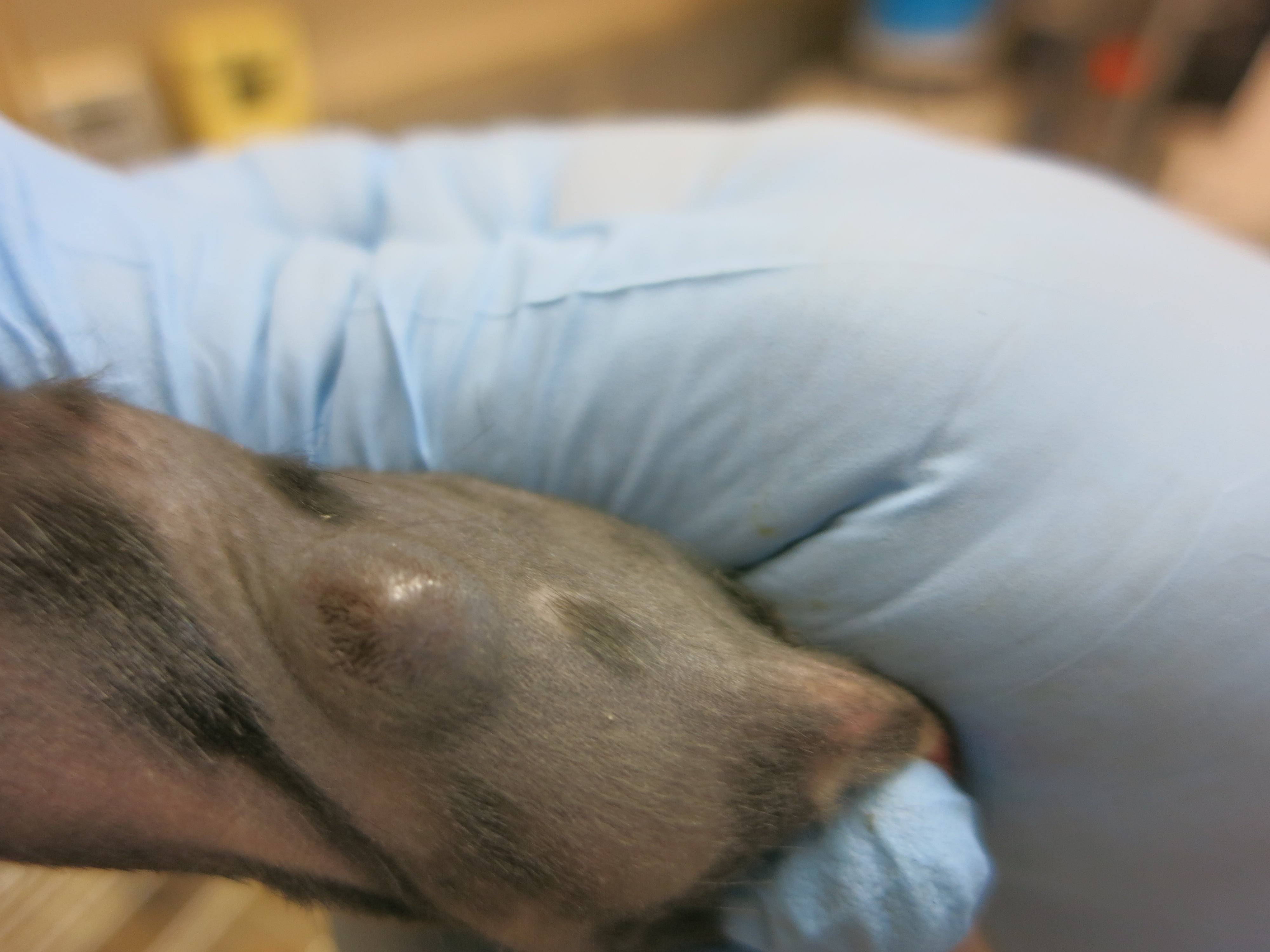

Supplement: Supplementary file 3 — Source Data for Expanded View [file EMMM-12-e11223-s009.zip › EV_source-data/Fig.EV5/AFN-II d10.JPG]

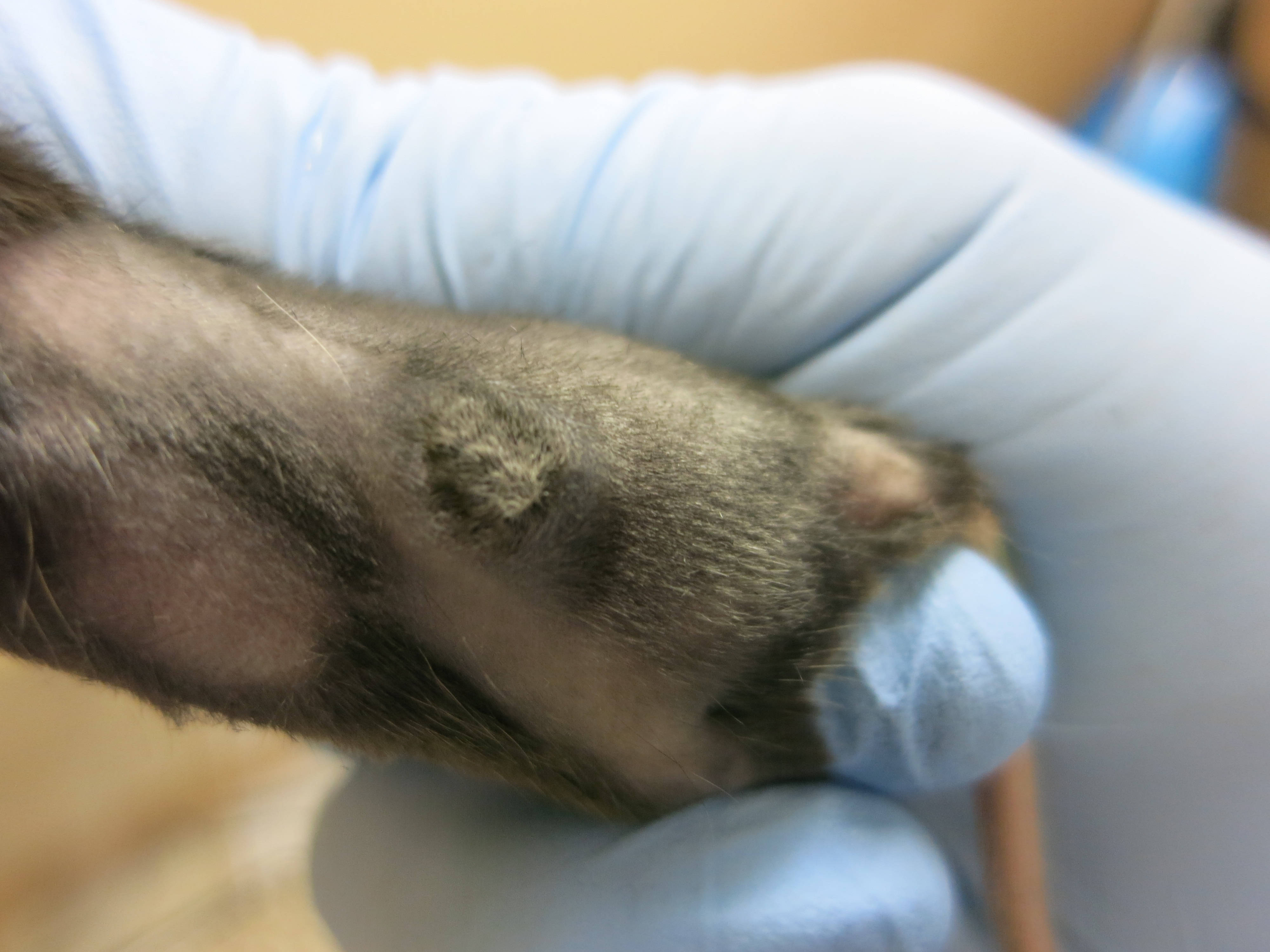

Supplement: Supplementary file 3 — Source Data for Expanded View [file EMMM-12-e11223-s009.zip › EV_source-data/Fig.EV5/combo d12.JPG]

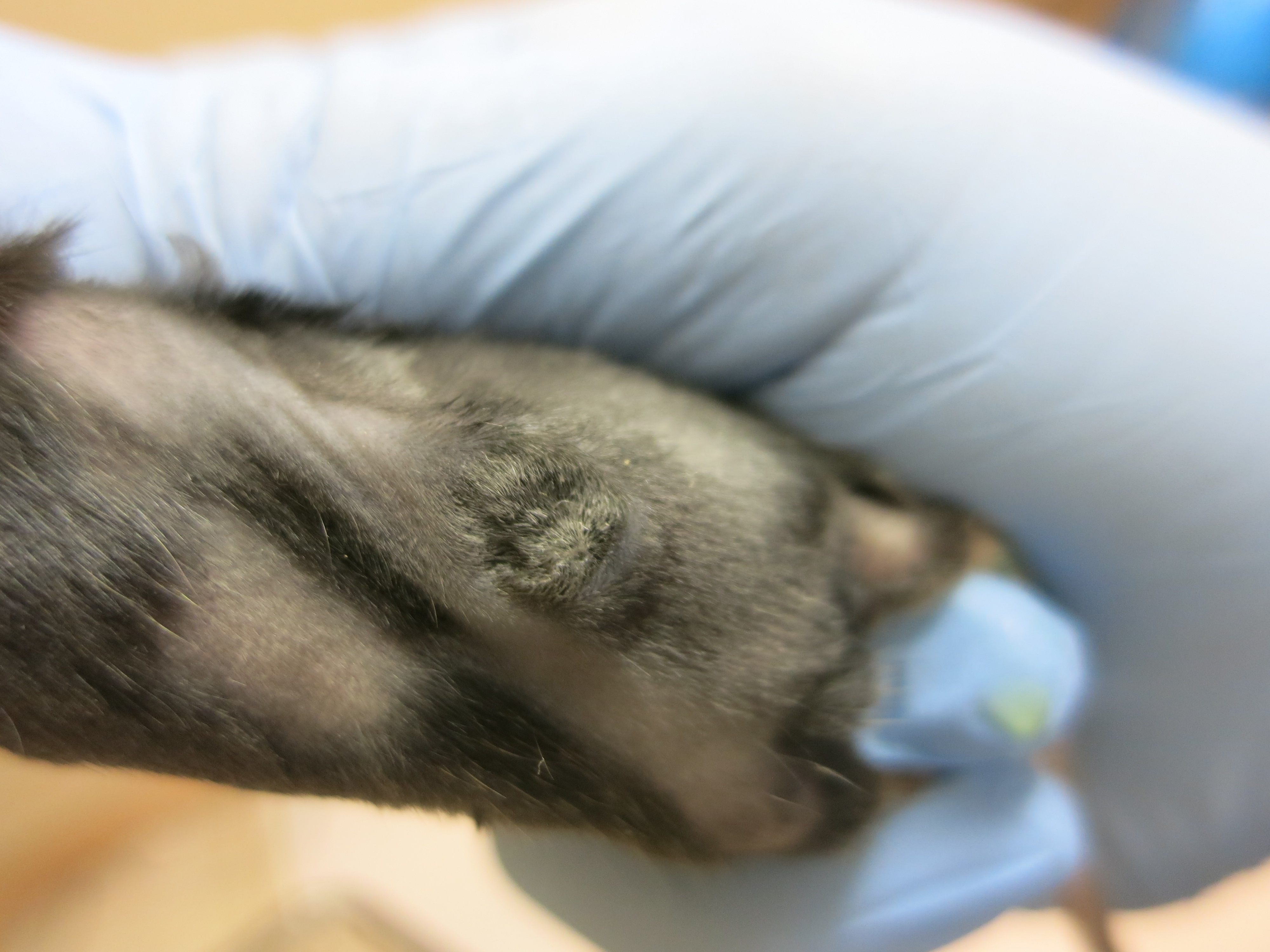

Supplement: Supplementary file 3 — Source Data for Expanded View [file EMMM-12-e11223-s009.zip › EV_source-data/Fig.EV5/combo d13.JPG]

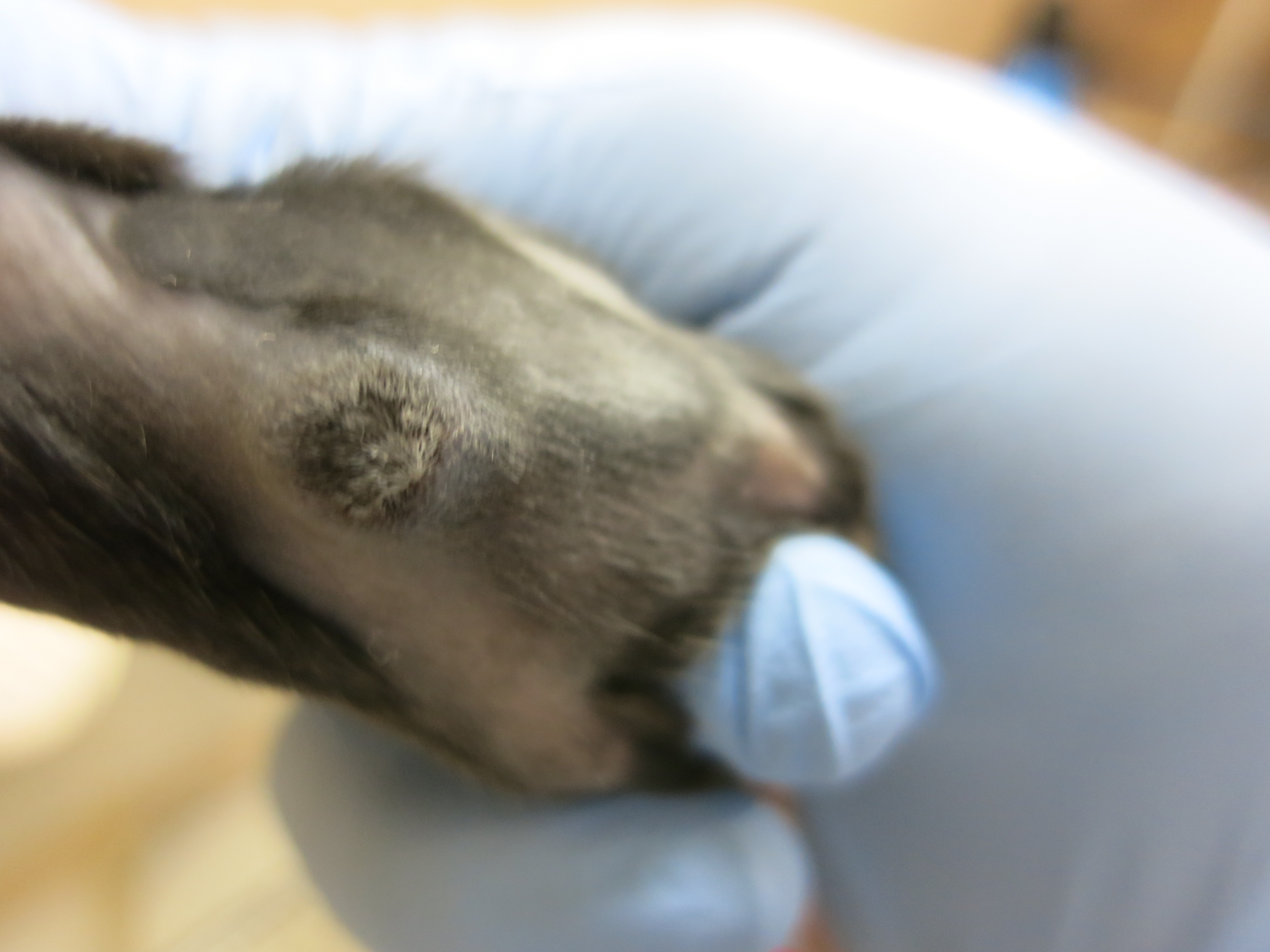

Supplement: Supplementary file 3 — Source Data for Expanded View [file EMMM-12-e11223-s009.zip › EV_source-data/Fig.EV5/combo d11.JPG]

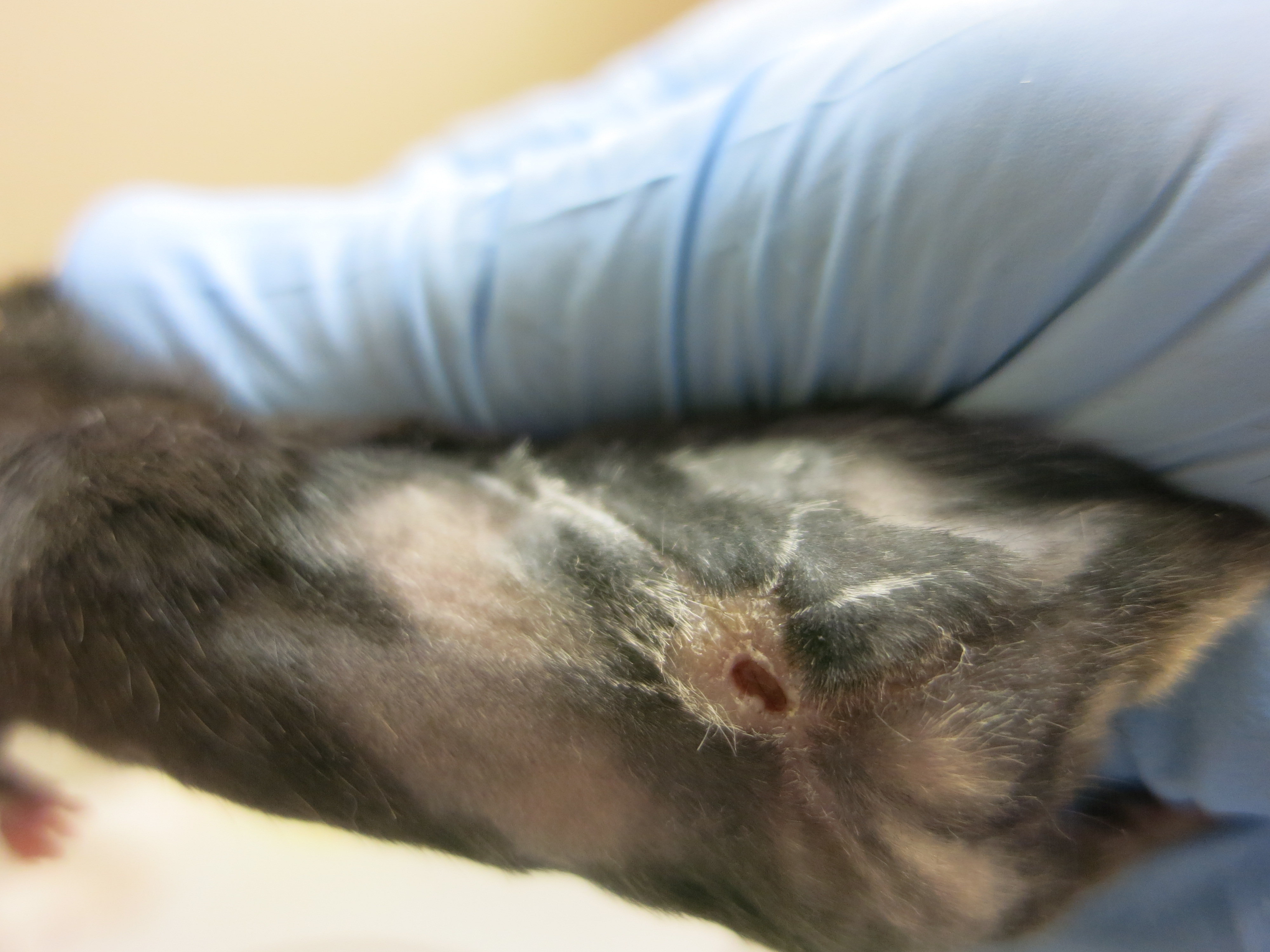

Supplement: Supplementary file 3 — Source Data for Expanded View [file EMMM-12-e11223-s009.zip › EV_source-data/Fig.EV5/combo d38.JPG]

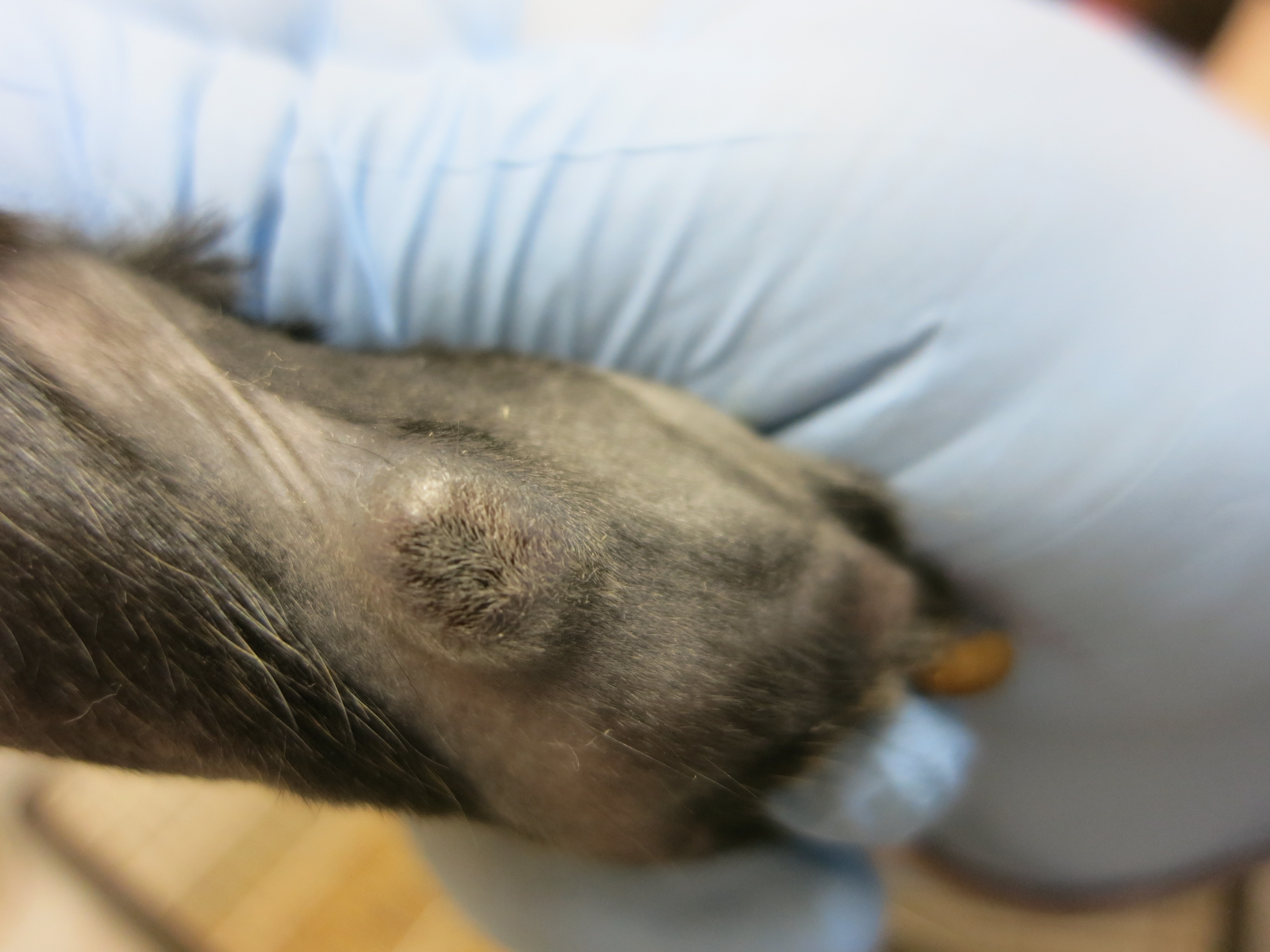

Supplement: Supplementary file 3 — Source Data for Expanded View [file EMMM-12-e11223-s009.zip › EV_source-data/Fig.EV5/combo d10.JPG]

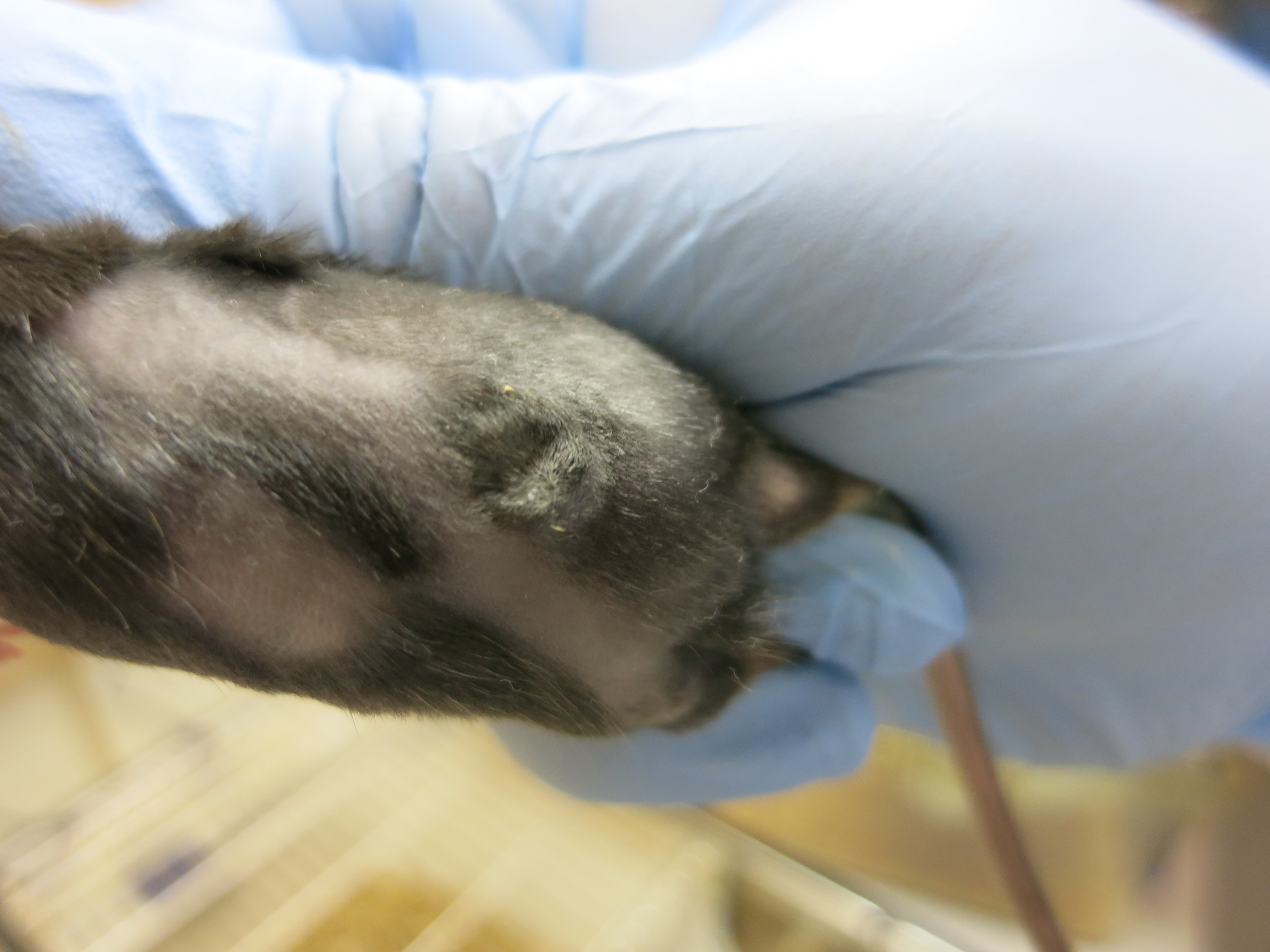

Supplement: Supplementary file 3 — Source Data for Expanded View [file EMMM-12-e11223-s009.zip › EV_source-data/Fig.EV5/combo d14.JPG]

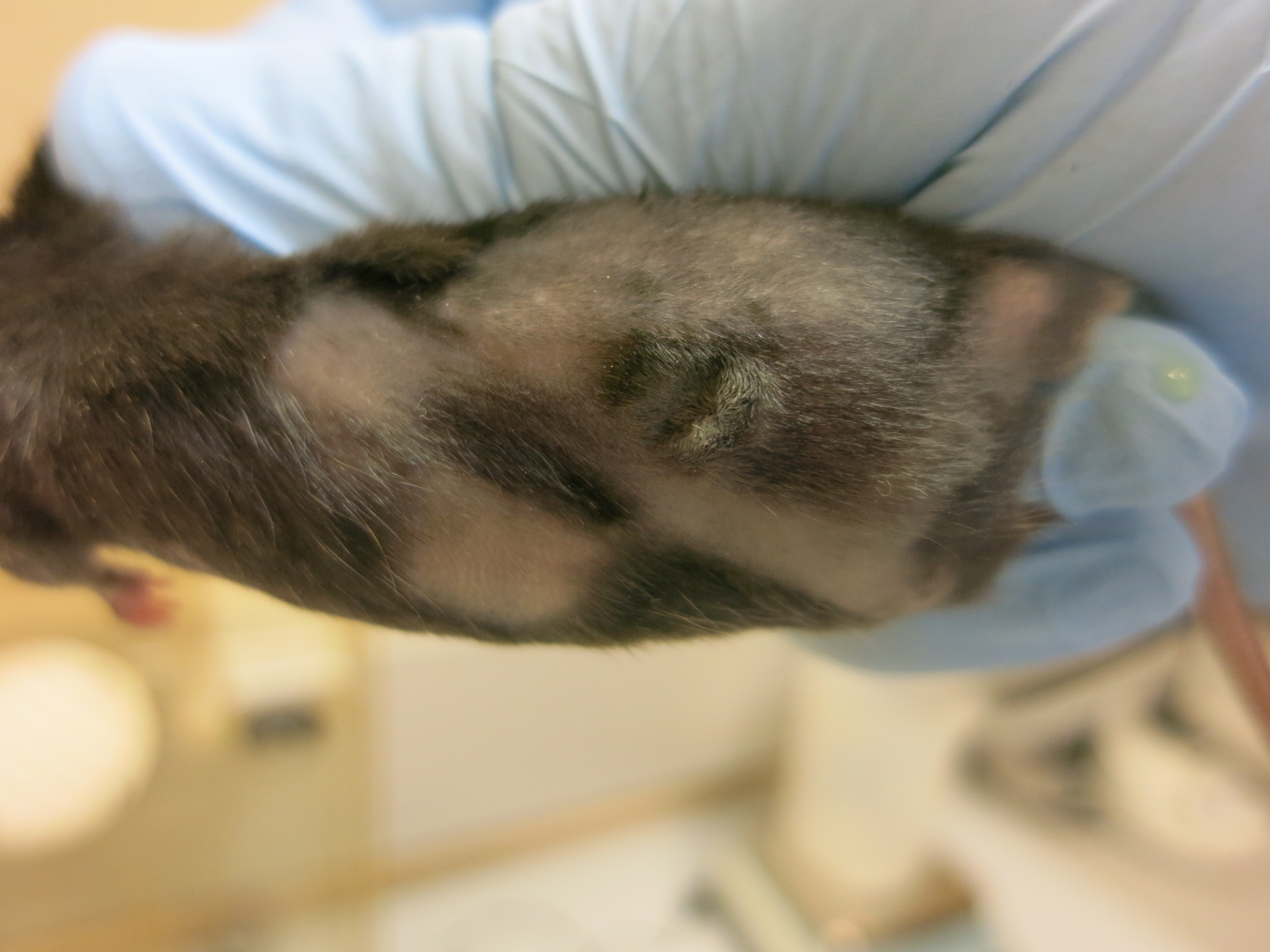

Supplement: Supplementary file 3 — Source Data for Expanded View [file EMMM-12-e11223-s009.zip › EV_source-data/Fig.EV5/combo d15.JPG]

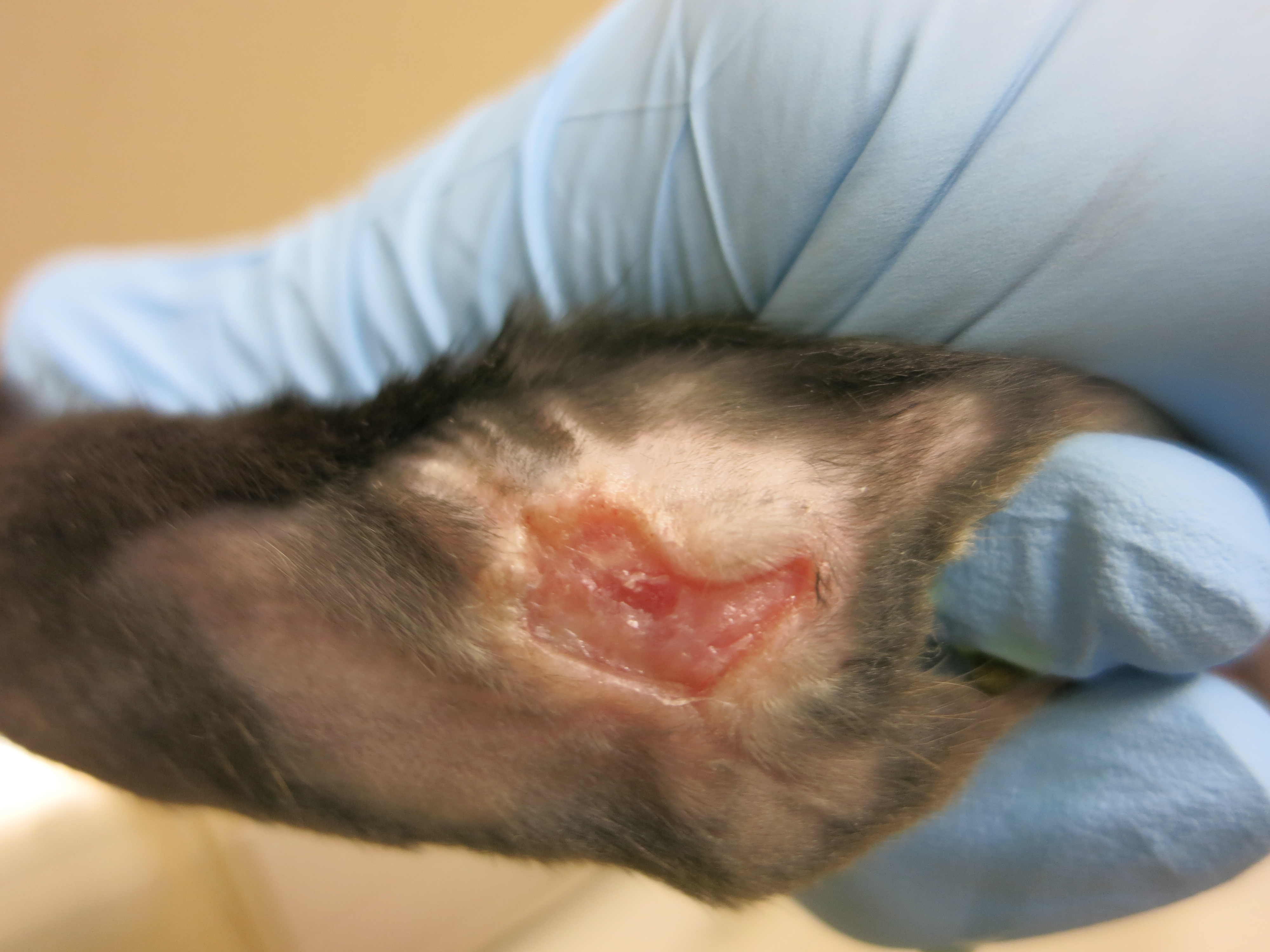

Supplement: Supplementary file 3 — Source Data for Expanded View [file EMMM-12-e11223-s009.zip › EV_source-data/Fig.EV5/combo d26.JPG]

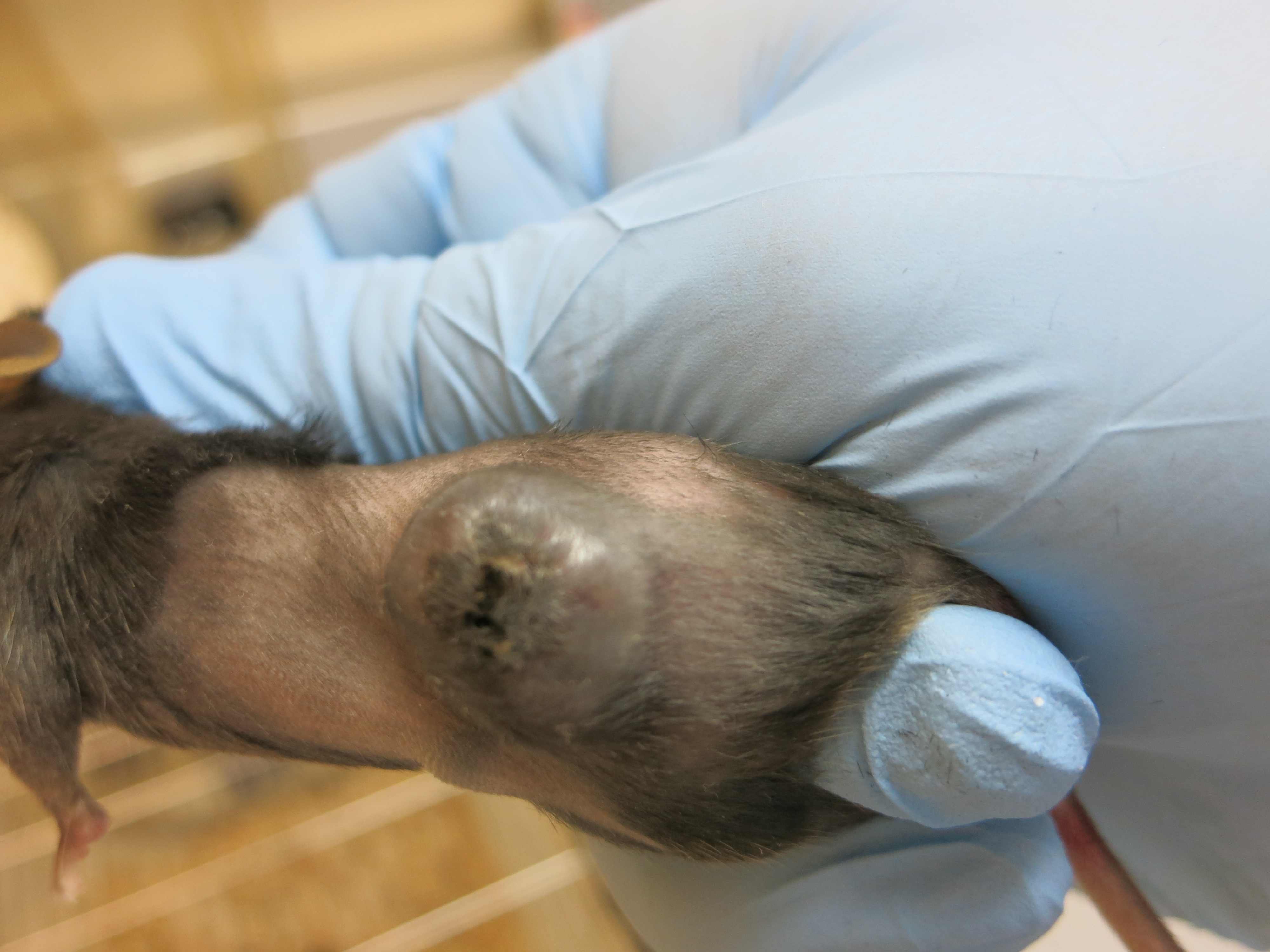

Supplement: Supplementary file 3 — Source Data for Expanded View [file EMMM-12-e11223-s009.zip › EV_source-data/Fig.EV5/PBS d15.JPG]

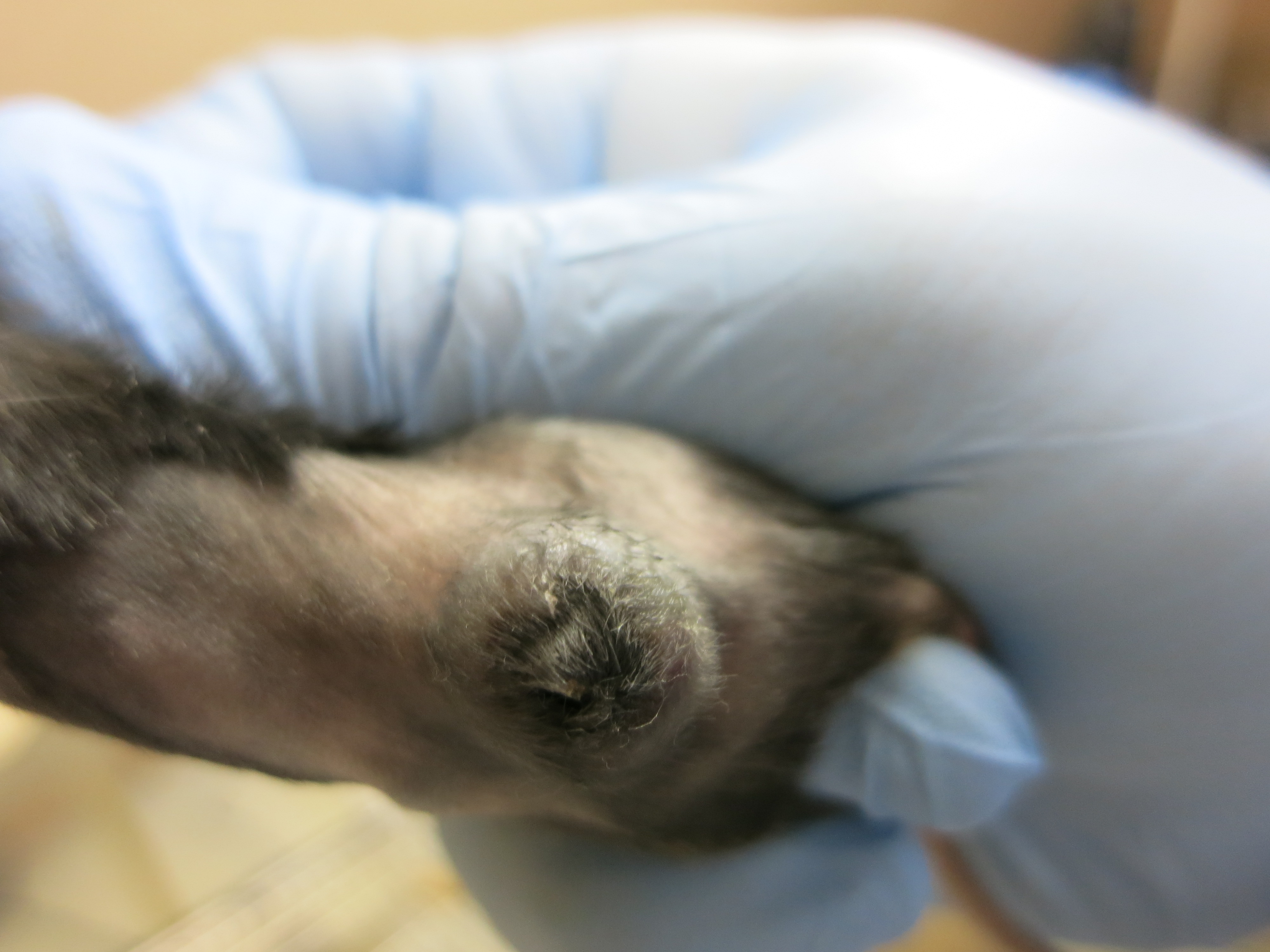

Supplement: Supplementary file 3 — Source Data for Expanded View [file EMMM-12-e11223-s009.zip › EV_source-data/Fig.EV5/PBS d14.JPG]

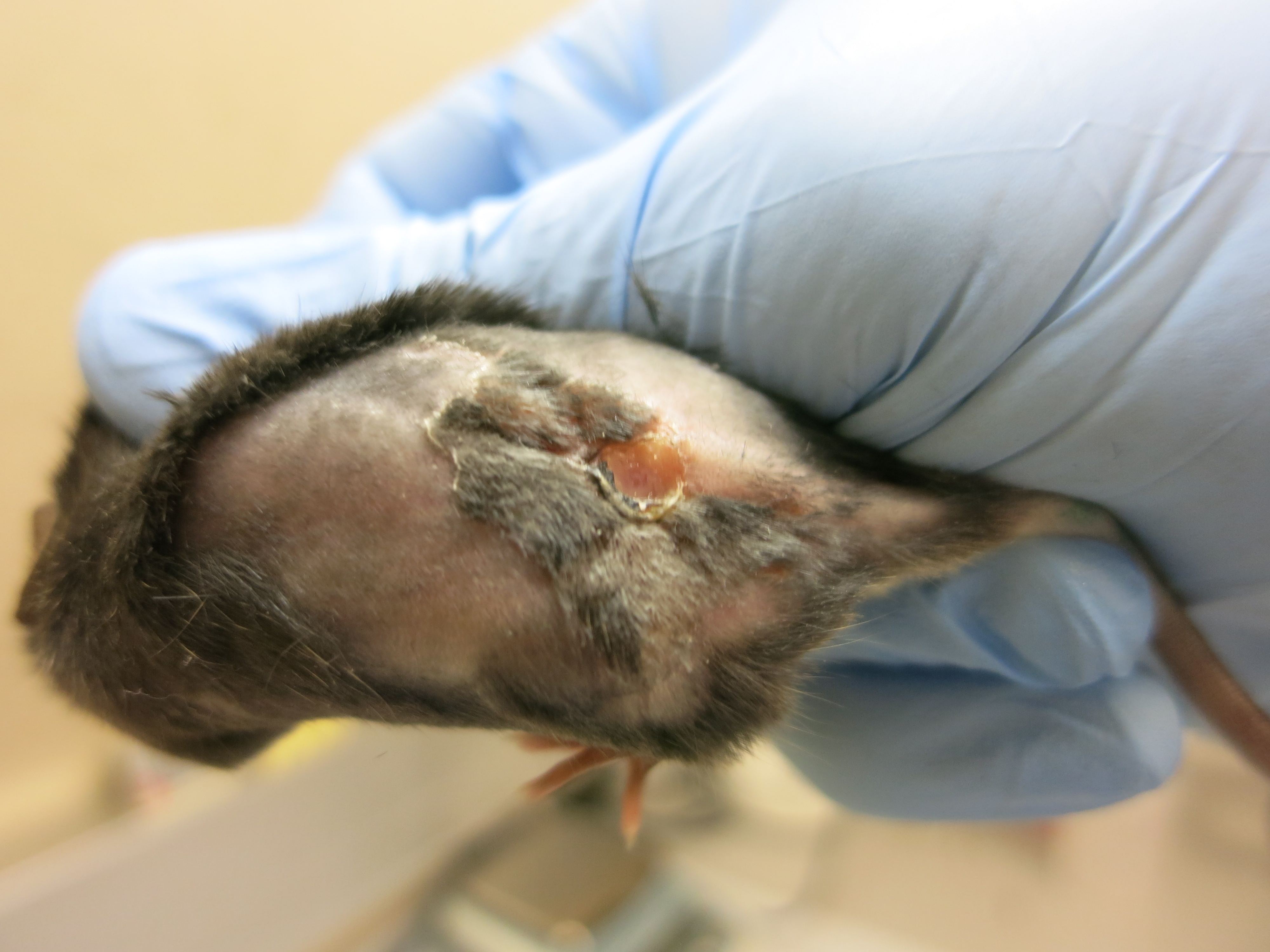

Supplement: Supplementary file 3 — Source Data for Expanded View [file EMMM-12-e11223-s009.zip › EV_source-data/Fig.EV5/combo d21.JPG]

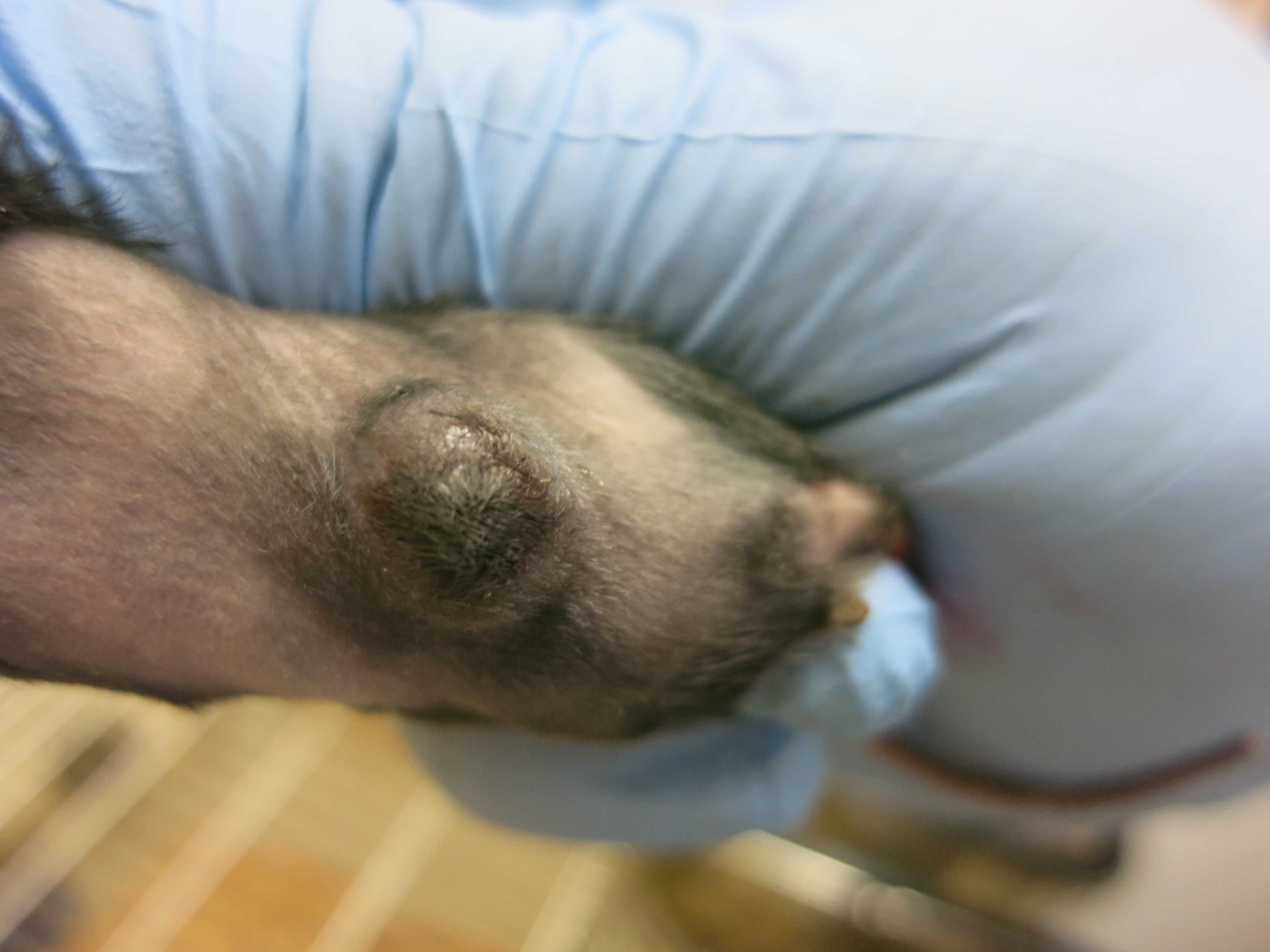

Supplement: Supplementary file 3 — Source Data for Expanded View [file EMMM-12-e11223-s009.zip › EV_source-data/Fig.EV5/PBS d10.JPG]

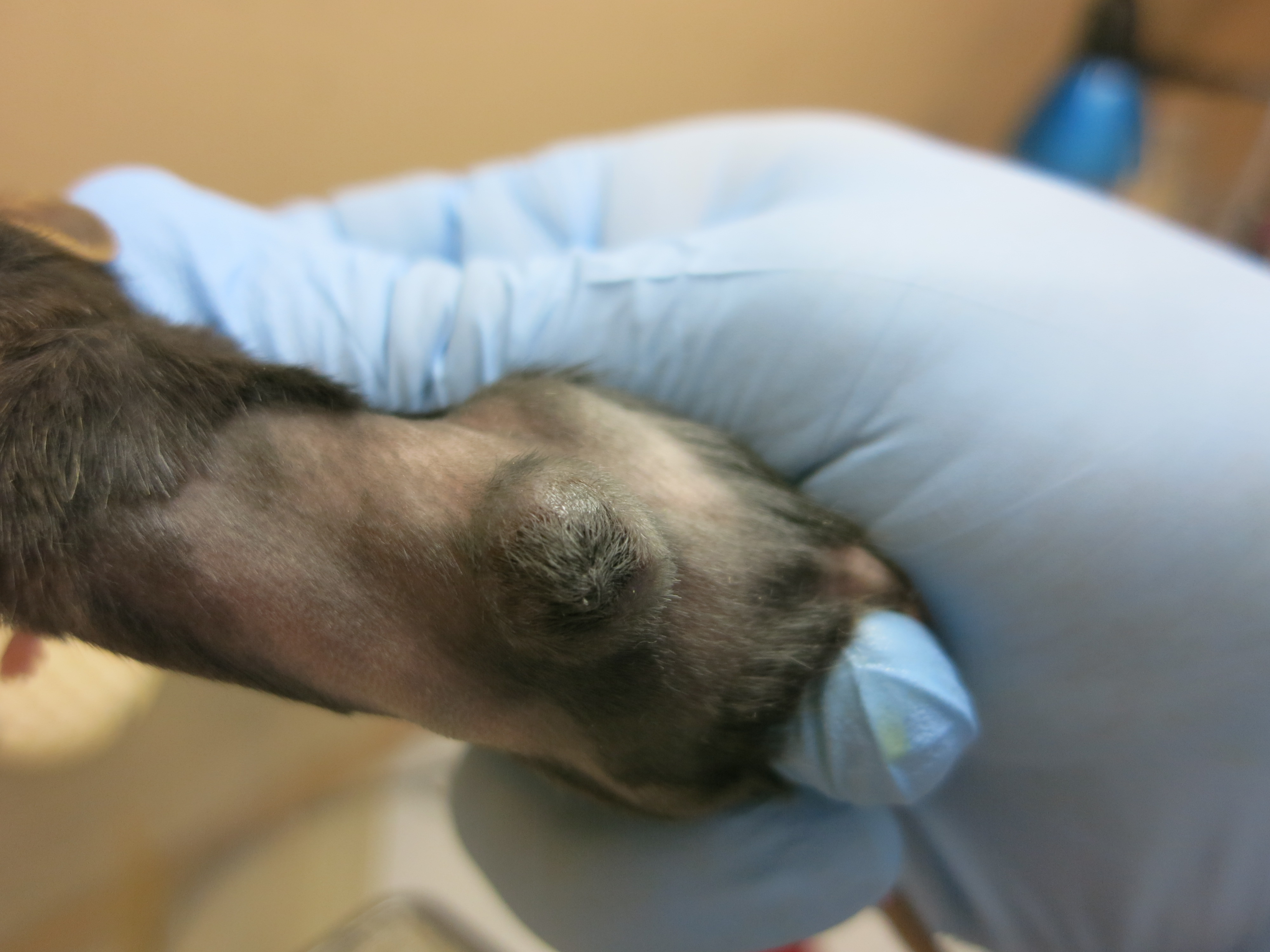

Supplement: Supplementary file 3 — Source Data for Expanded View [file EMMM-12-e11223-s009.zip › EV_source-data/Fig.EV5/PBS d11.JPG]

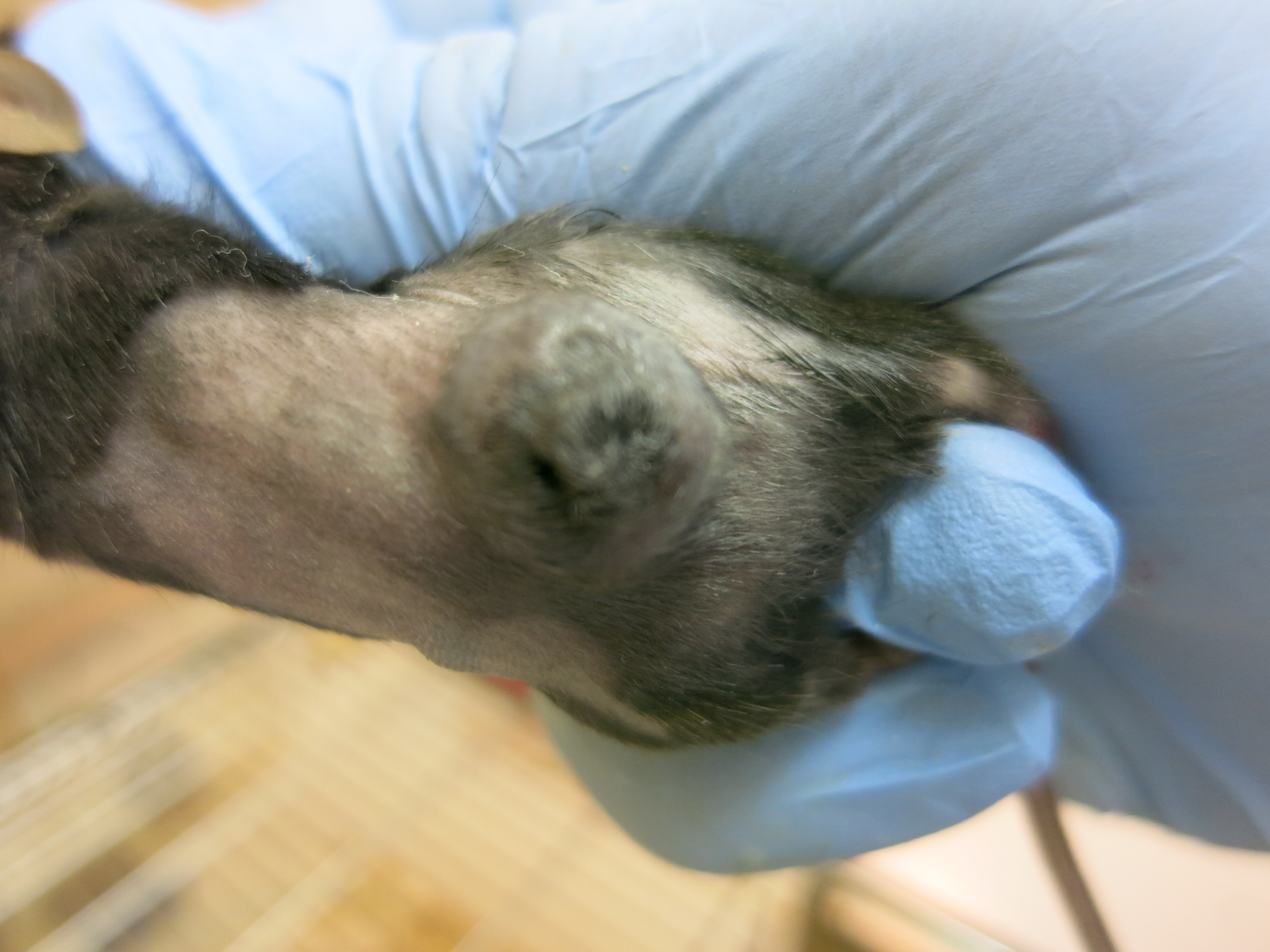

Supplement: Supplementary file 3 — Source Data for Expanded View [file EMMM-12-e11223-s009.zip › EV_source-data/Fig.EV5/PBS d13.JPG]

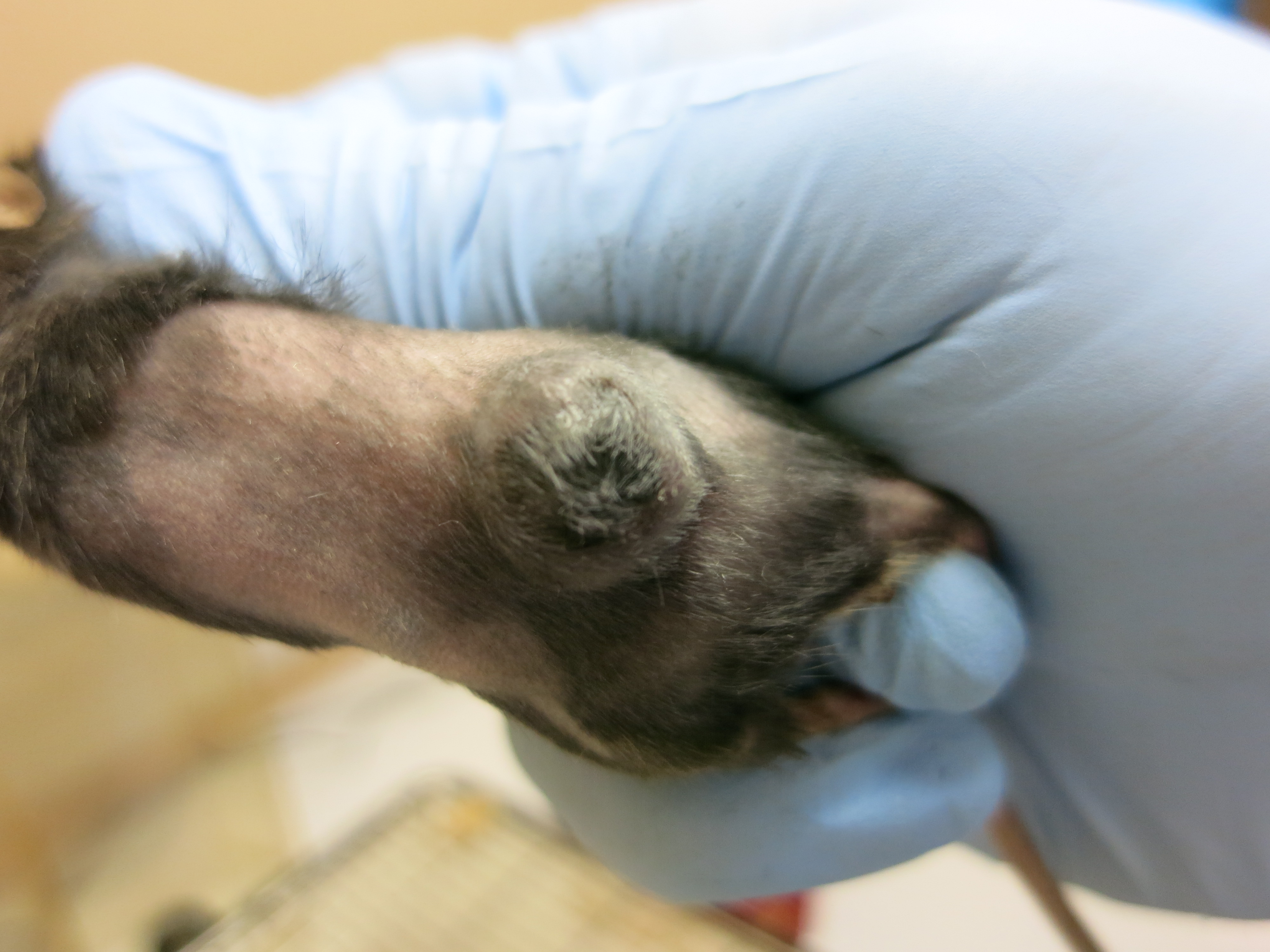

Supplement: Supplementary file 3 — Source Data for Expanded View [file EMMM-12-e11223-s009.zip › EV_source-data/Fig.EV5/PBS d12.JPG]

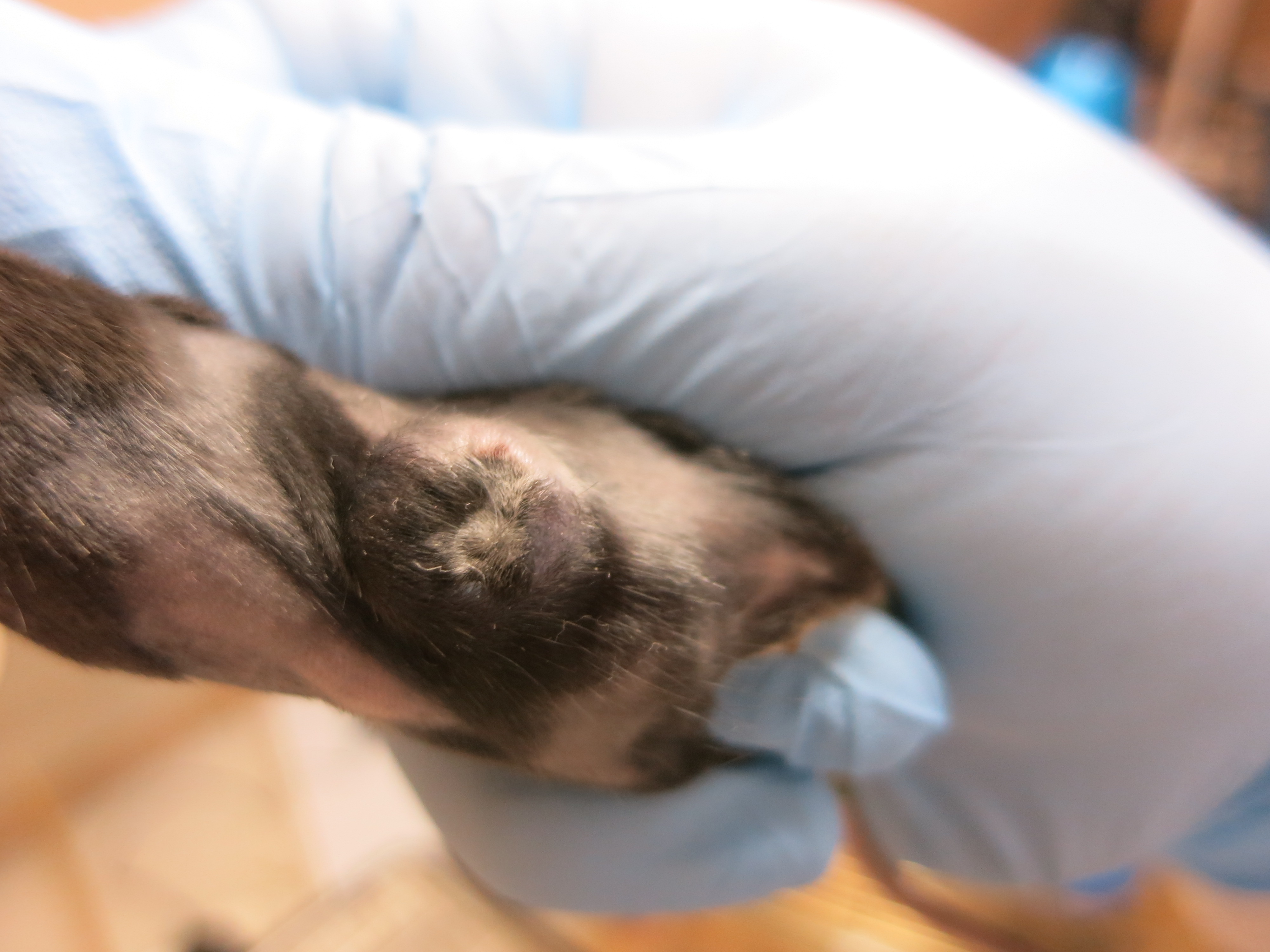

Supplement: Supplementary file 3 — Source Data for Expanded View [file EMMM-12-e11223-s009.zip › EV_source-data/Fig.EV5/AFR d14.JPG]

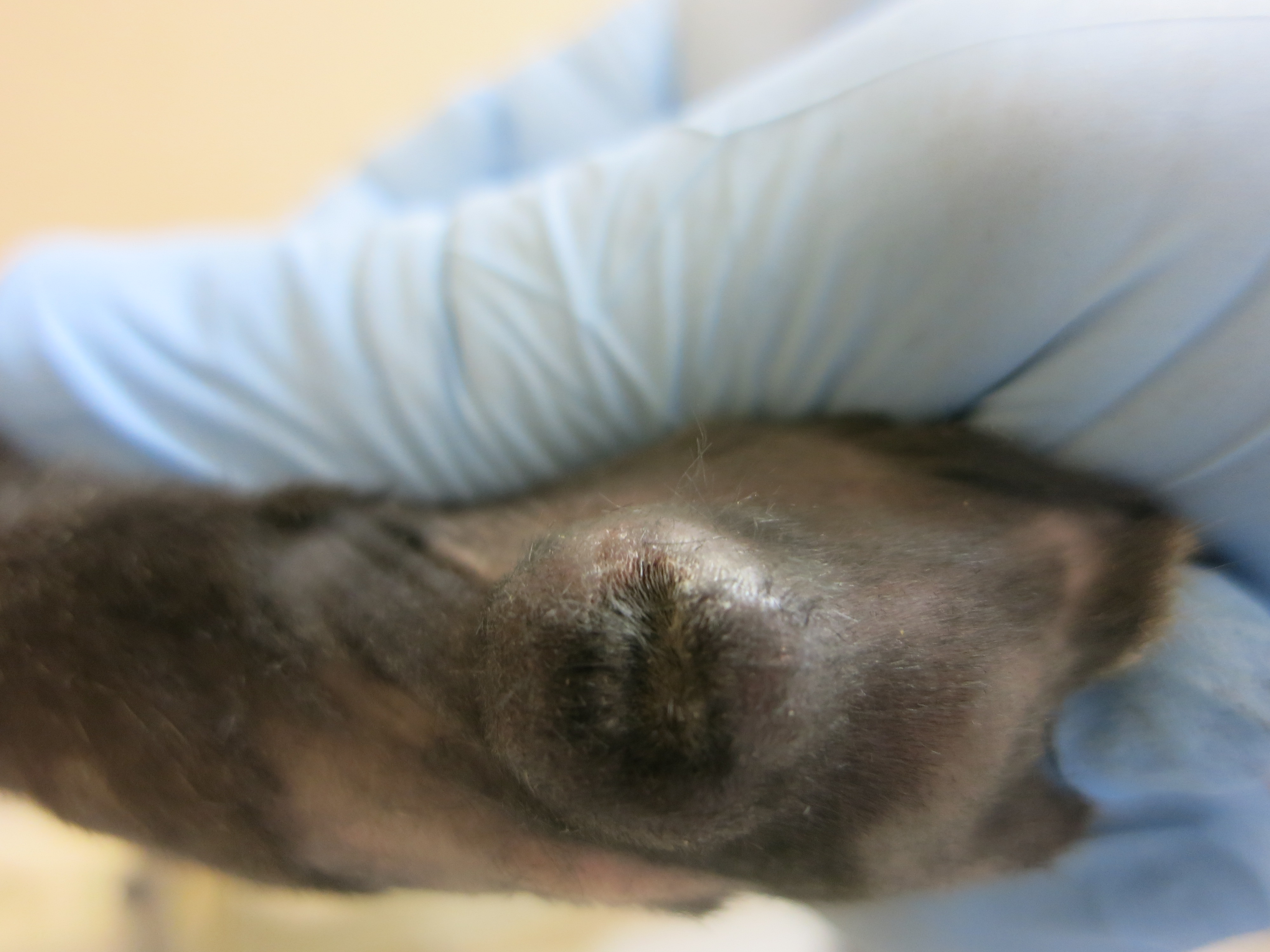

Supplement: Supplementary file 3 — Source Data for Expanded View [file EMMM-12-e11223-s009.zip › EV_source-data/Fig.EV5/AFR d15.JPG]

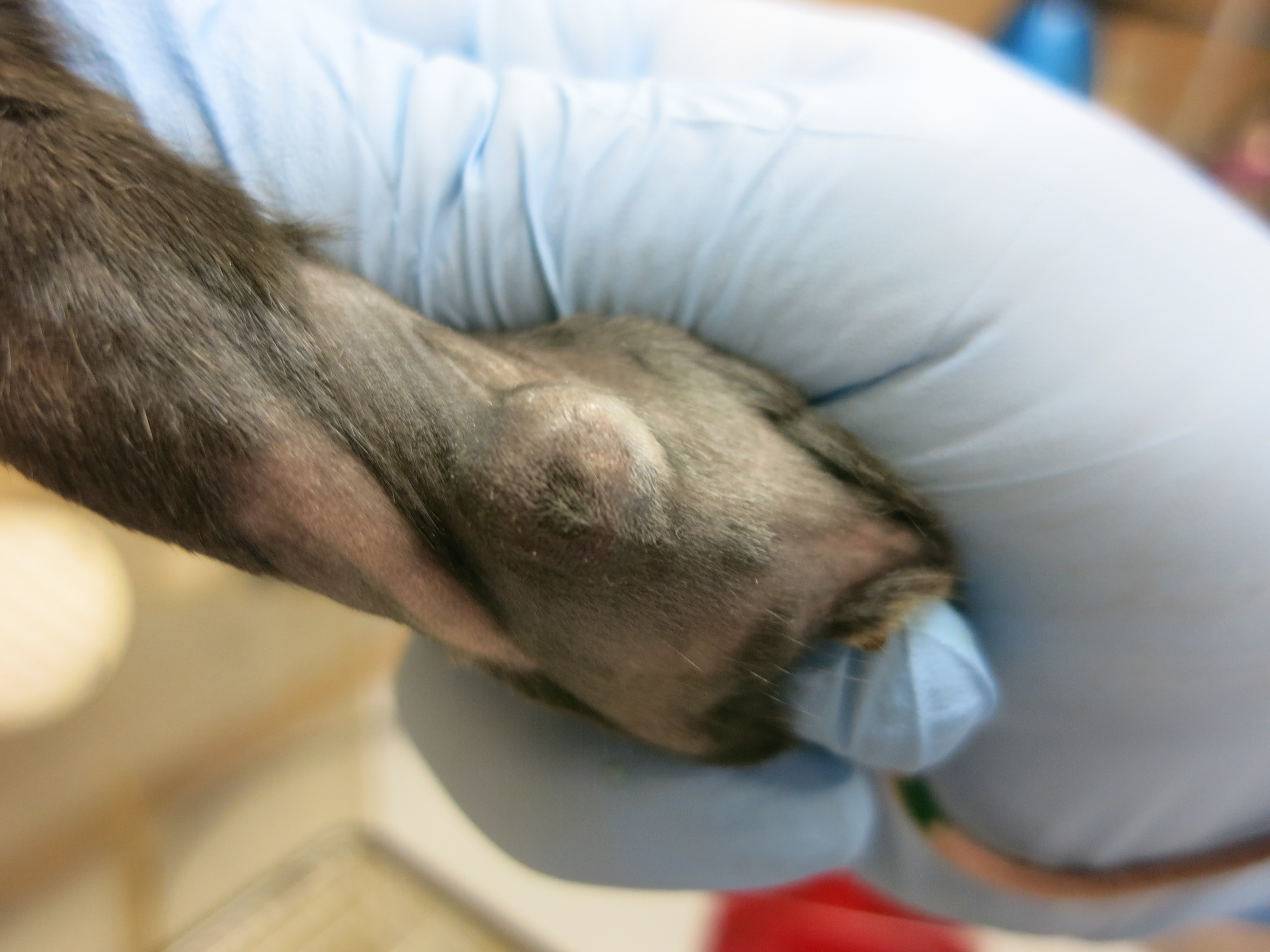

Supplement: Supplementary file 3 — Source Data for Expanded View [file EMMM-12-e11223-s009.zip › EV_source-data/Fig.EV5/AFR d11.JPG]

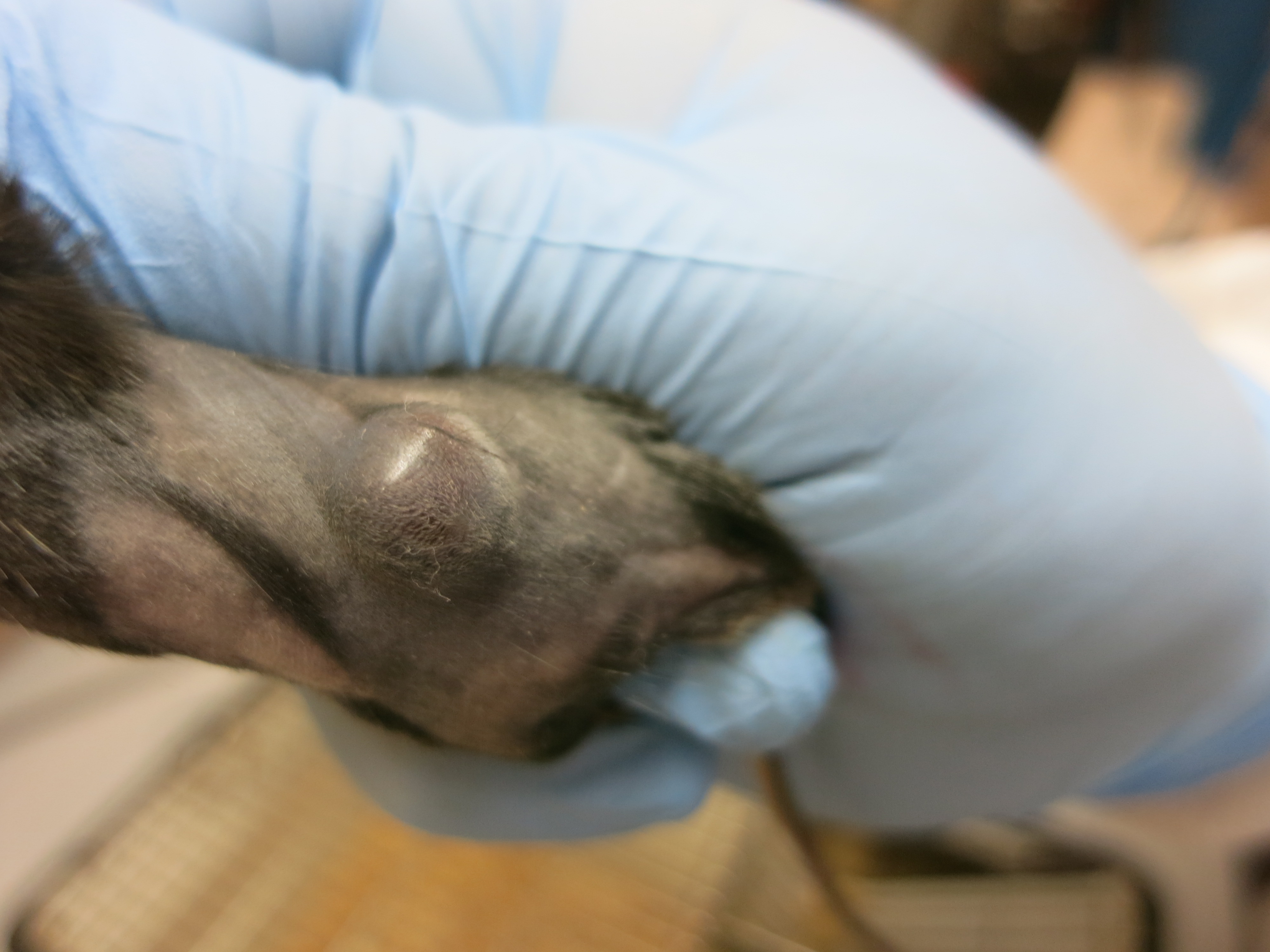

Supplement: Supplementary file 3 — Source Data for Expanded View [file EMMM-12-e11223-s009.zip › EV_source-data/Fig.EV5/AFR d10.JPG]

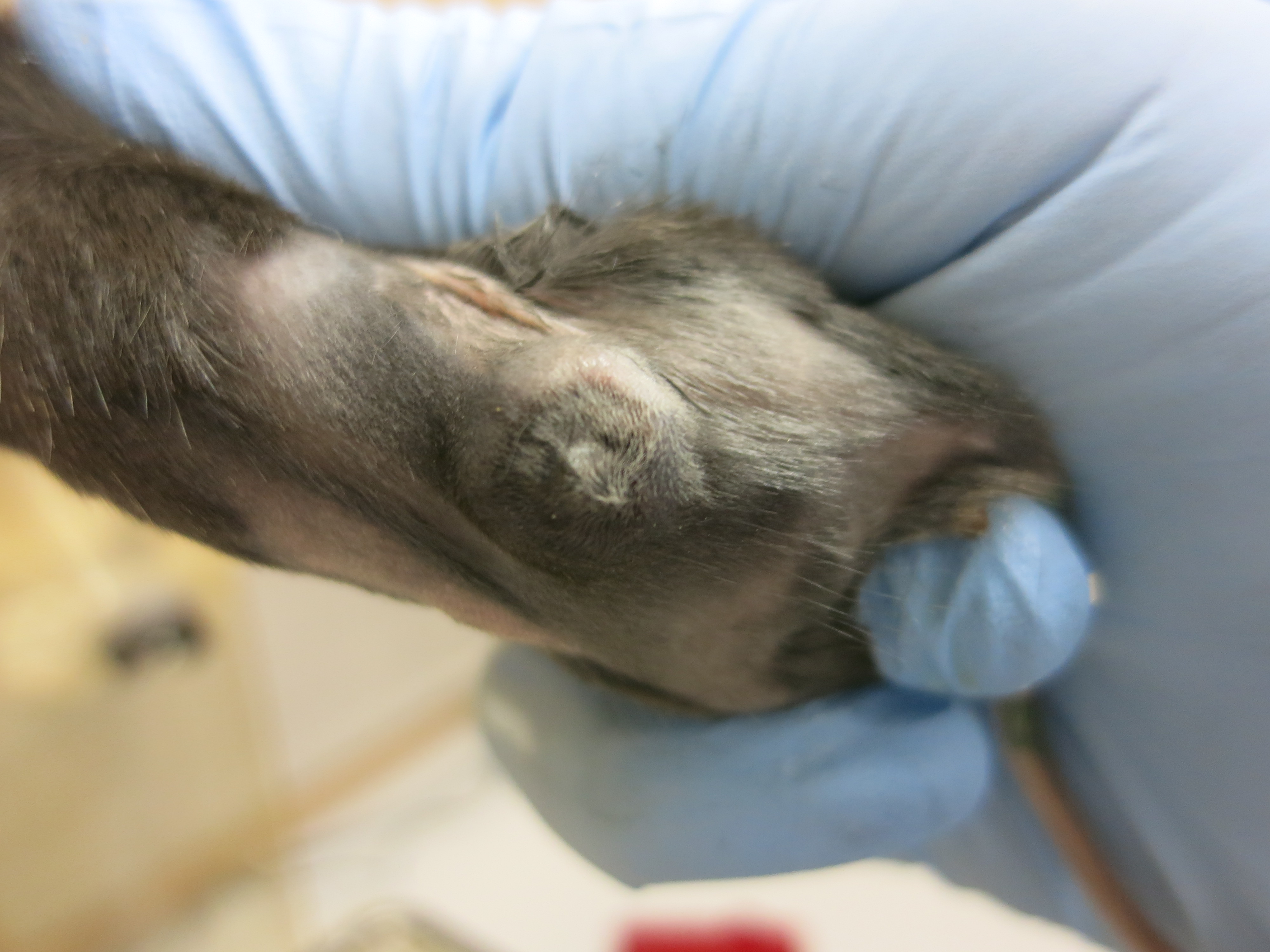

Supplement: Supplementary file 3 — Source Data for Expanded View [file EMMM-12-e11223-s009.zip › EV_source-data/Fig.EV5/AFR d12.JPG]

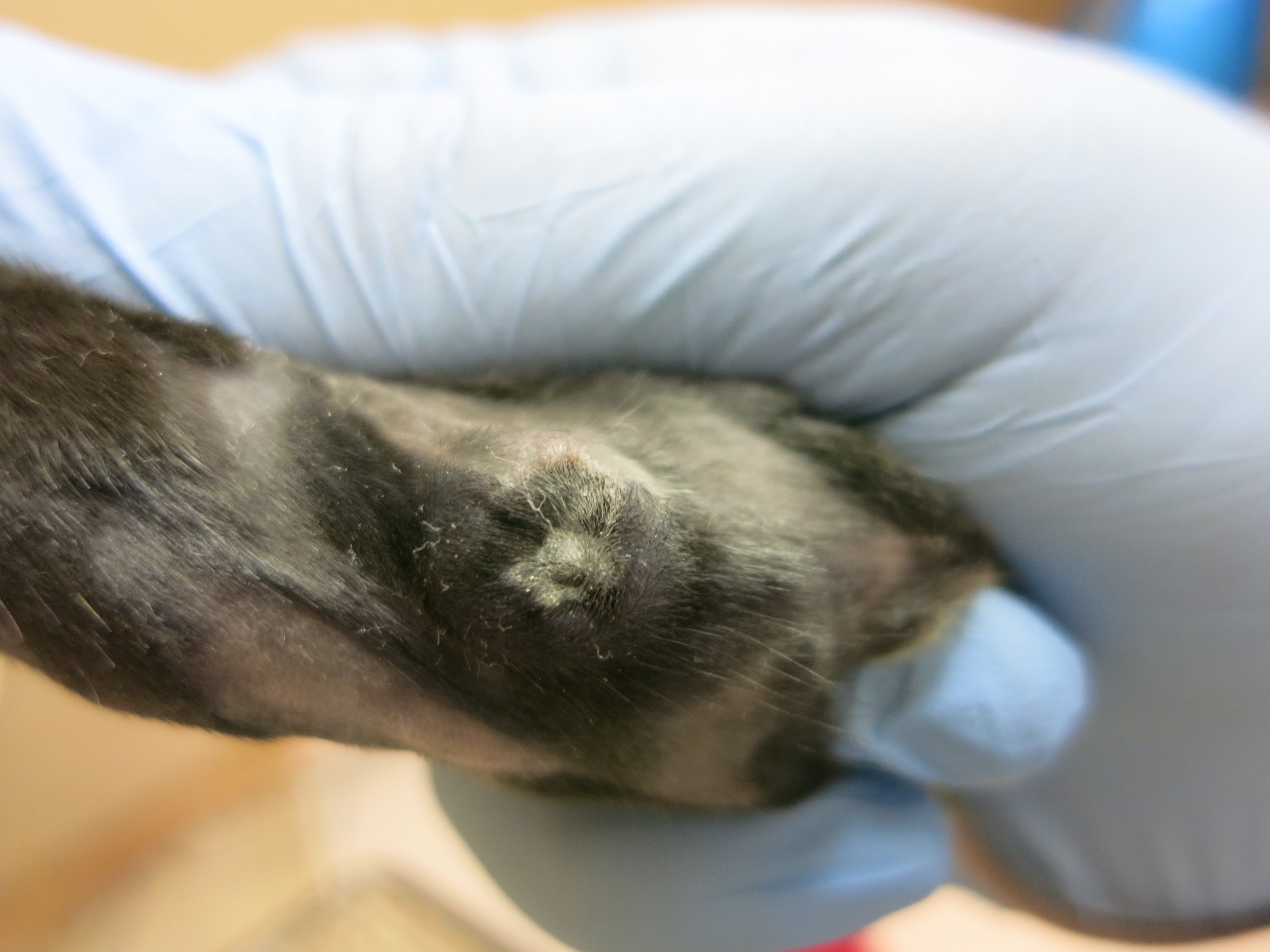

Supplement: Supplementary file 3 — Source Data for Expanded View [file EMMM-12-e11223-s009.zip › EV_source-data/Fig.EV5/AFR d13.JPG]

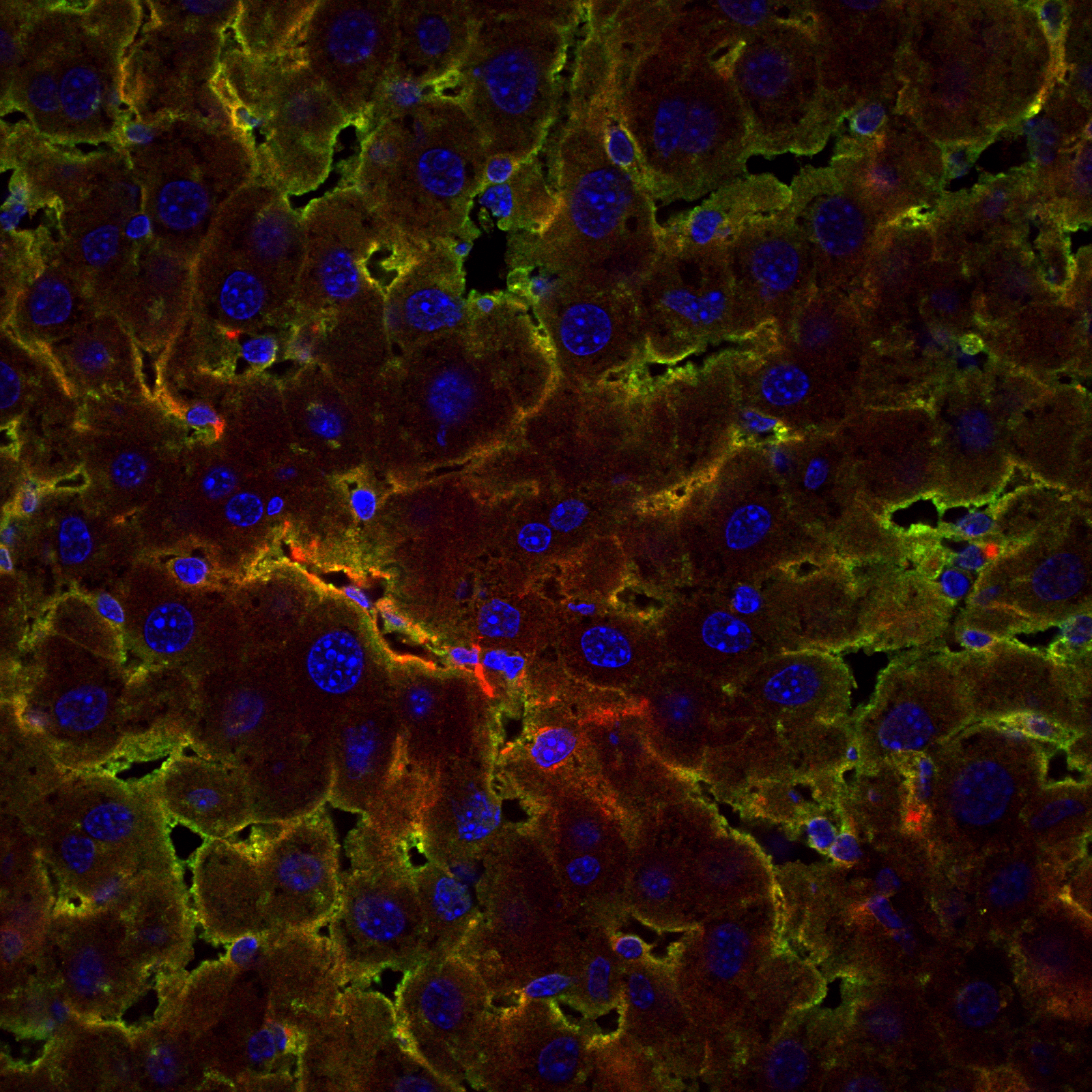

Supplement: Supplementary file 3 — Source Data for Expanded View [file EMMM-12-e11223-s009.zip › EV_source-data/Fig.EV2D/Liver CD31_.tif]

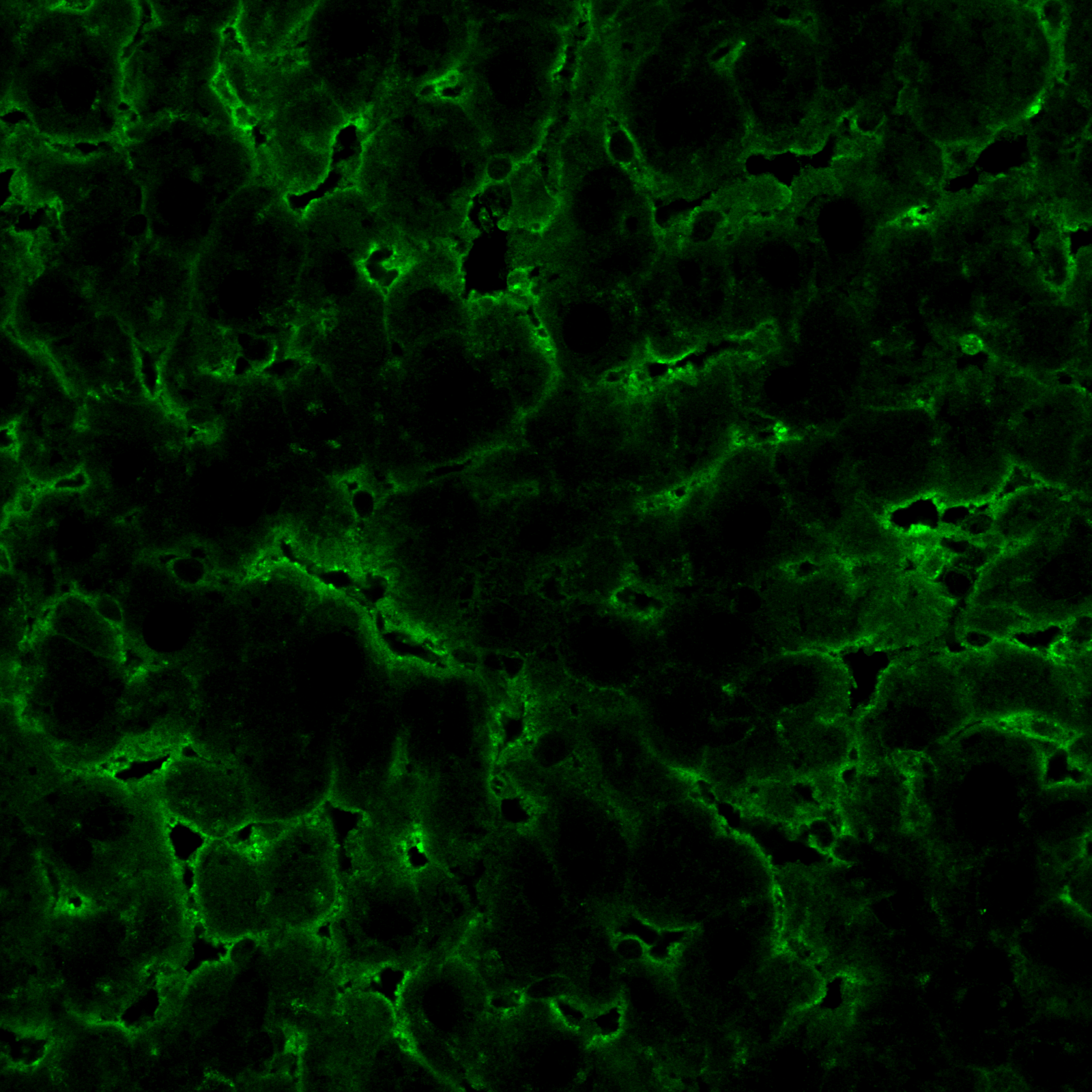

Supplement: Supplementary file 3 — Source Data for Expanded View [file EMMM-12-e11223-s009.zip › EV_source-data/Fig.EV2D/Liver CD31_C002.tif]

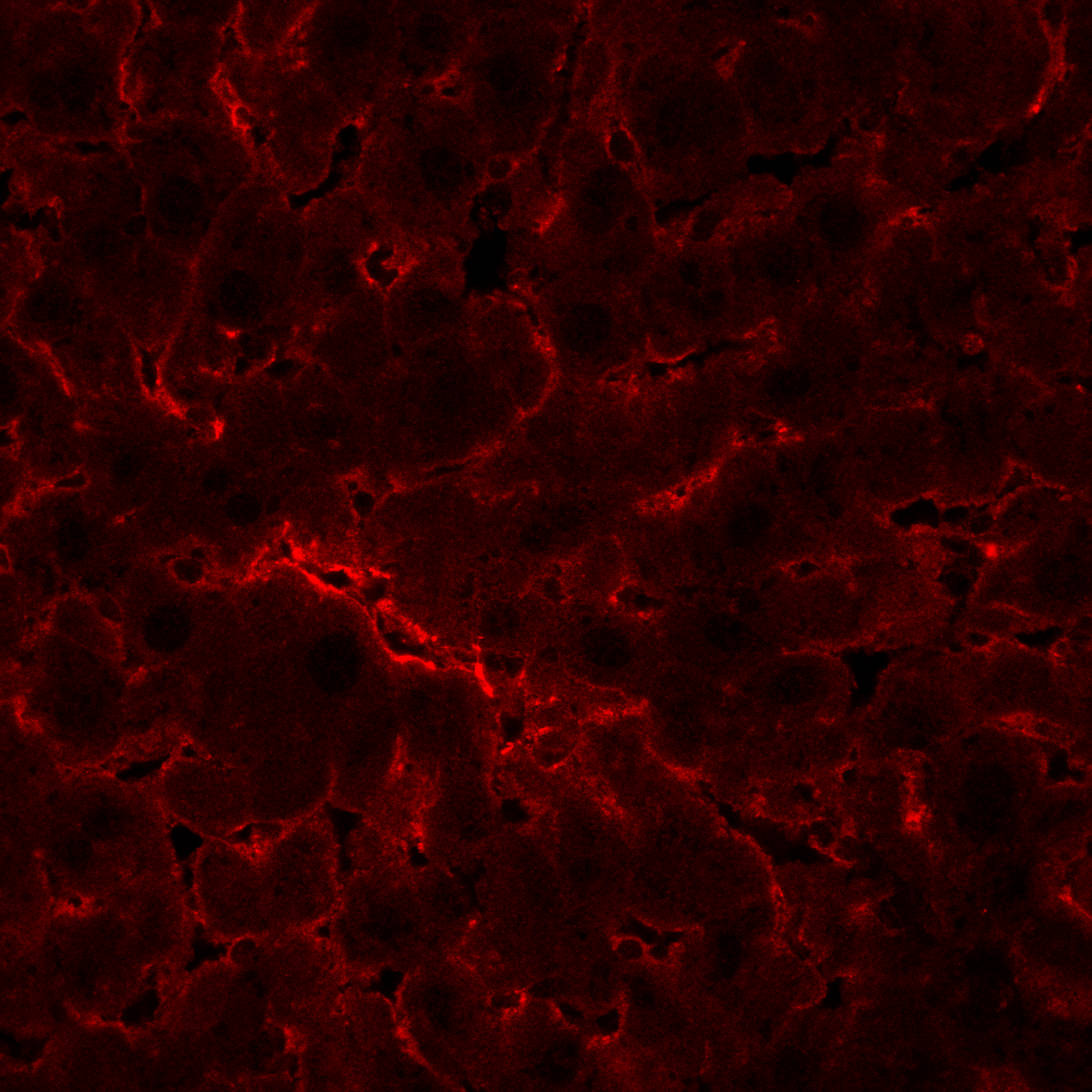

Supplement: Supplementary file 3 — Source Data for Expanded View [file EMMM-12-e11223-s009.zip › EV_source-data/Fig.EV2D/Liver CD31_C003.tif]

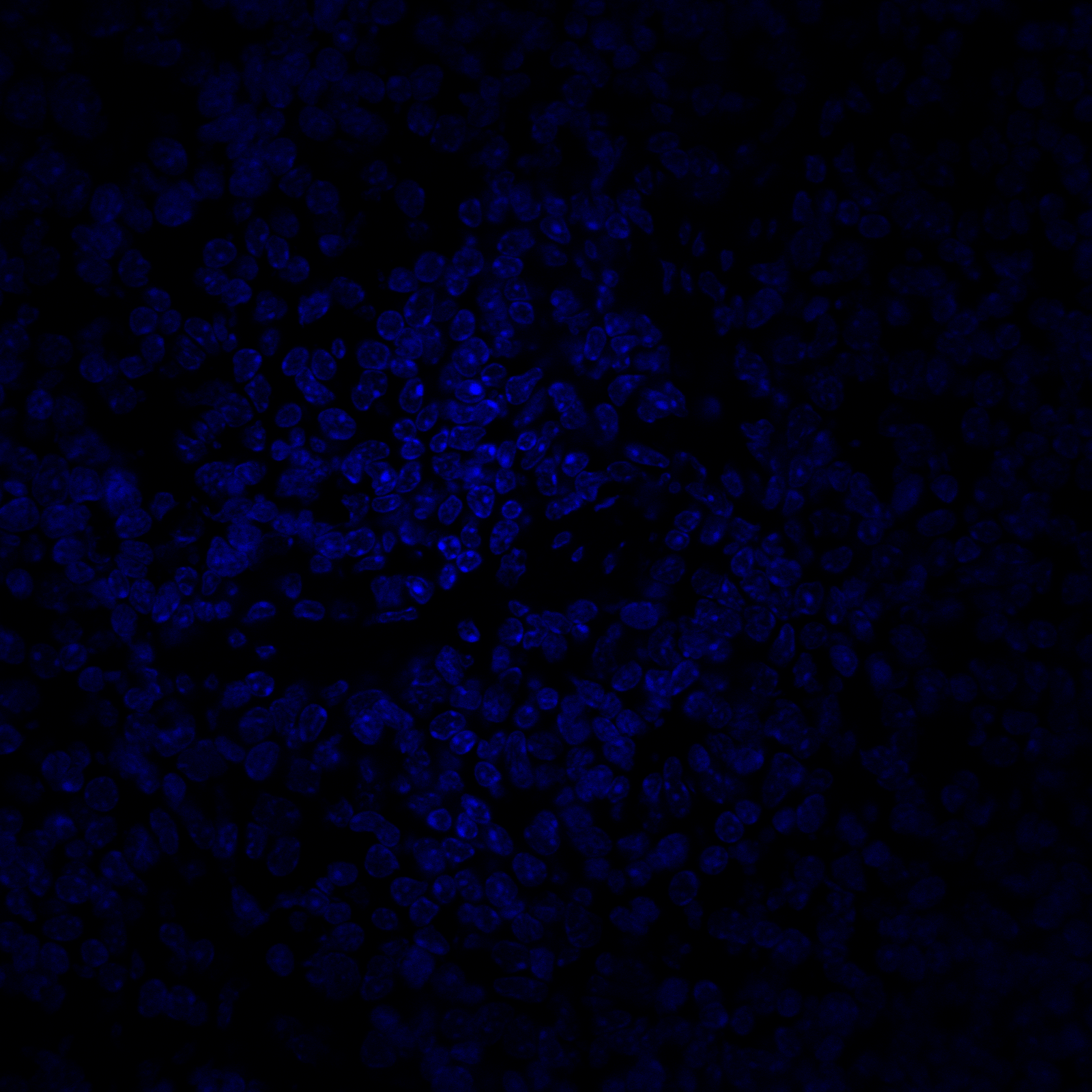

Supplement: Supplementary file 3 — Source Data for Expanded View [file EMMM-12-e11223-s009.zip › EV_source-data/Fig.EV2D/Spleen CD31_C001.tif]

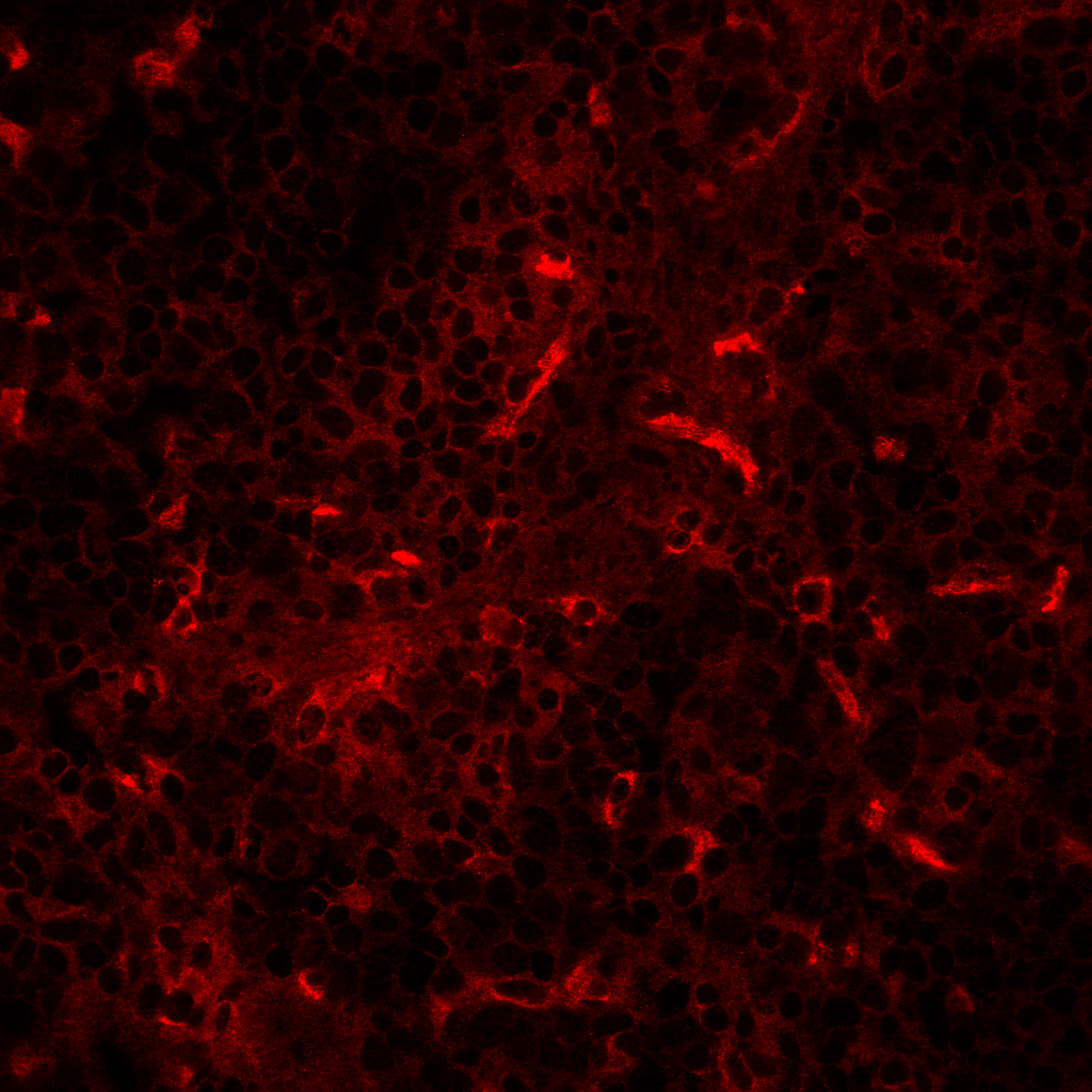

Supplement: Supplementary file 3 — Source Data for Expanded View [file EMMM-12-e11223-s009.zip › EV_source-data/Fig.EV2D/Spleen CD31_C003.tif]

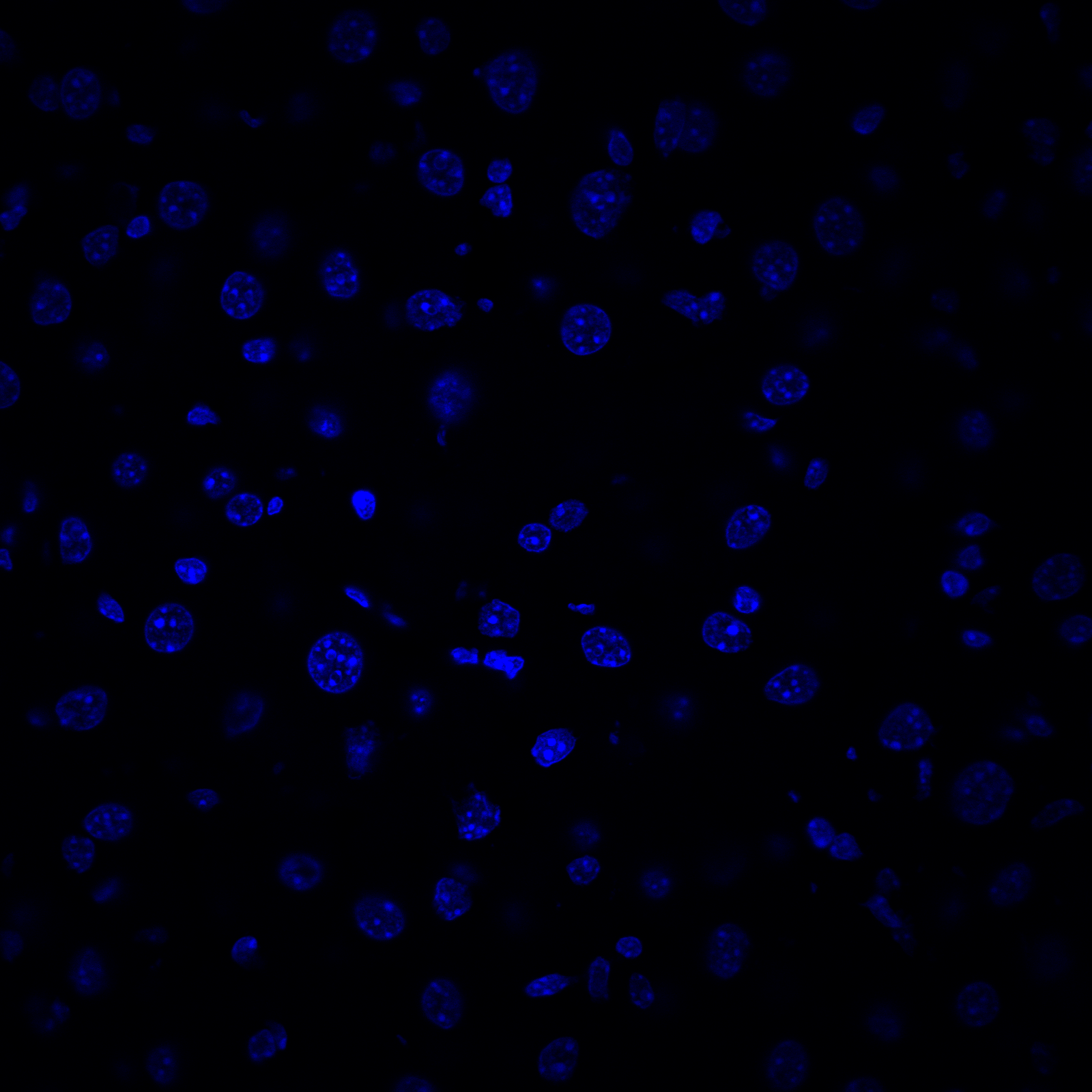

Supplement: Supplementary file 3 — Source Data for Expanded View [file EMMM-12-e11223-s009.zip › EV_source-data/Fig.EV2D/Liver CD31_C001.tif]

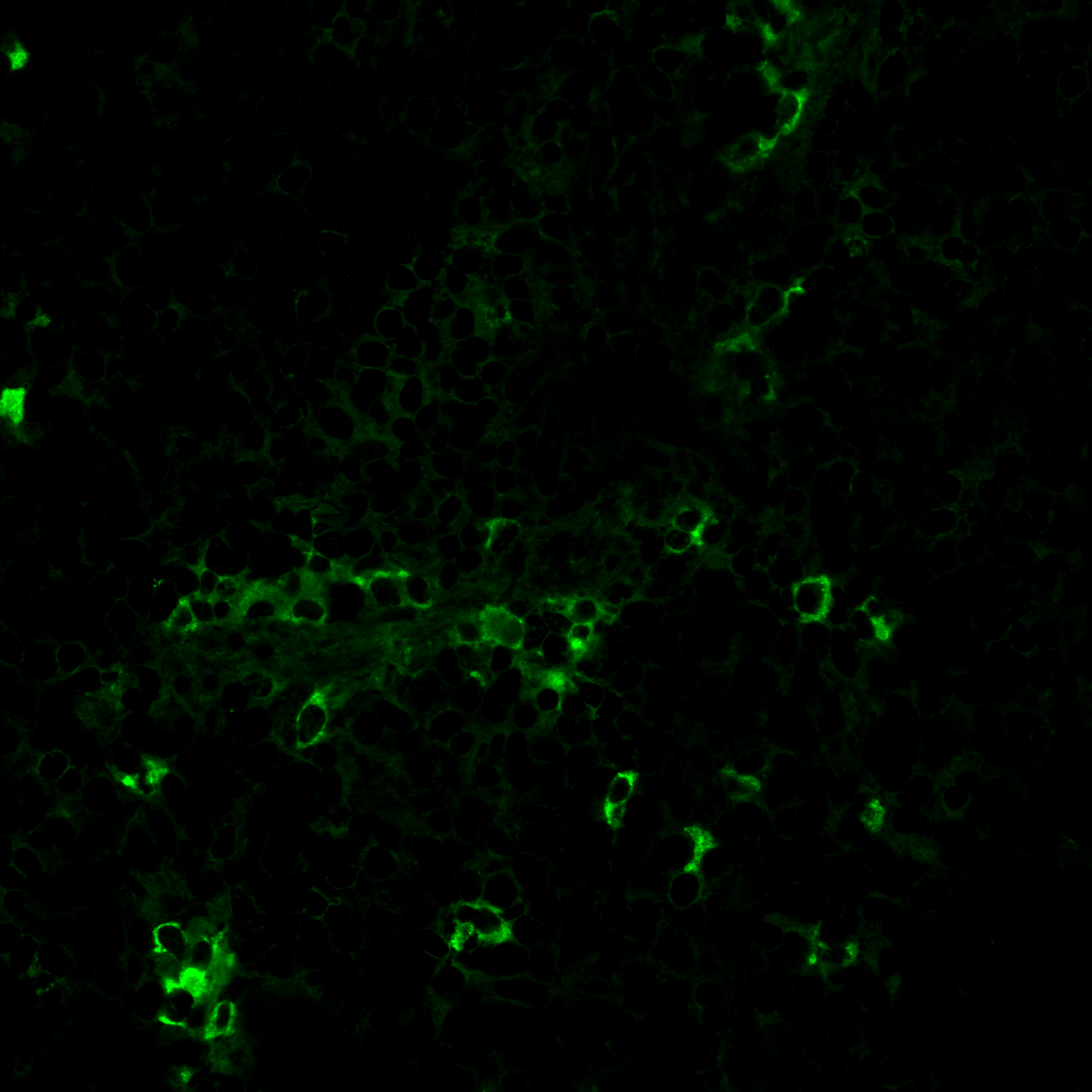

Supplement: Supplementary file 3 — Source Data for Expanded View [file EMMM-12-e11223-s009.zip › EV_source-data/Fig.EV2D/Spleen CD31_C002.tif]

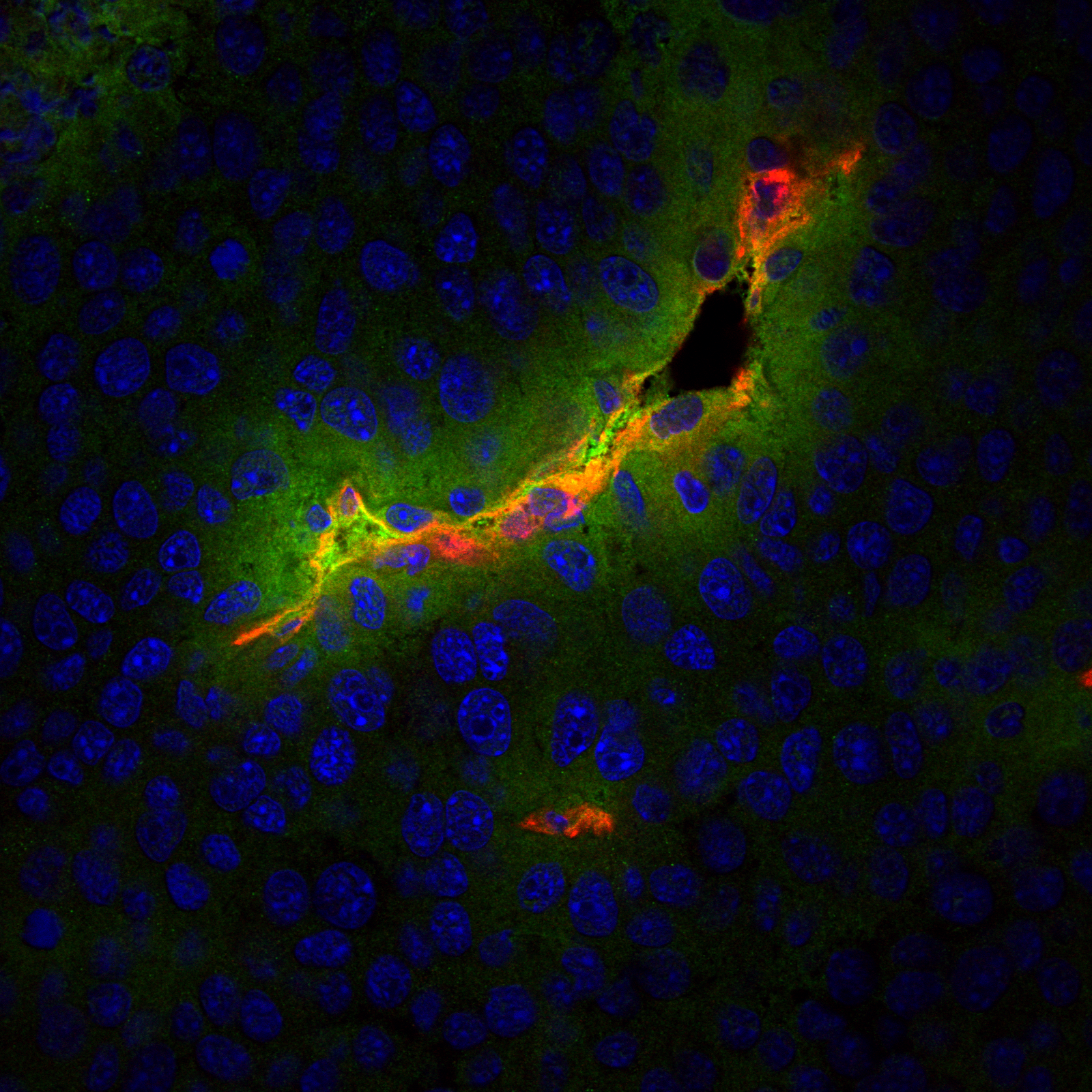

Supplement: Supplementary file 3 — Source Data for Expanded View [file EMMM-12-e11223-s009.zip › EV_source-data/Fig.EV2D/B16 CD31_b_.tif]

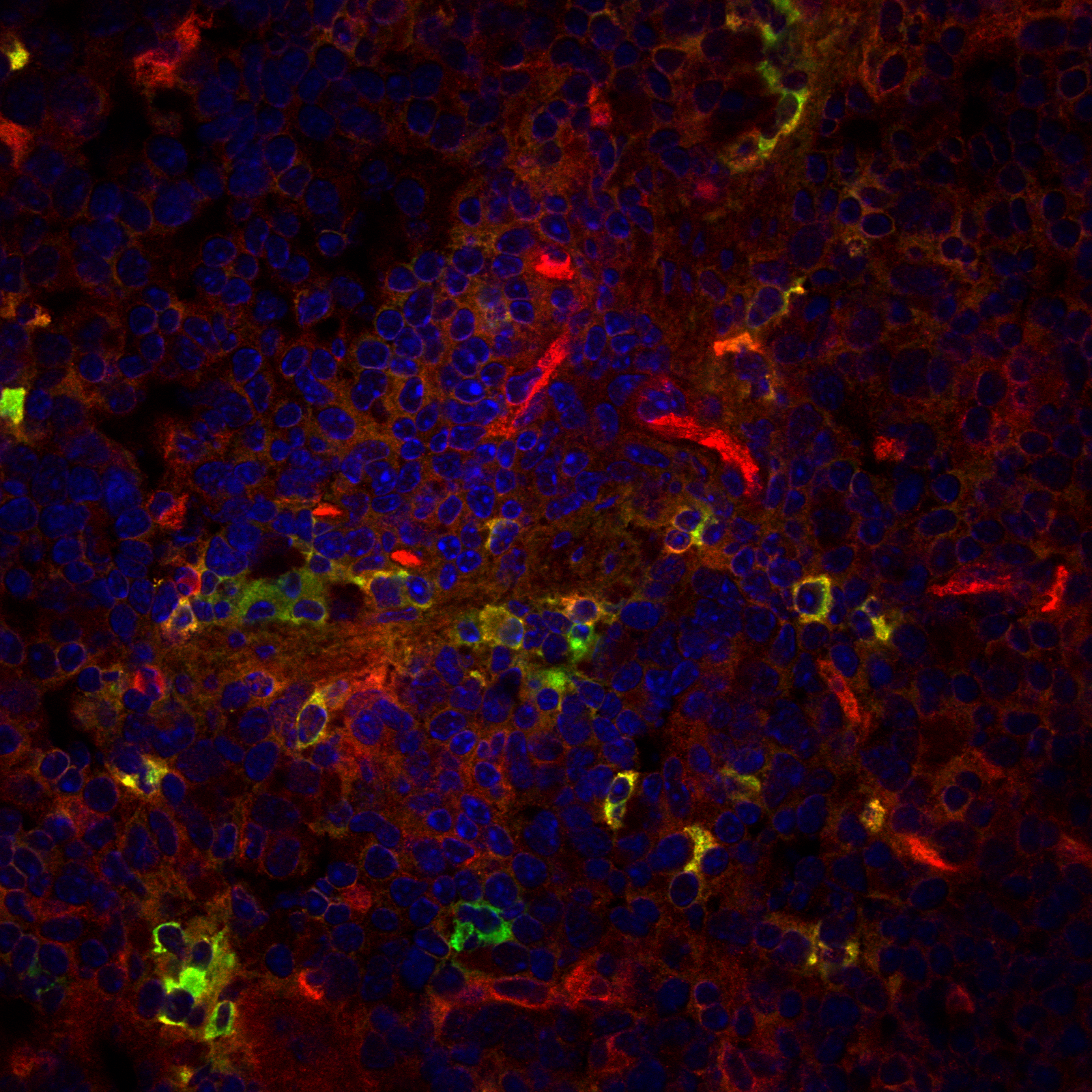

Supplement: Supplementary file 3 — Source Data for Expanded View [file EMMM-12-e11223-s009.zip › EV_source-data/Fig.EV2D/Spleen CD31_.tif]

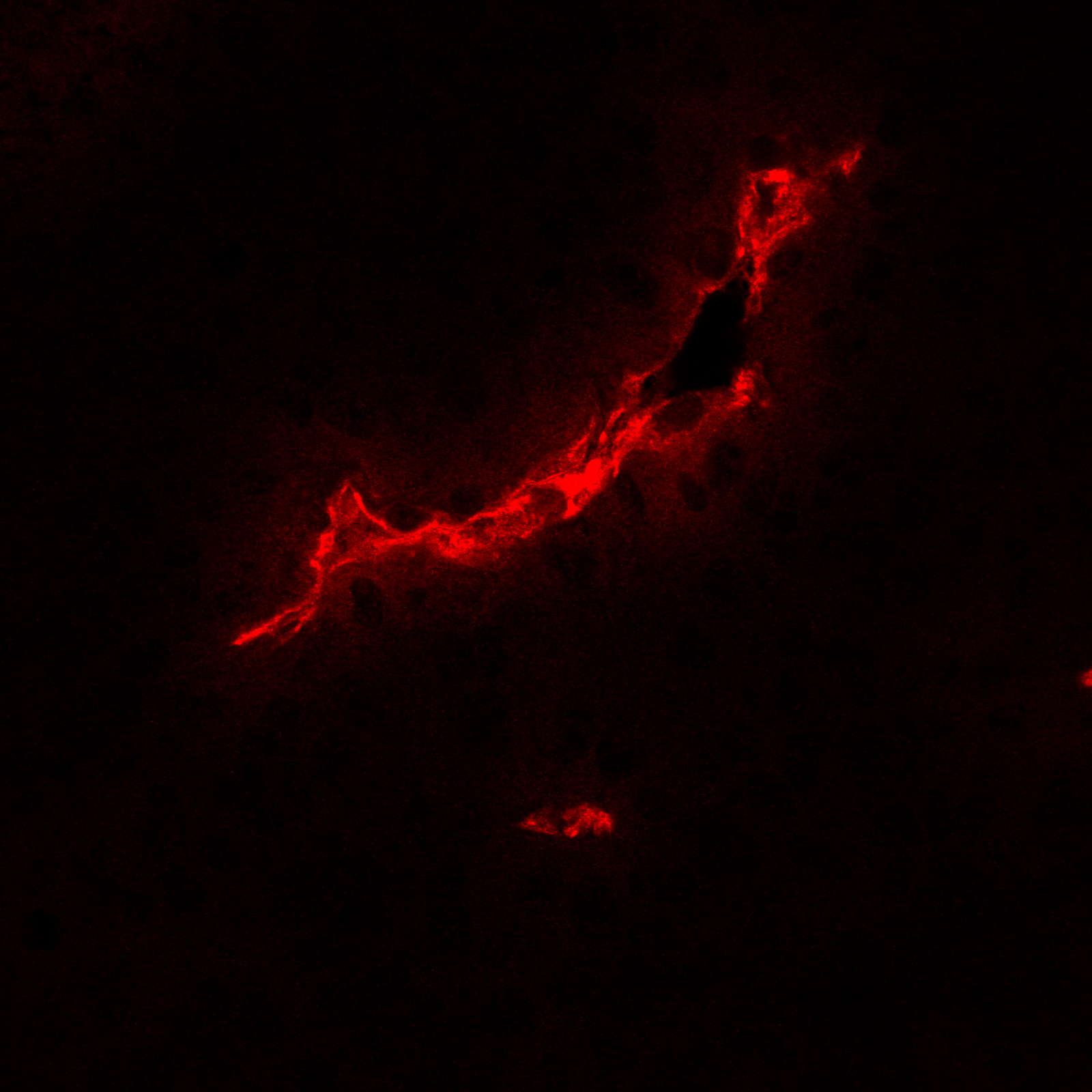

Supplement: Supplementary file 3 — Source Data for Expanded View [file EMMM-12-e11223-s009.zip › EV_source-data/Fig.EV2D/B16 CD31_b_C003.tif]

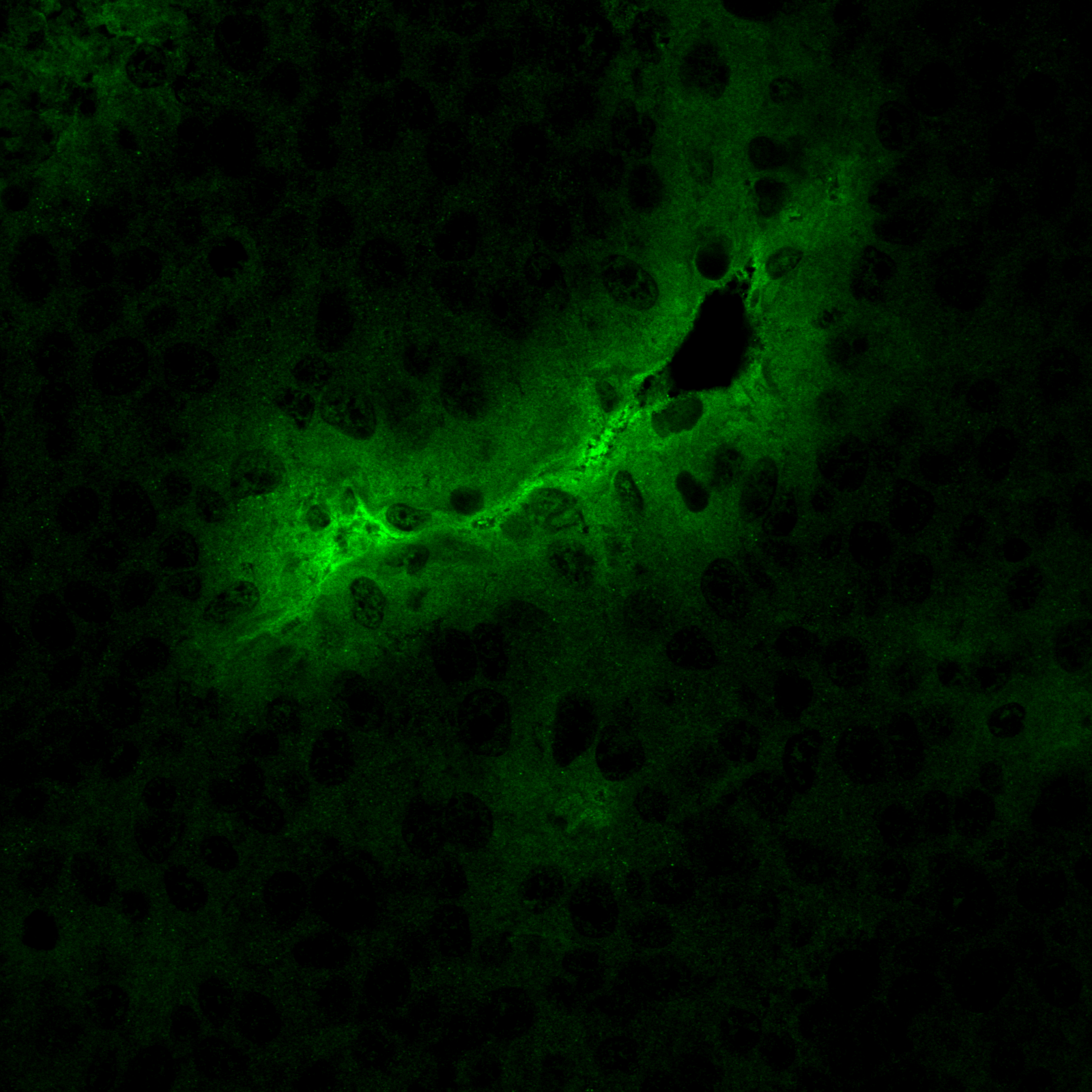

Supplement: Supplementary file 3 — Source Data for Expanded View [file EMMM-12-e11223-s009.zip › EV_source-data/Fig.EV2D/B16 CD31_b_C002.tif]

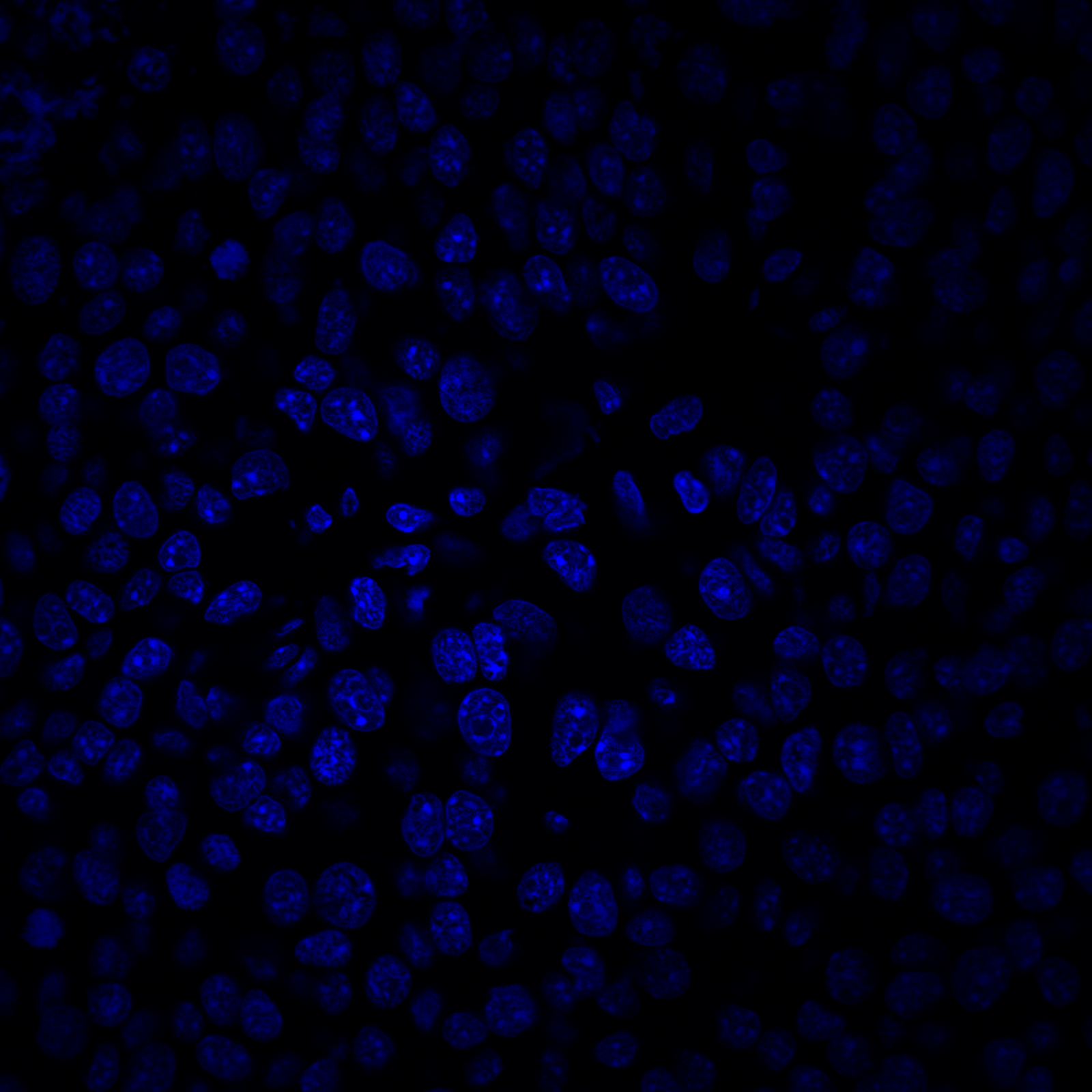

Supplement: Supplementary file 3 — Source Data for Expanded View [file EMMM-12-e11223-s009.zip › EV_source-data/Fig.EV2D/B16 CD31_b_C001.tif]

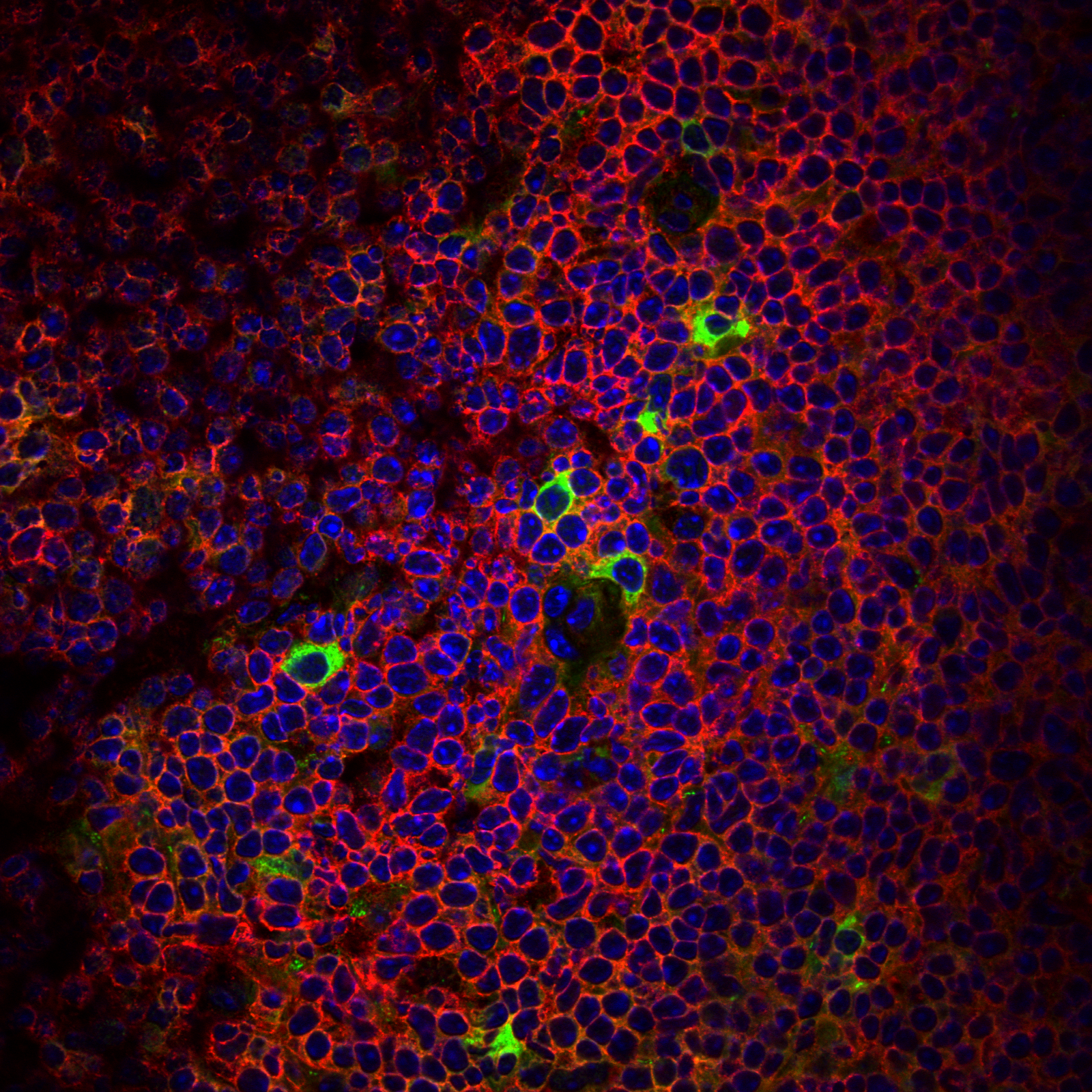

Supplement: Supplementary file 3 — Source Data for Expanded View [file EMMM-12-e11223-s009.zip › EV_source-data/Fig.EV2E/Spleen CD45_.tif]

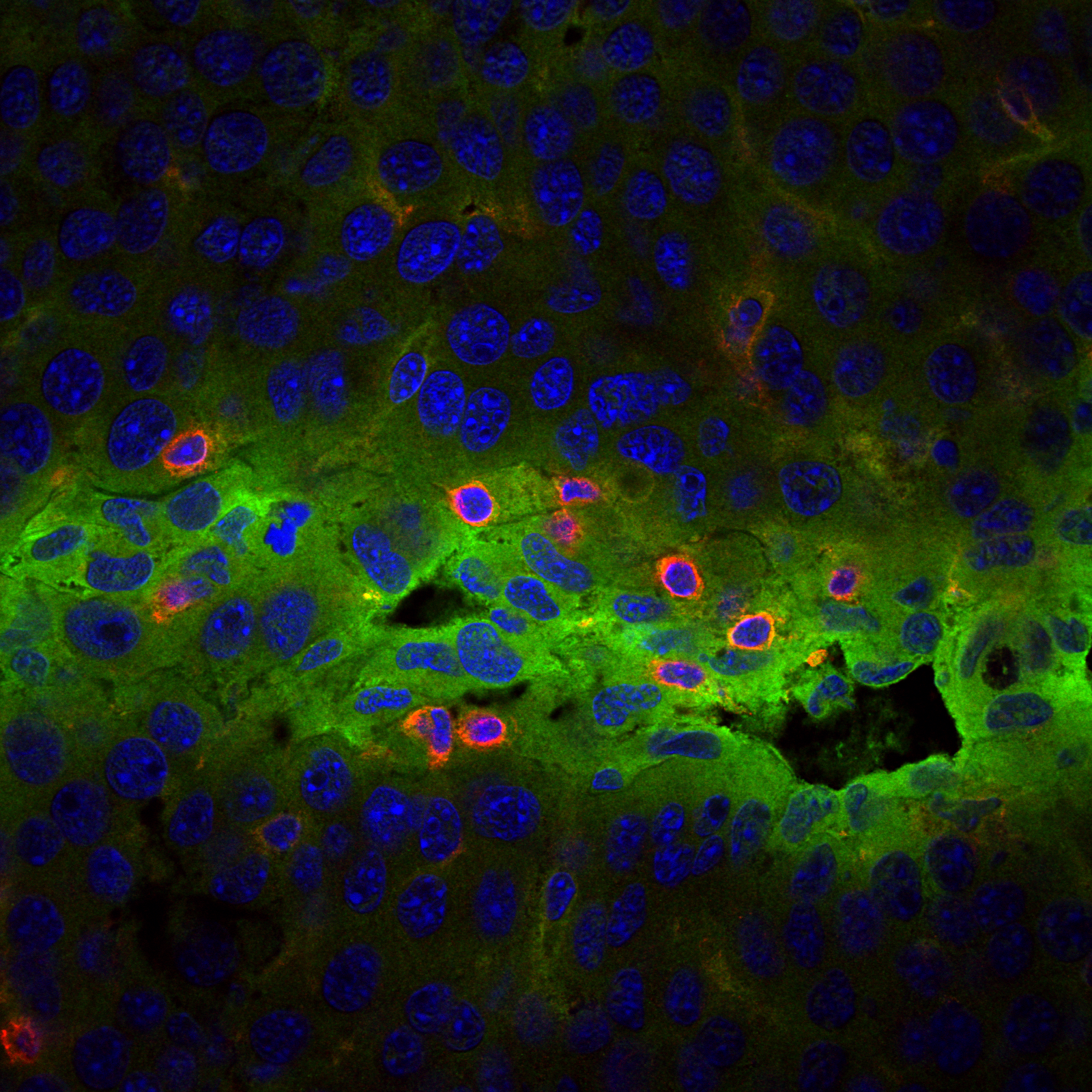

Supplement: Supplementary file 3 — Source Data for Expanded View [file EMMM-12-e11223-s009.zip › EV_source-data/Fig.EV2E/B16 CD45_b_.tif]

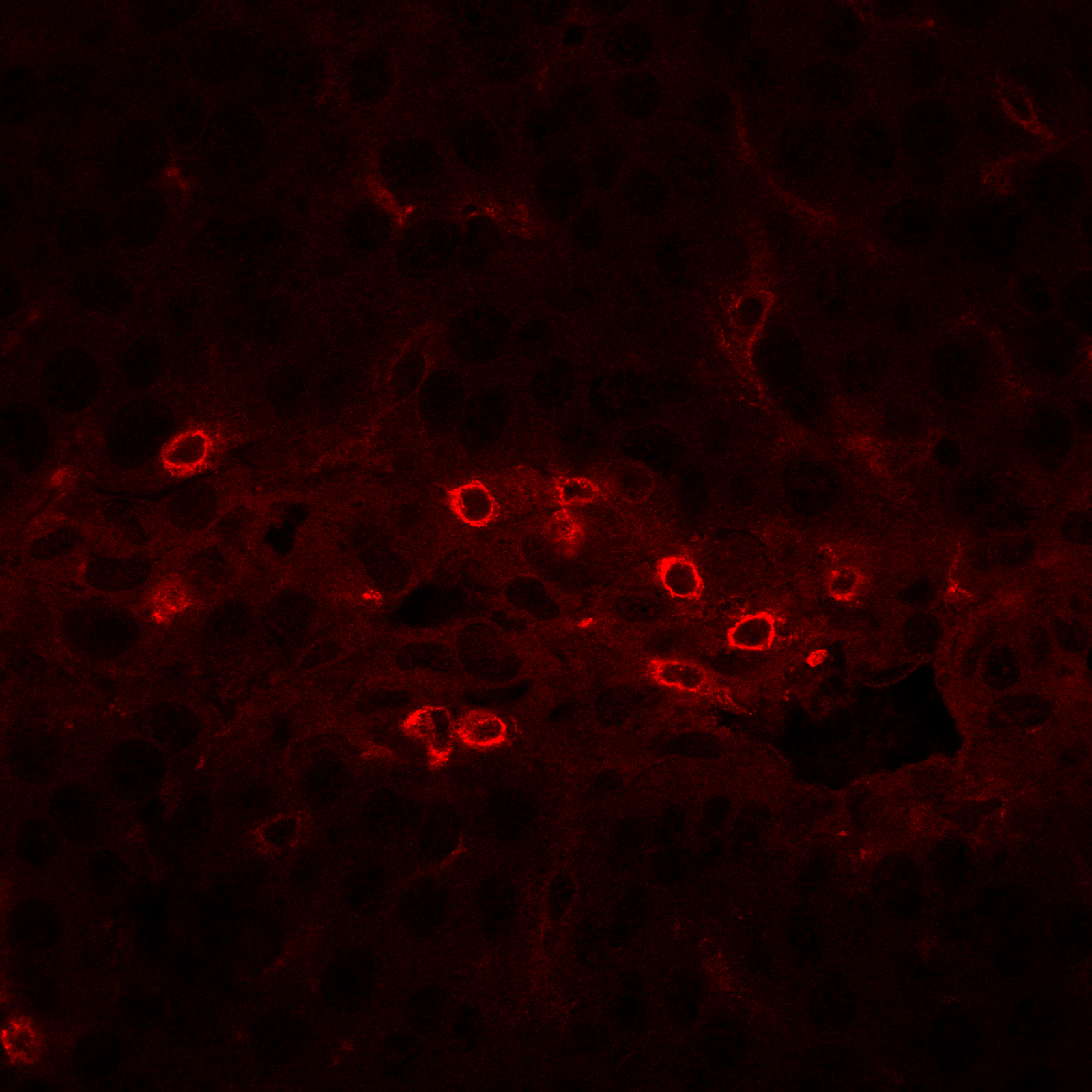

Supplement: Supplementary file 3 — Source Data for Expanded View [file EMMM-12-e11223-s009.zip › EV_source-data/Fig.EV2E/B16 CD45_b_C003.tif]

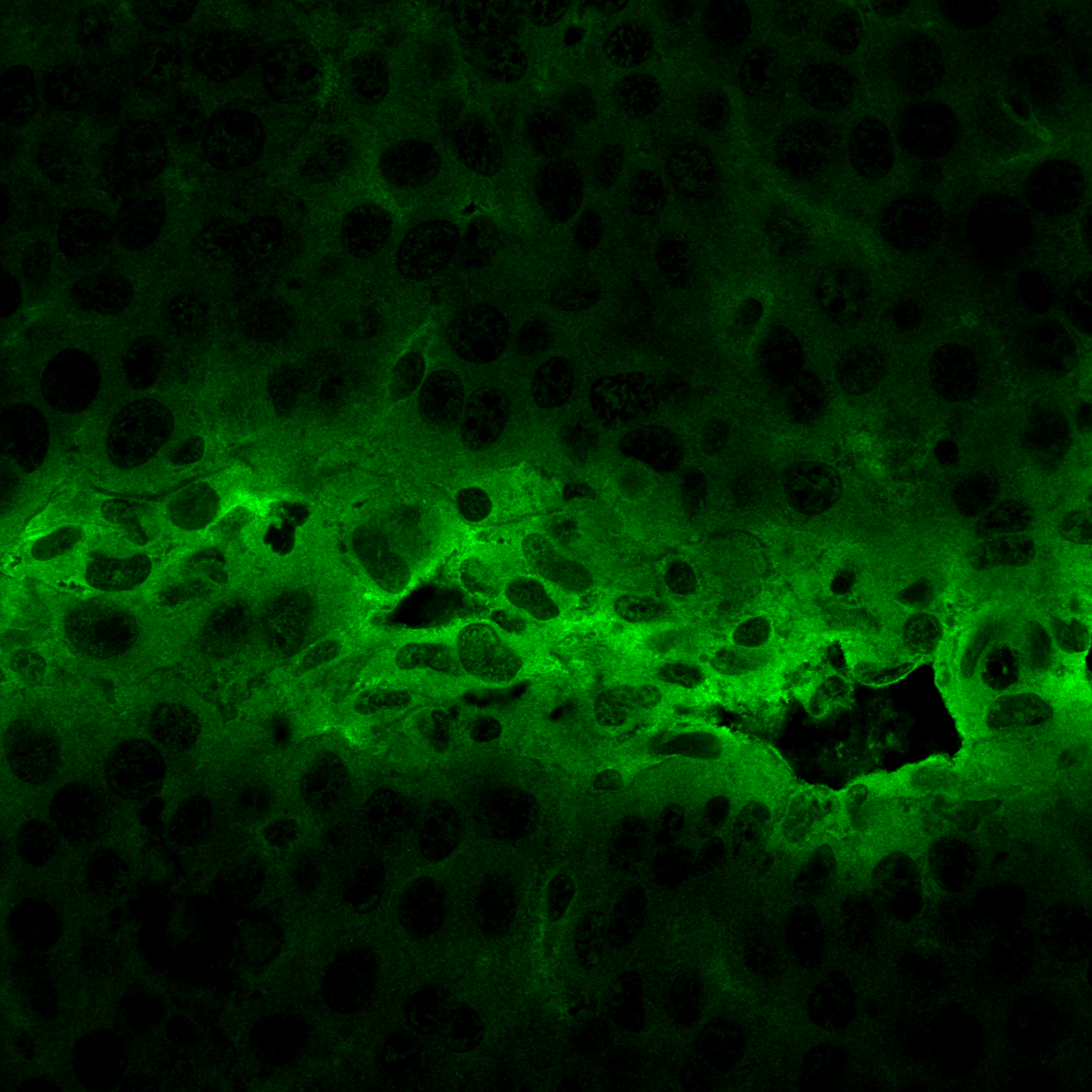

Supplement: Supplementary file 3 — Source Data for Expanded View [file EMMM-12-e11223-s009.zip › EV_source-data/Fig.EV2E/B16 CD45_b_C002.tif]

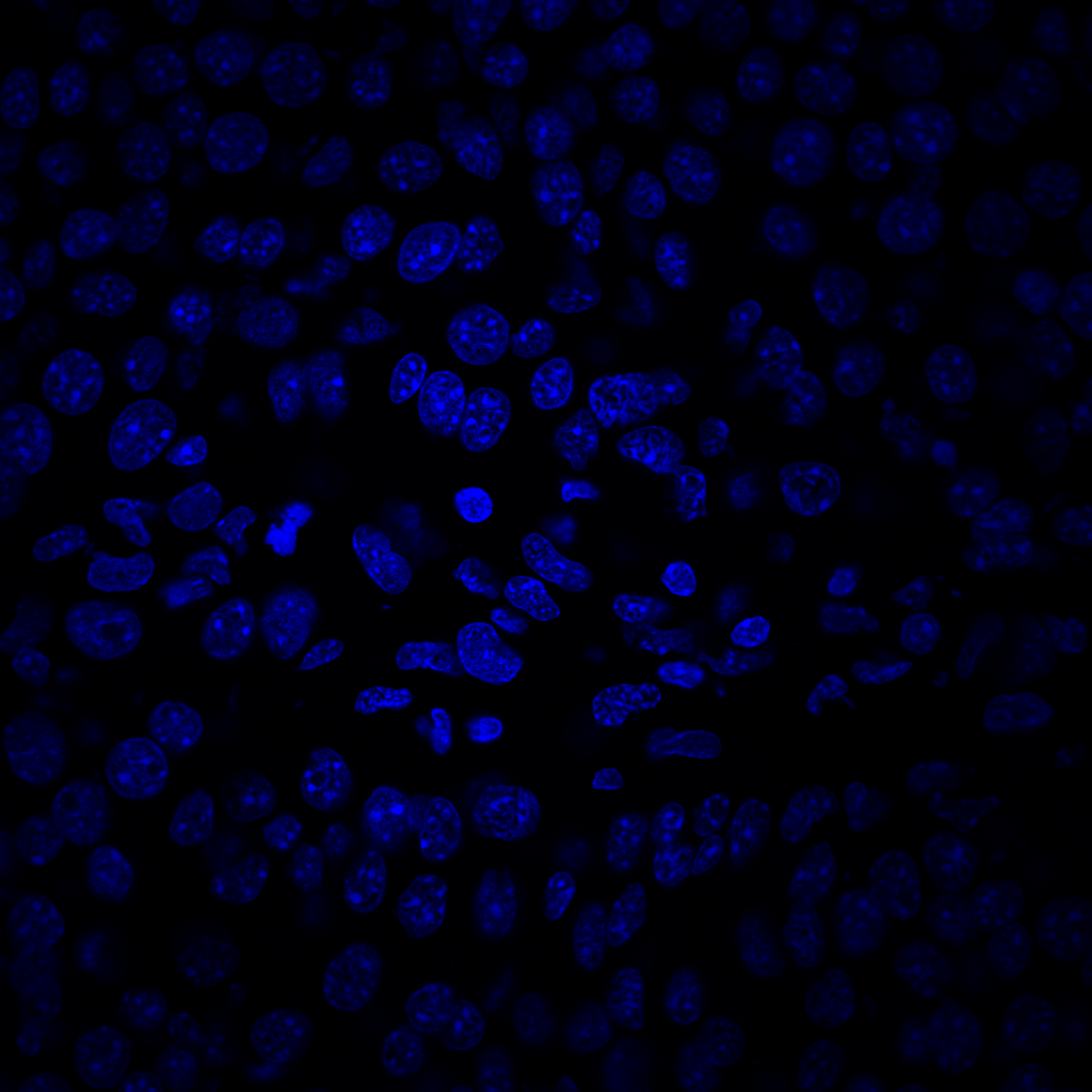

Supplement: Supplementary file 3 — Source Data for Expanded View [file EMMM-12-e11223-s009.zip › EV_source-data/Fig.EV2E/B16 CD45_b_C001.tif]

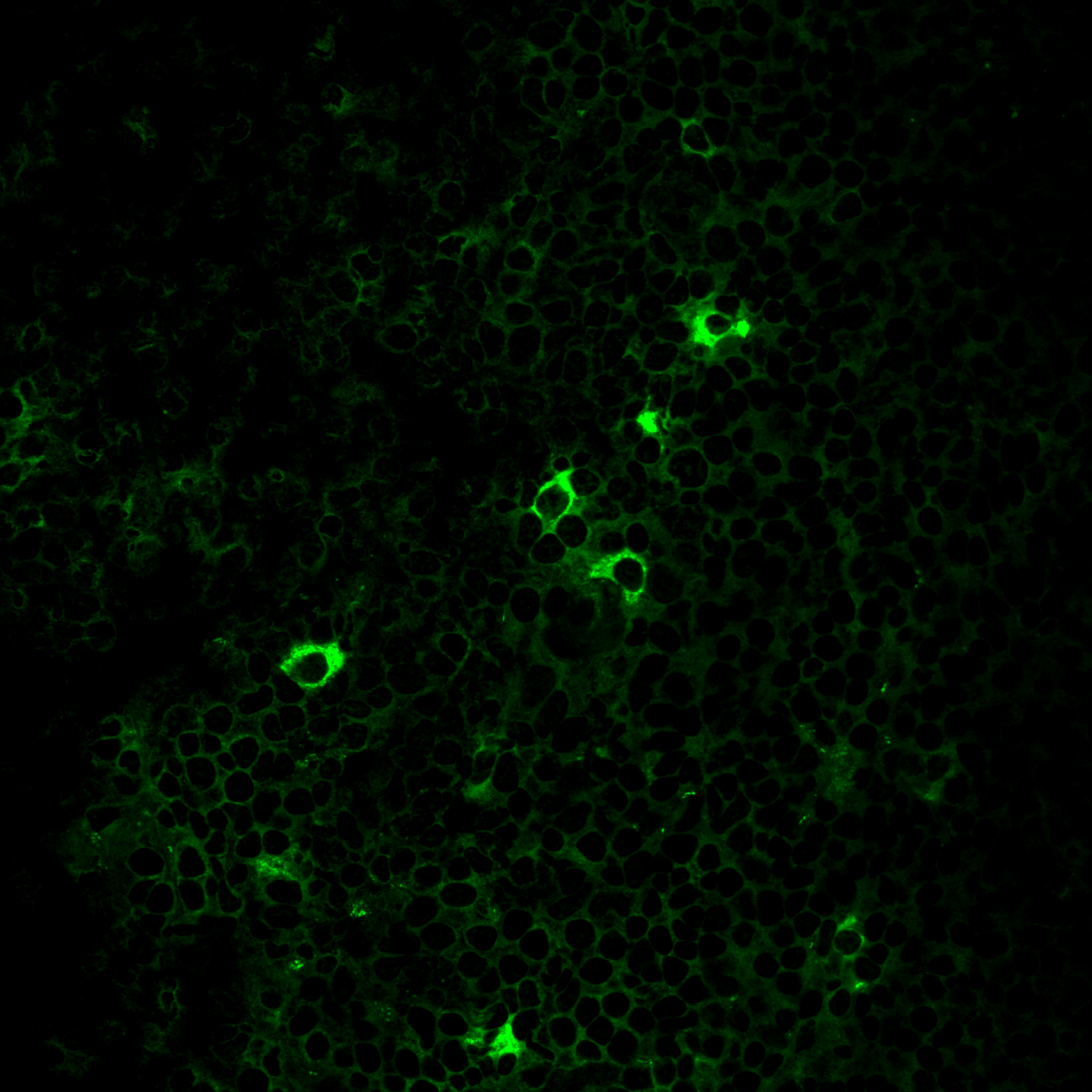

Supplement: Supplementary file 3 — Source Data for Expanded View [file EMMM-12-e11223-s009.zip › EV_source-data/Fig.EV2E/Spleen CD45_C002.tif]

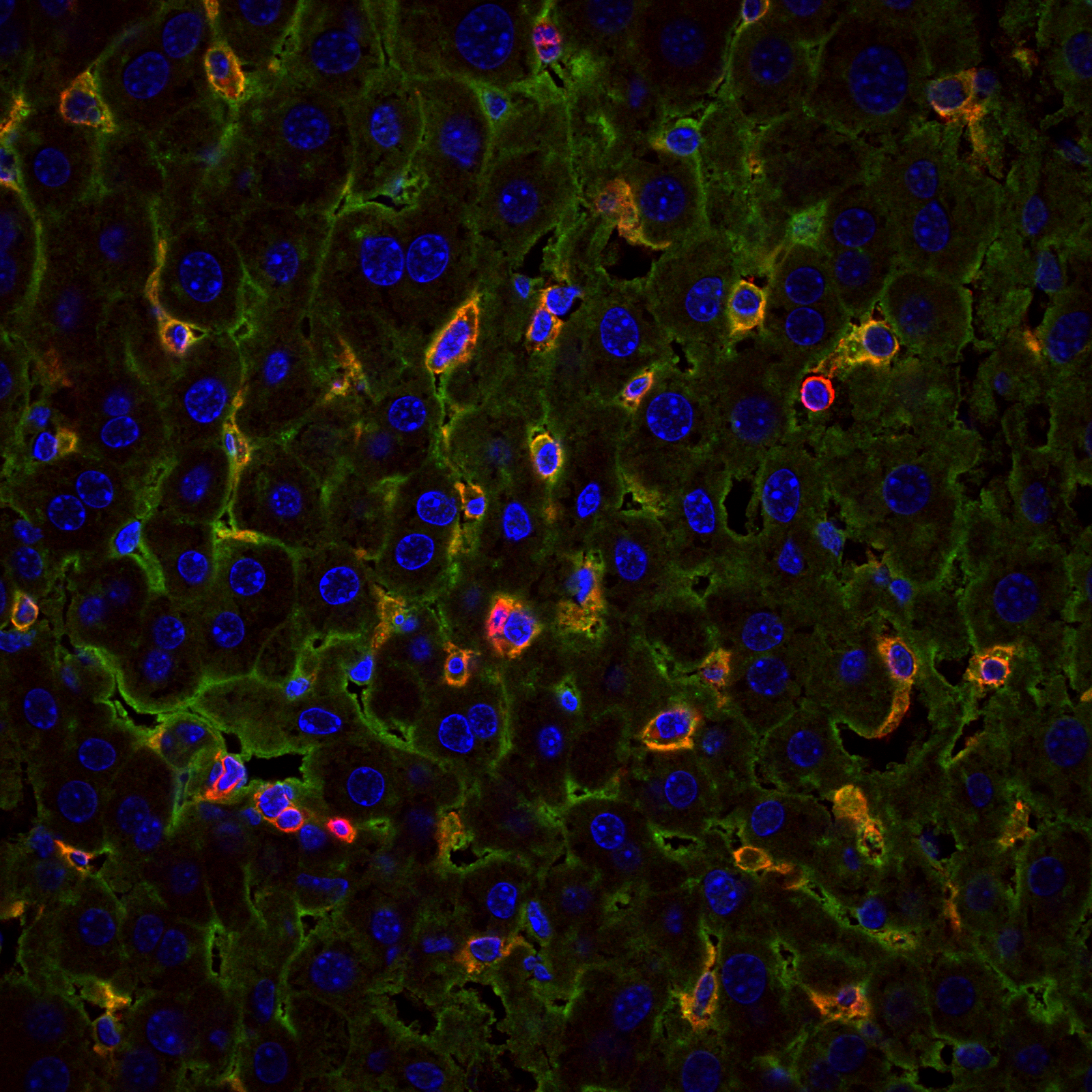

Supplement: Supplementary file 3 — Source Data for Expanded View [file EMMM-12-e11223-s009.zip › EV_source-data/Fig.EV2E/Liver CD45_.tif]

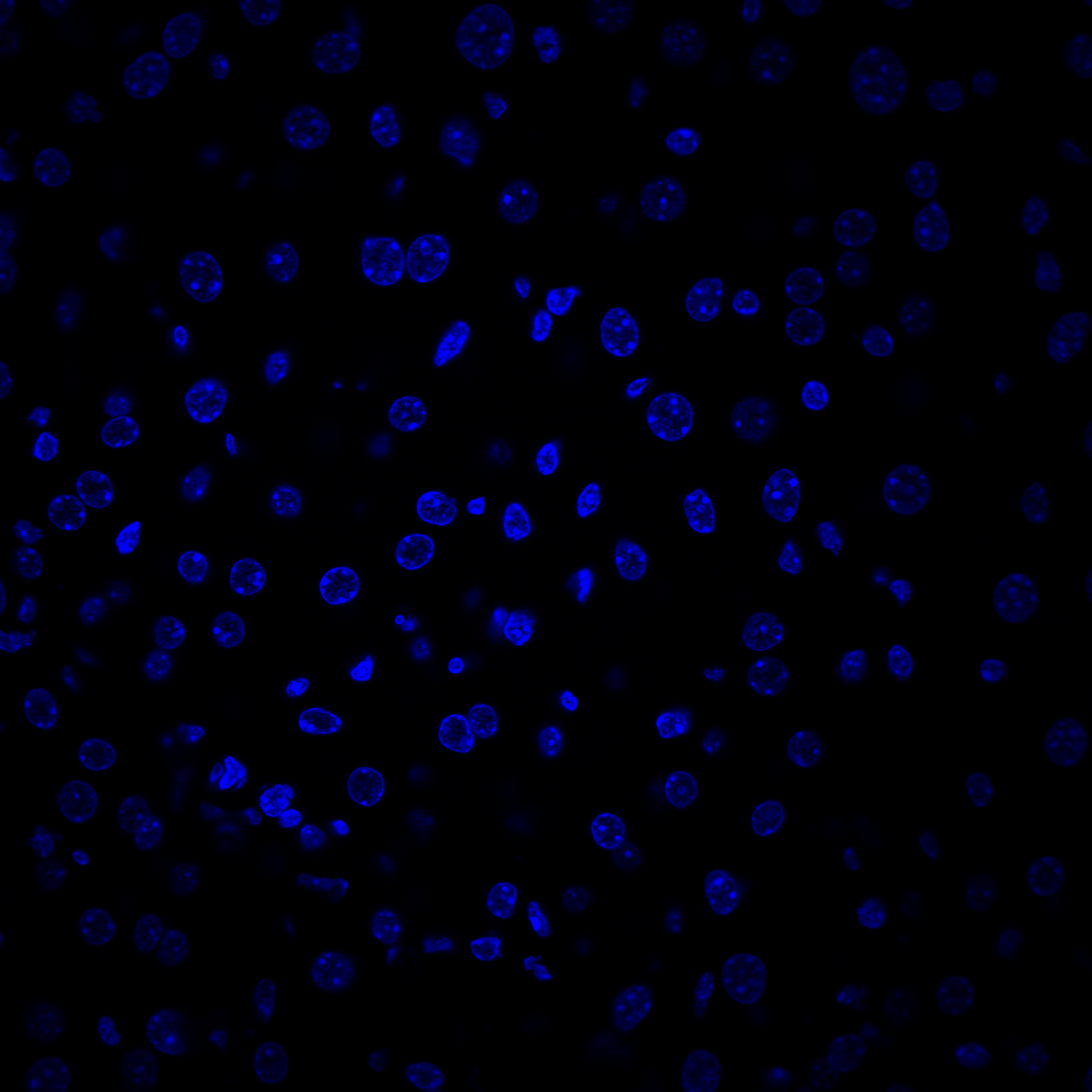

Supplement: Supplementary file 3 — Source Data for Expanded View [file EMMM-12-e11223-s009.zip › EV_source-data/Fig.EV2E/Liver CD45_C001.tif]

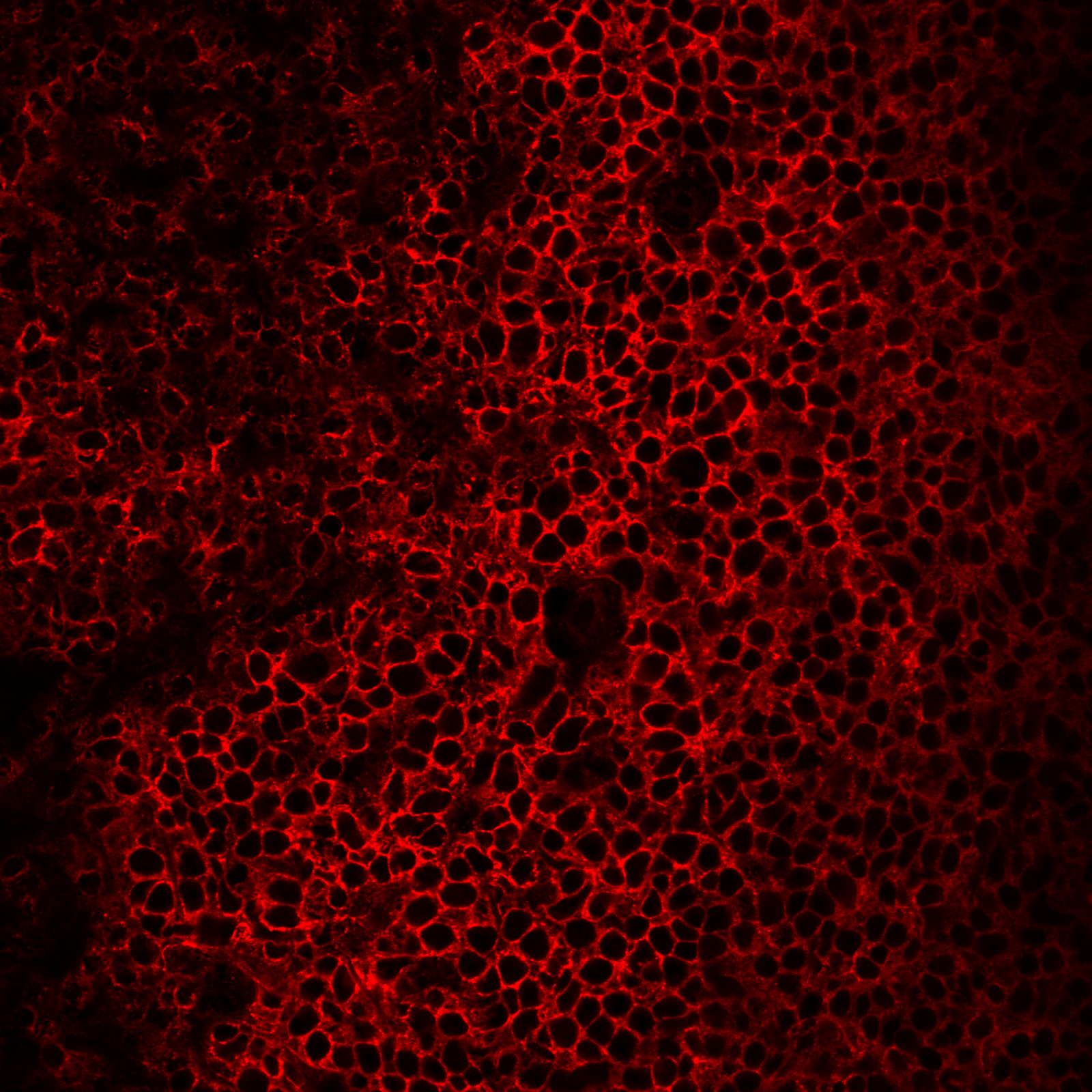

Supplement: Supplementary file 3 — Source Data for Expanded View [file EMMM-12-e11223-s009.zip › EV_source-data/Fig.EV2E/Spleen CD45_C003.tif]

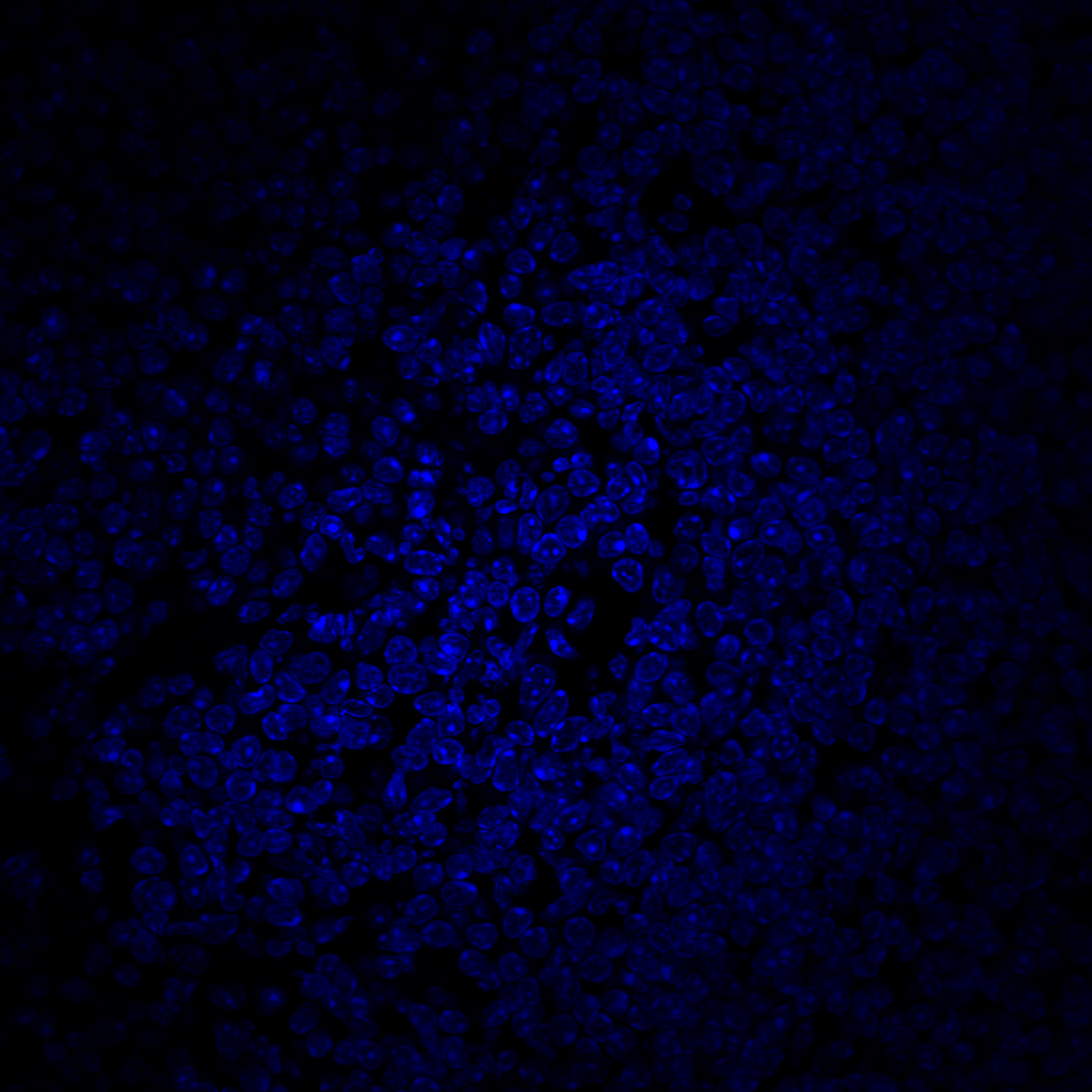

Supplement: Supplementary file 3 — Source Data for Expanded View [file EMMM-12-e11223-s009.zip › EV_source-data/Fig.EV2E/Spleen CD45_C001.tif]

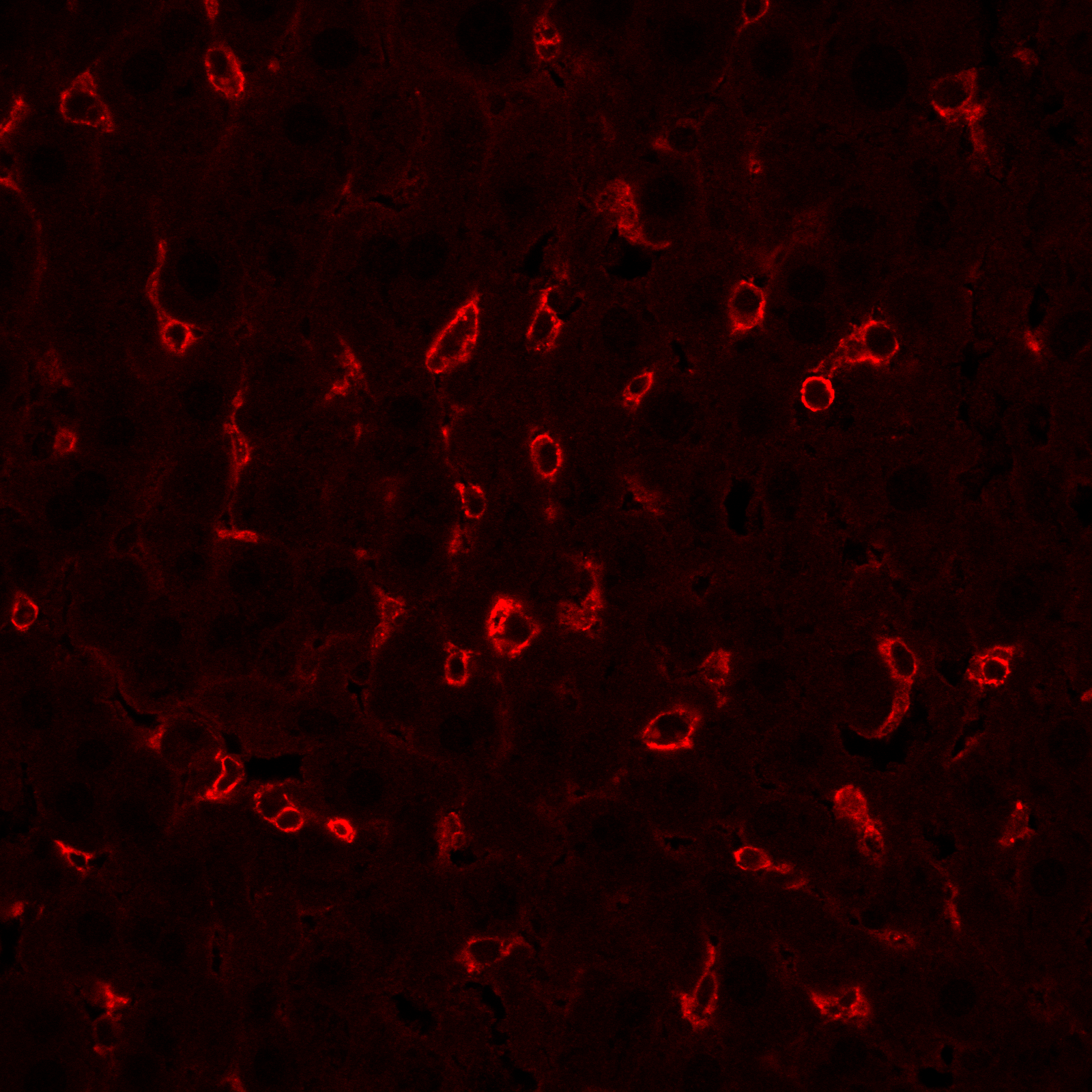

Supplement: Supplementary file 3 — Source Data for Expanded View [file EMMM-12-e11223-s009.zip › EV_source-data/Fig.EV2E/Liver CD45_C003.tif]

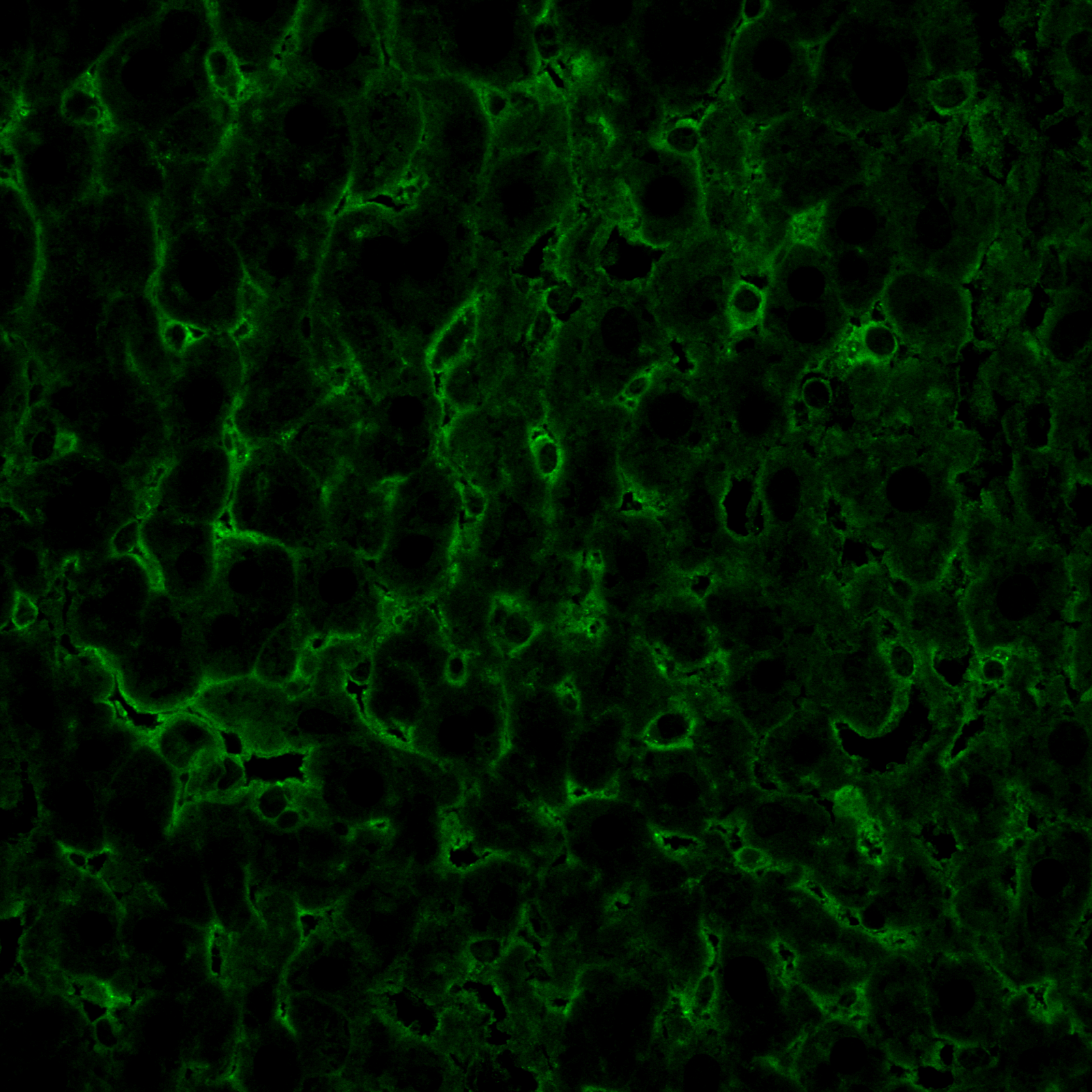

Supplement: Supplementary file 3 — Source Data for Expanded View [file EMMM-12-e11223-s009.zip › EV_source-data/Fig.EV2E/Liver CD45_C002.tif]

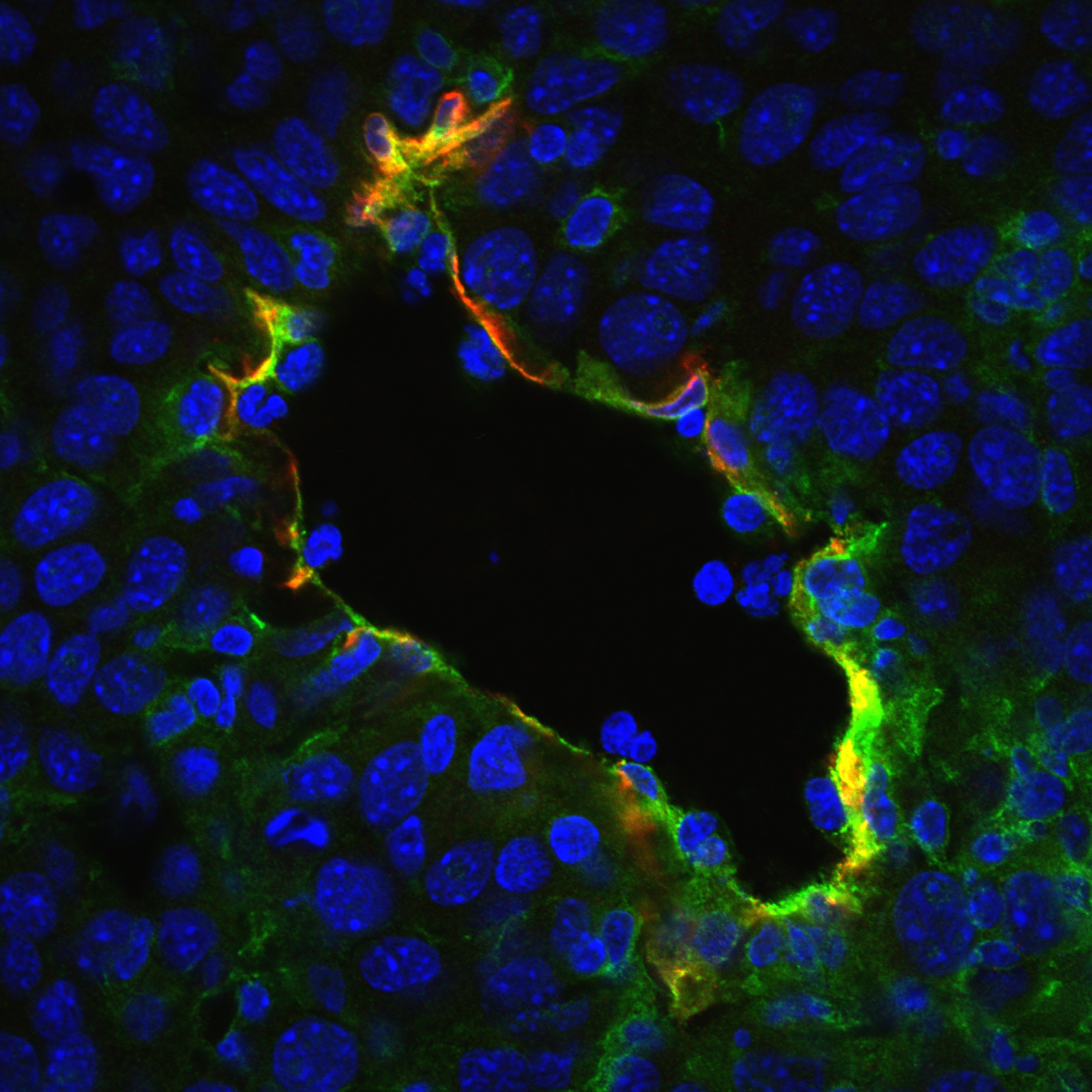

Supplement: Supplementary file 7 — Source Data for Figure 3 [file EMMM-12-e11223-s005.zip › Fig3/Fig.3C/AFR_6u_2_.tif]

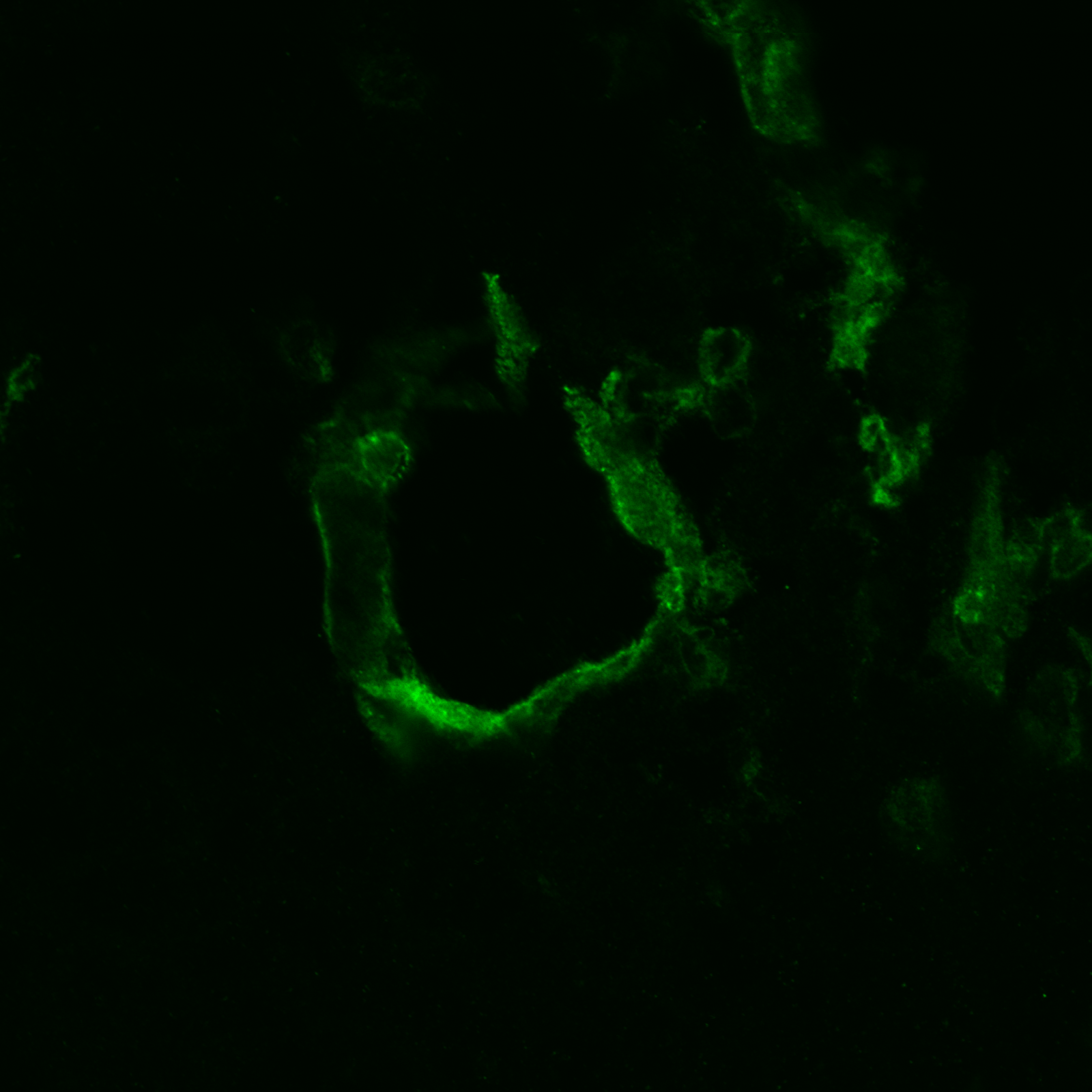

Supplement: Supplementary file 7 — Source Data for Figure 3 [file EMMM-12-e11223-s005.zip › Fig3/Fig.3C/mTNF_6u_2_C002.tif]

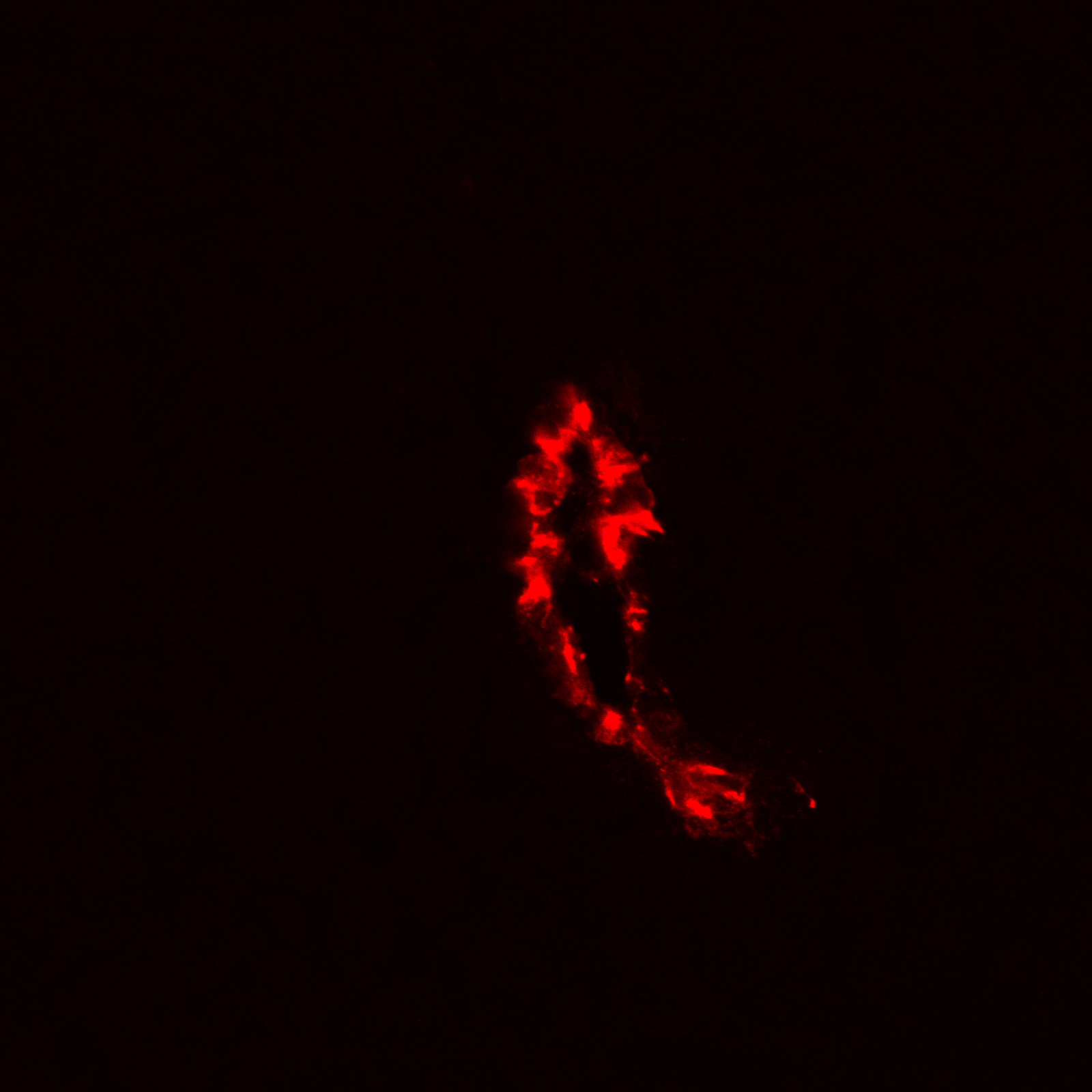

Supplement: Supplementary file 7 — Source Data for Figure 3 [file EMMM-12-e11223-s005.zip › Fig3/Fig.3C/AFR_24u_2_C003.tif]

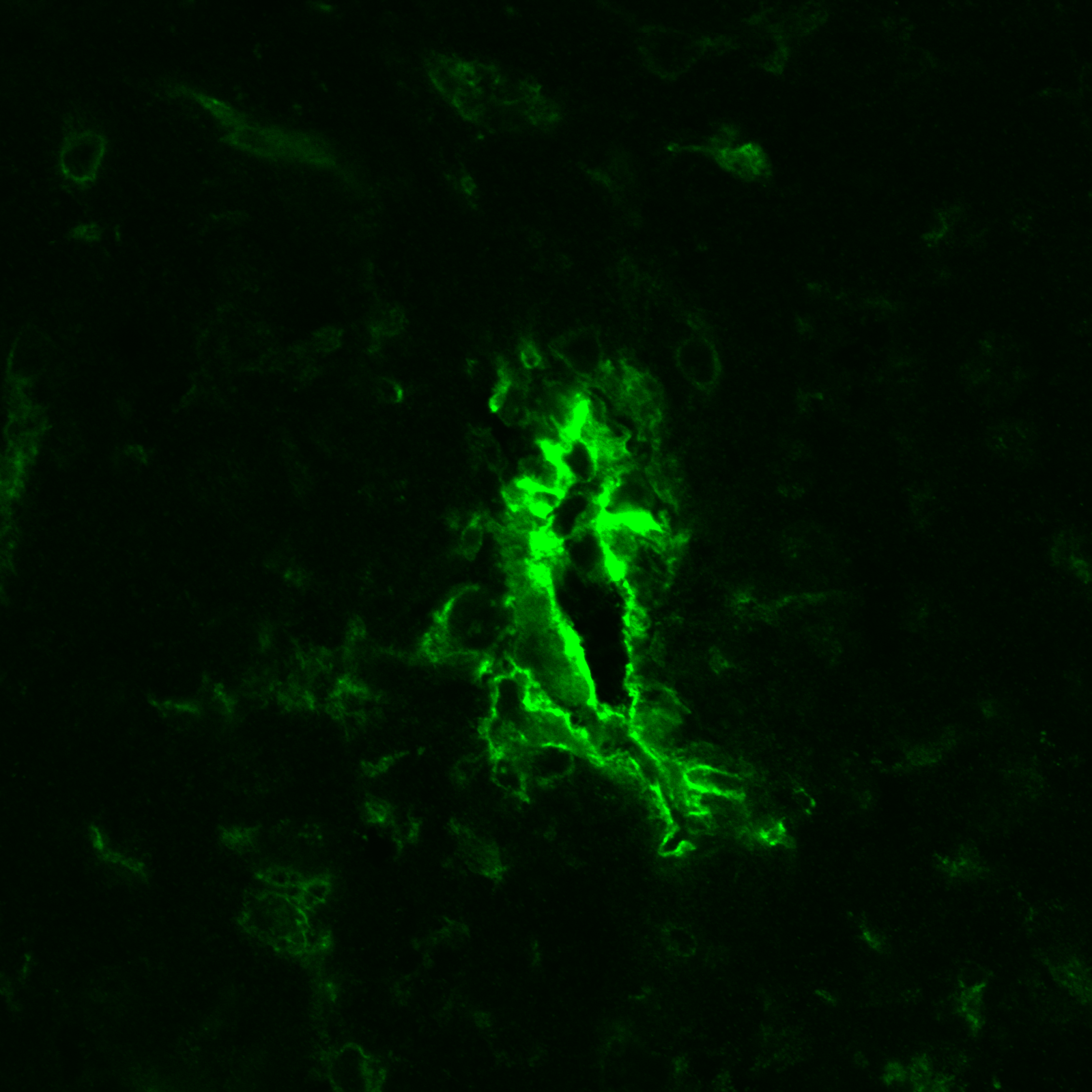

Supplement: Supplementary file 7 — Source Data for Figure 3 [file EMMM-12-e11223-s005.zip › Fig3/Fig.3C/AFR_24u_2_C002.tif]

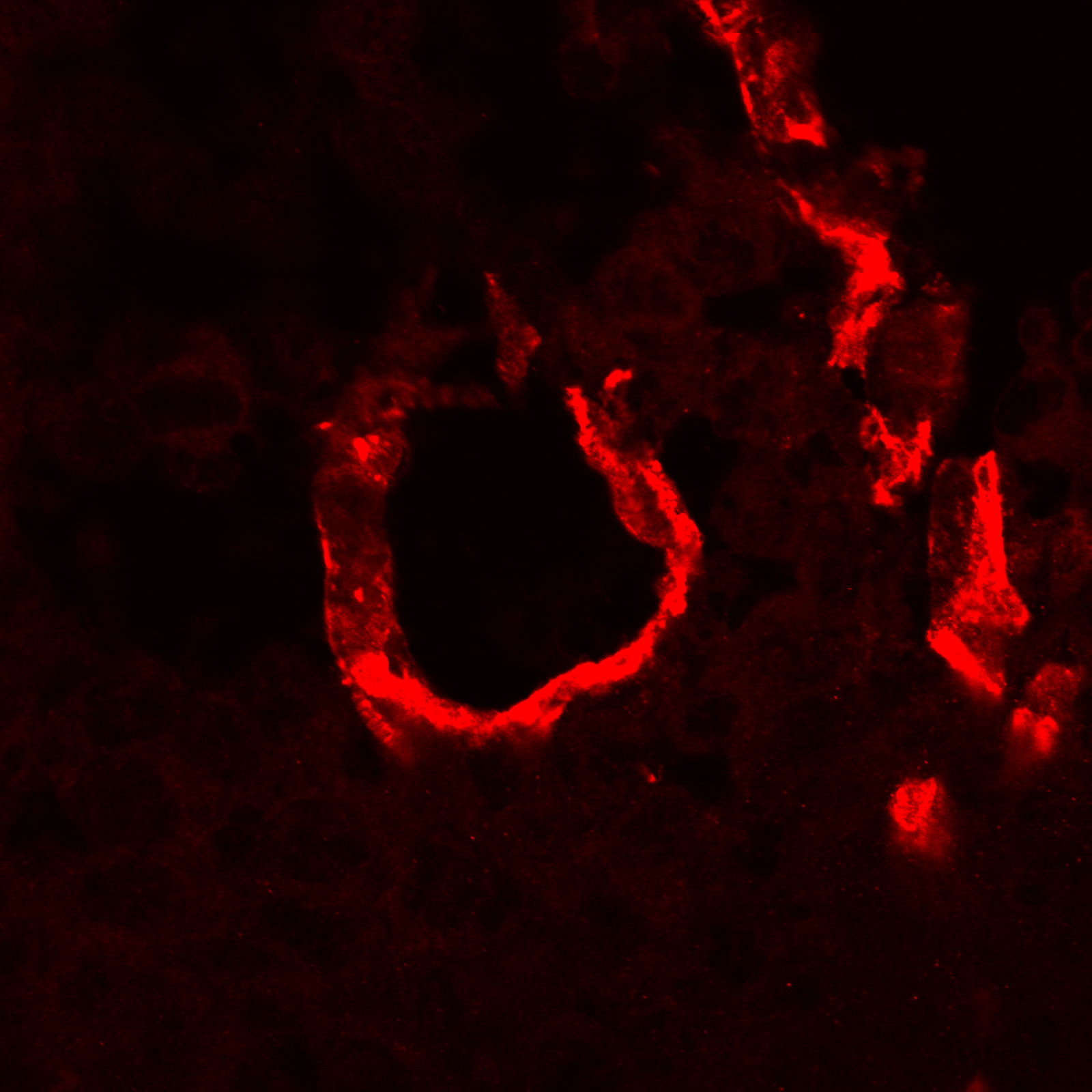

Supplement: Supplementary file 7 — Source Data for Figure 3 [file EMMM-12-e11223-s005.zip › Fig3/Fig.3C/mTNF_6u_2_C003.tif]

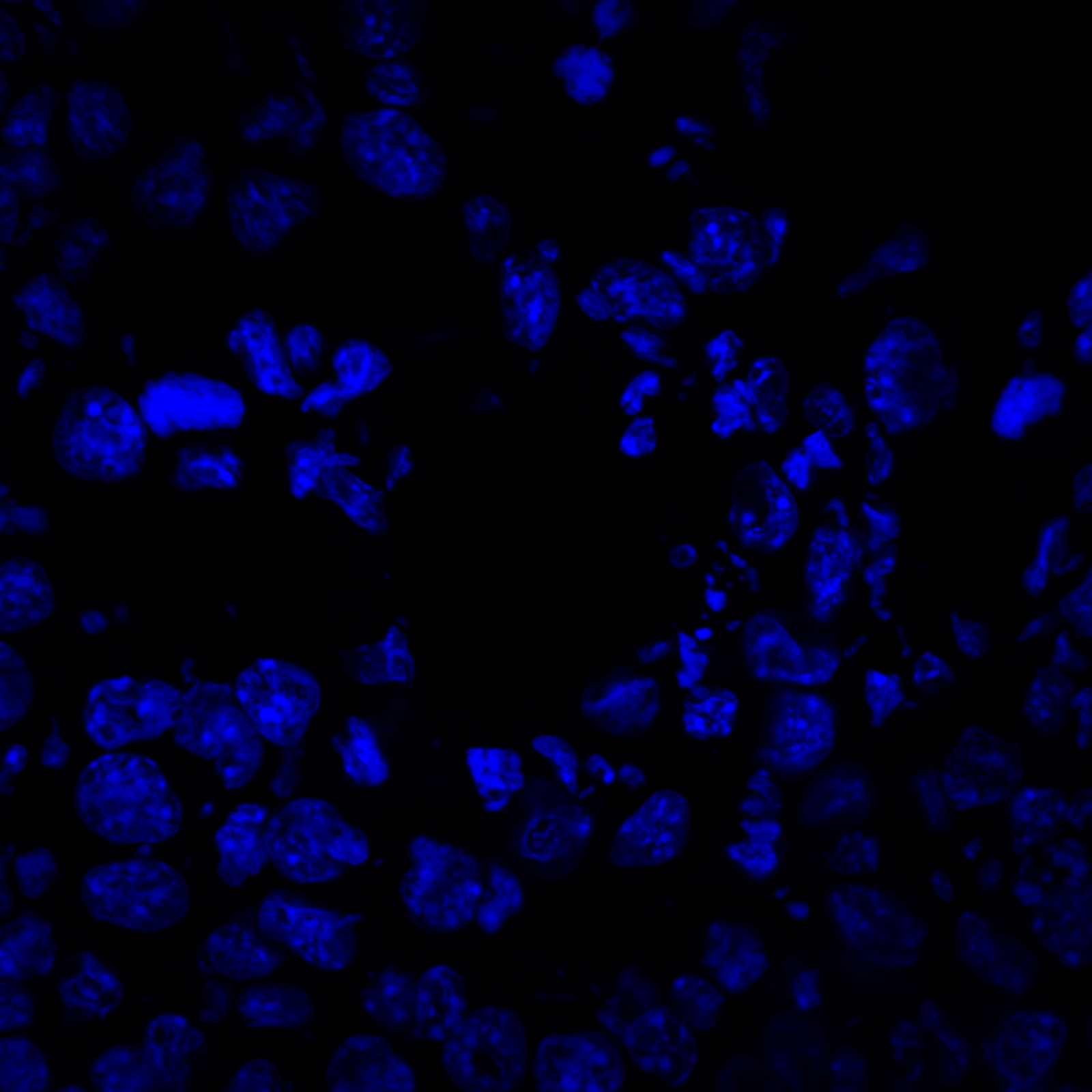

Supplement: Supplementary file 7 — Source Data for Figure 3 [file EMMM-12-e11223-s005.zip › Fig3/Fig.3C/mTNF_6u_2_C001.tif]

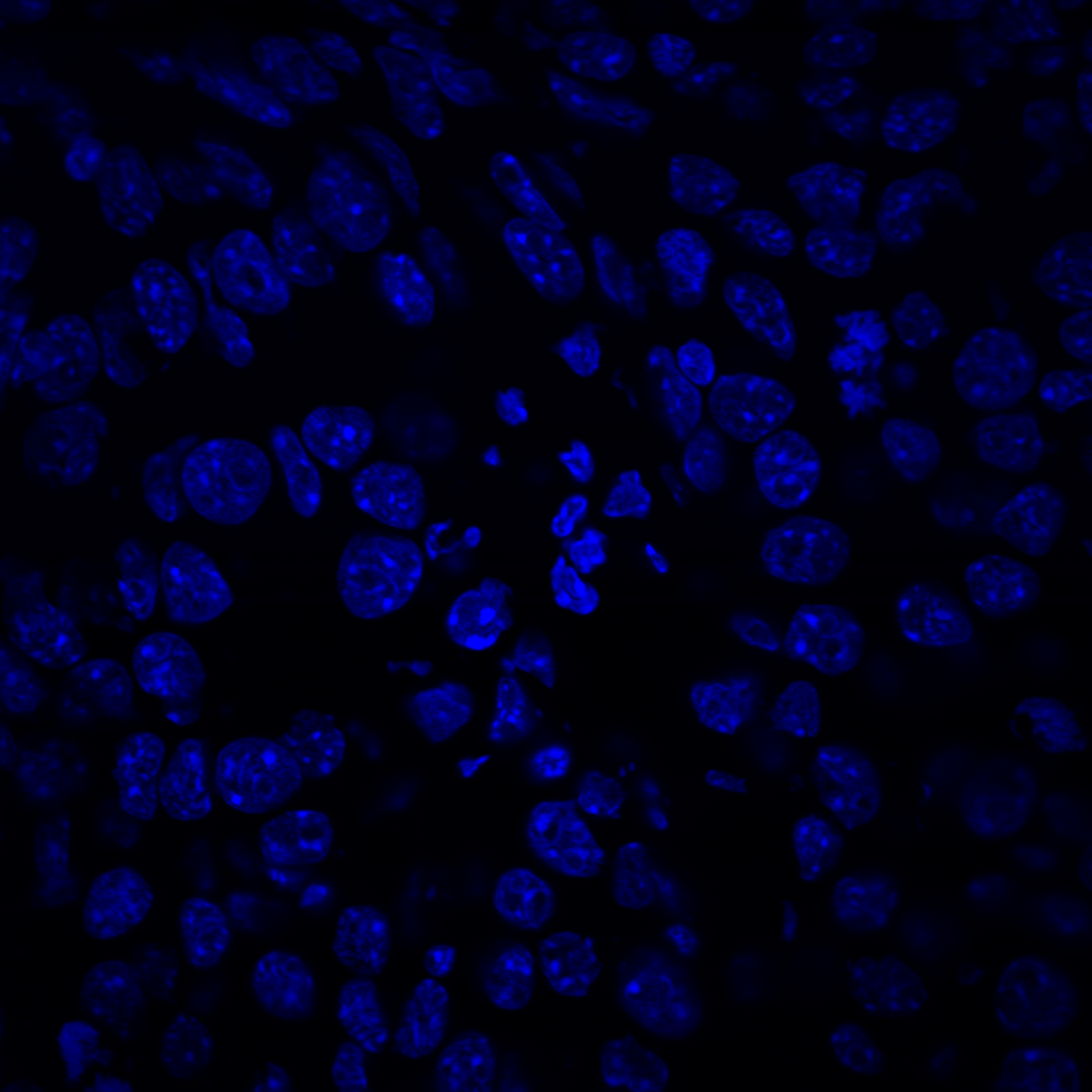

Supplement: Supplementary file 7 — Source Data for Figure 3 [file EMMM-12-e11223-s005.zip › Fig3/Fig.3C/AFR_24u_2_C001.tif]

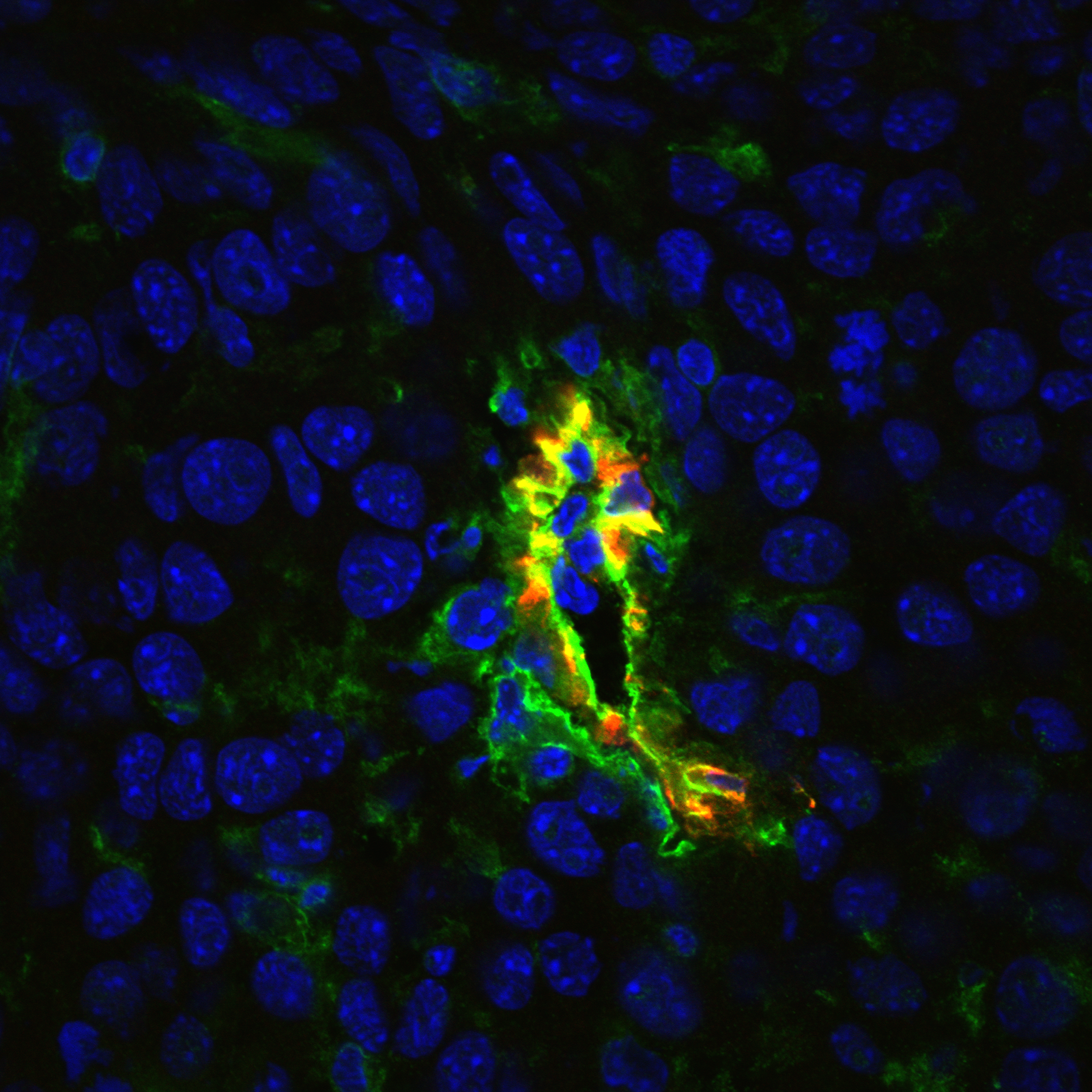

Supplement: Supplementary file 7 — Source Data for Figure 3 [file EMMM-12-e11223-s005.zip › Fig3/Fig.3C/AFR_24u_2_.tif]

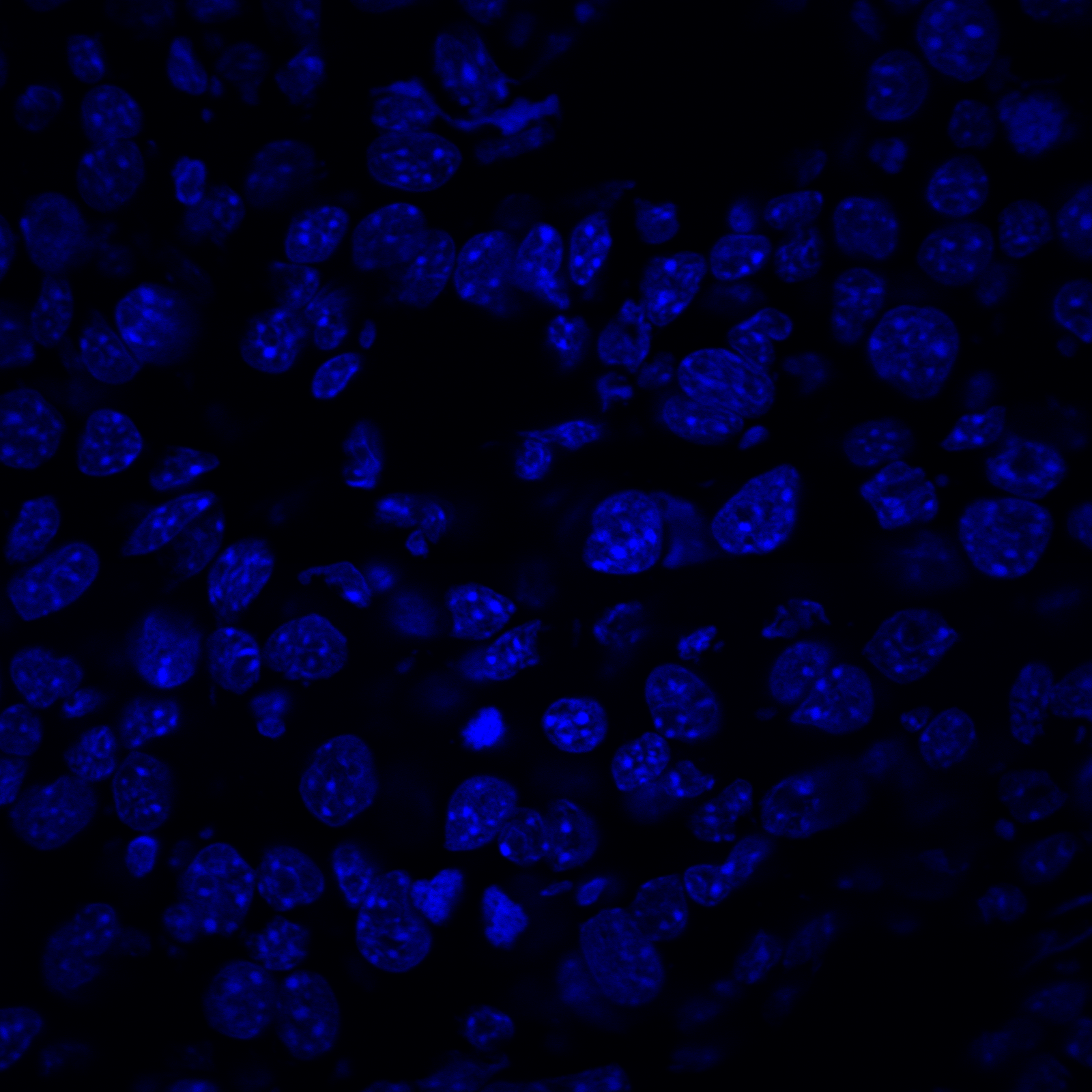

Supplement: Supplementary file 7 — Source Data for Figure 3 [file EMMM-12-e11223-s005.zip › Fig3/Fig.3C/PBS_2_C001.tif]

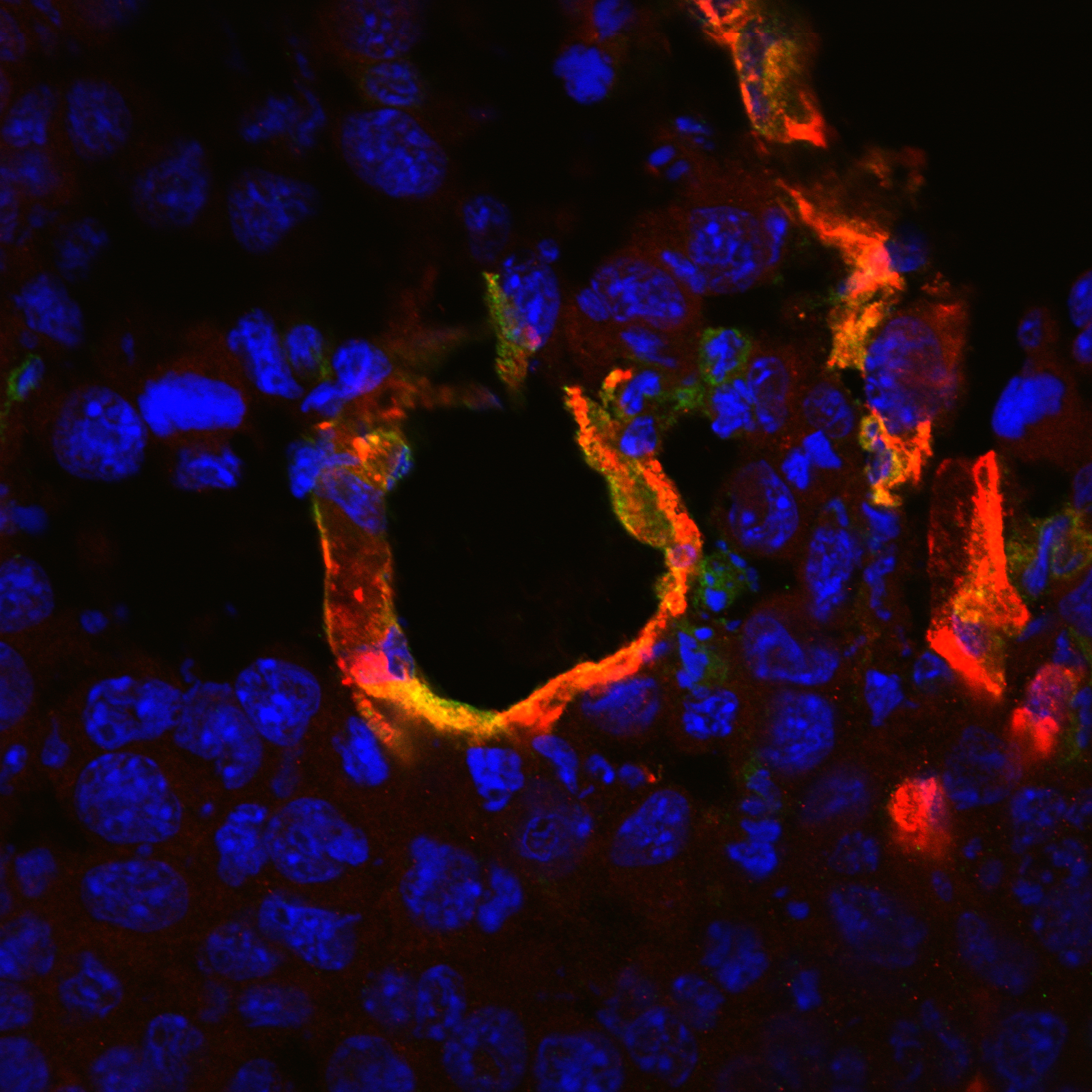

Supplement: Supplementary file 7 — Source Data for Figure 3 [file EMMM-12-e11223-s005.zip › Fig3/Fig.3C/mTNF_6u_2_.tif]

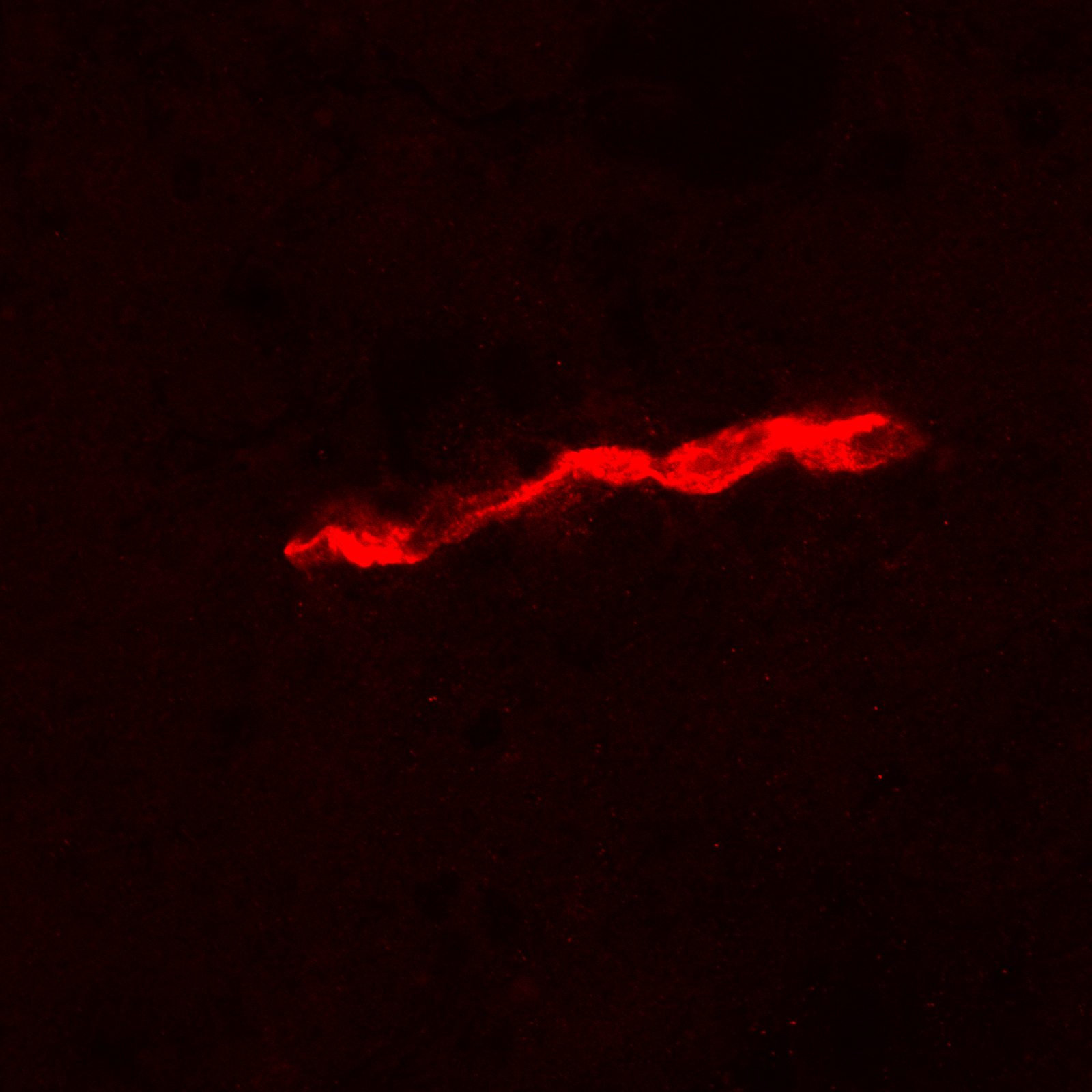

Supplement: Supplementary file 7 — Source Data for Figure 3 [file EMMM-12-e11223-s005.zip › Fig3/Fig.3C/PBS_2_C003.tif]

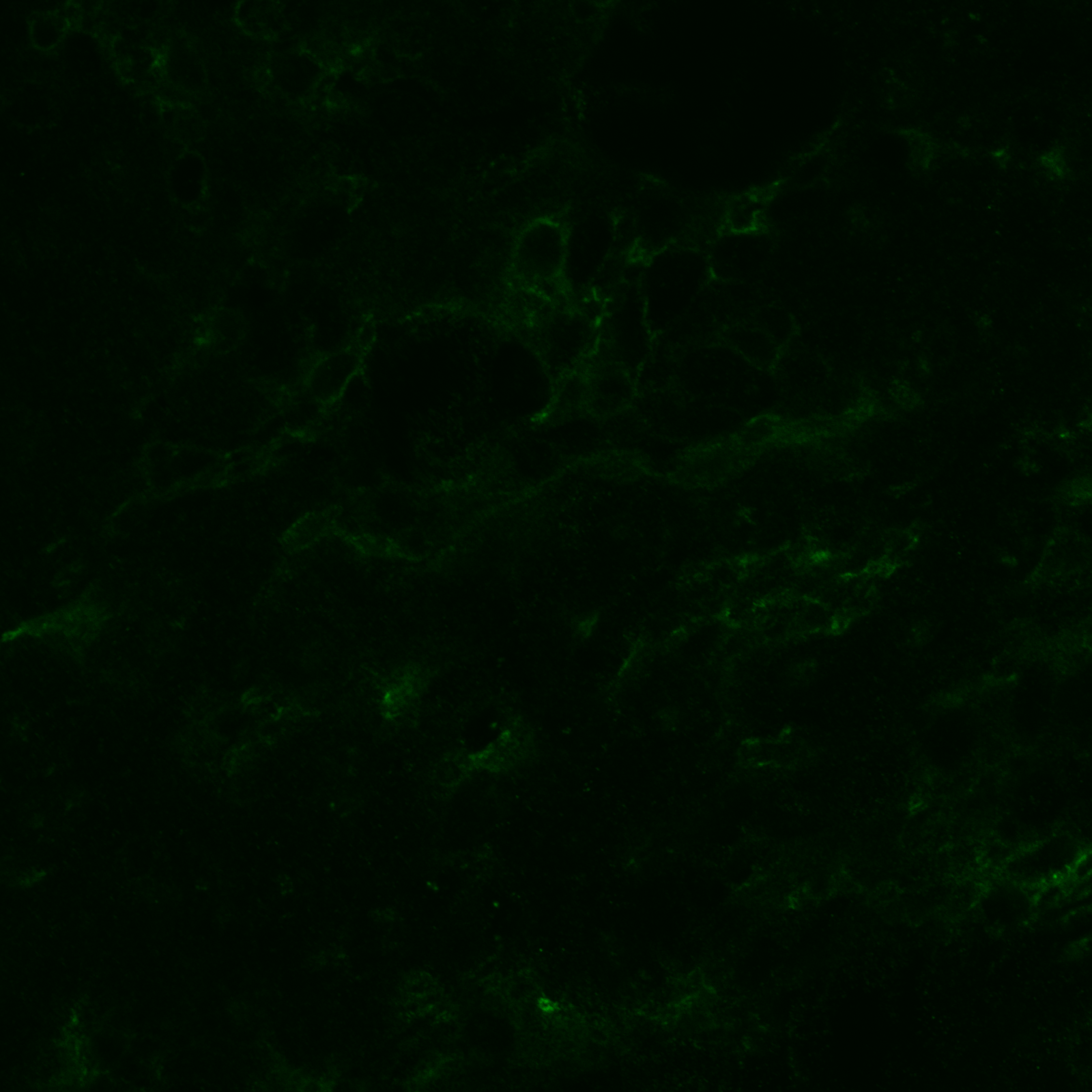

Supplement: Supplementary file 7 — Source Data for Figure 3 [file EMMM-12-e11223-s005.zip › Fig3/Fig.3C/PBS_2_C002.tif]

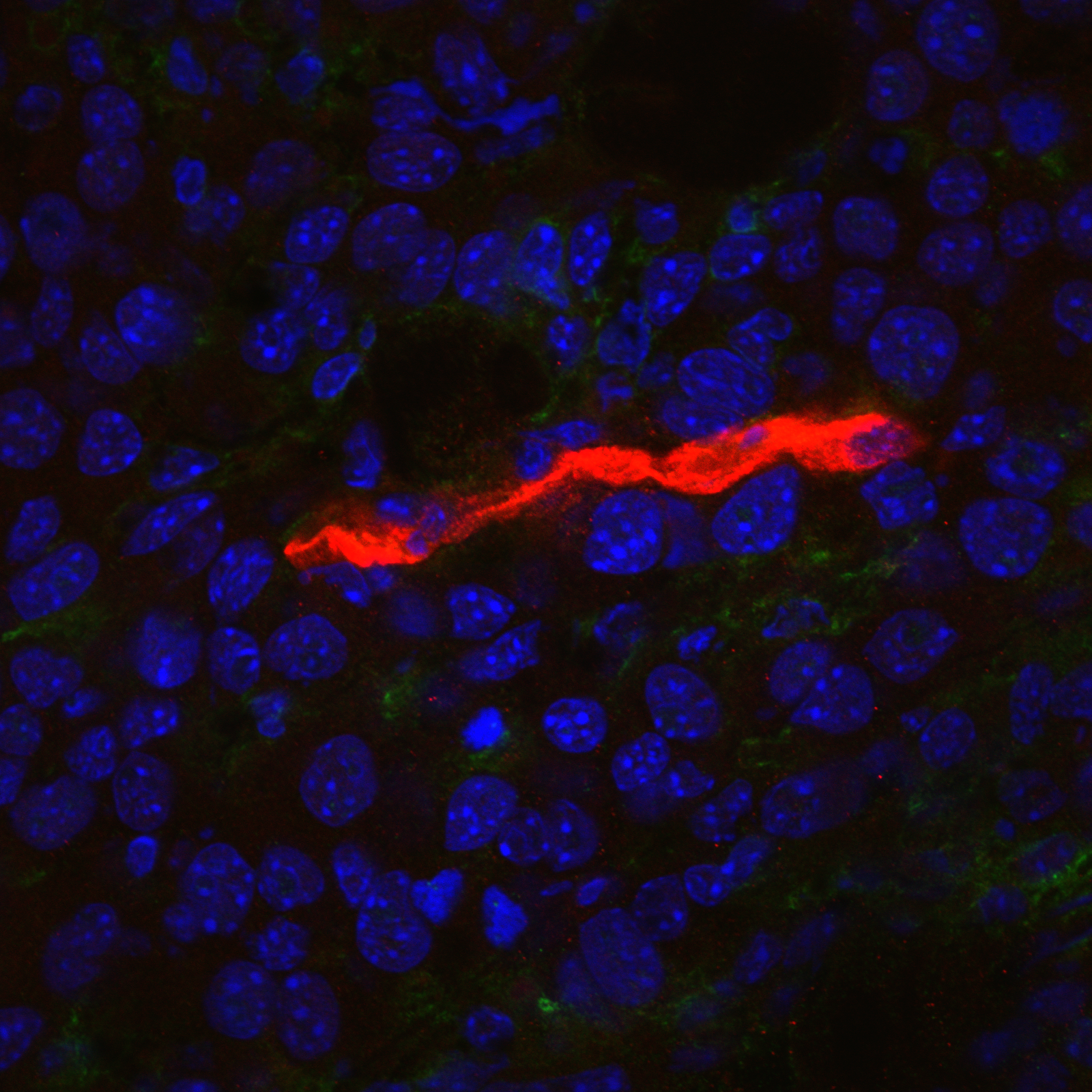

Supplement: Supplementary file 7 — Source Data for Figure 3 [file EMMM-12-e11223-s005.zip › Fig3/Fig.3C/PBS_2_.tif]

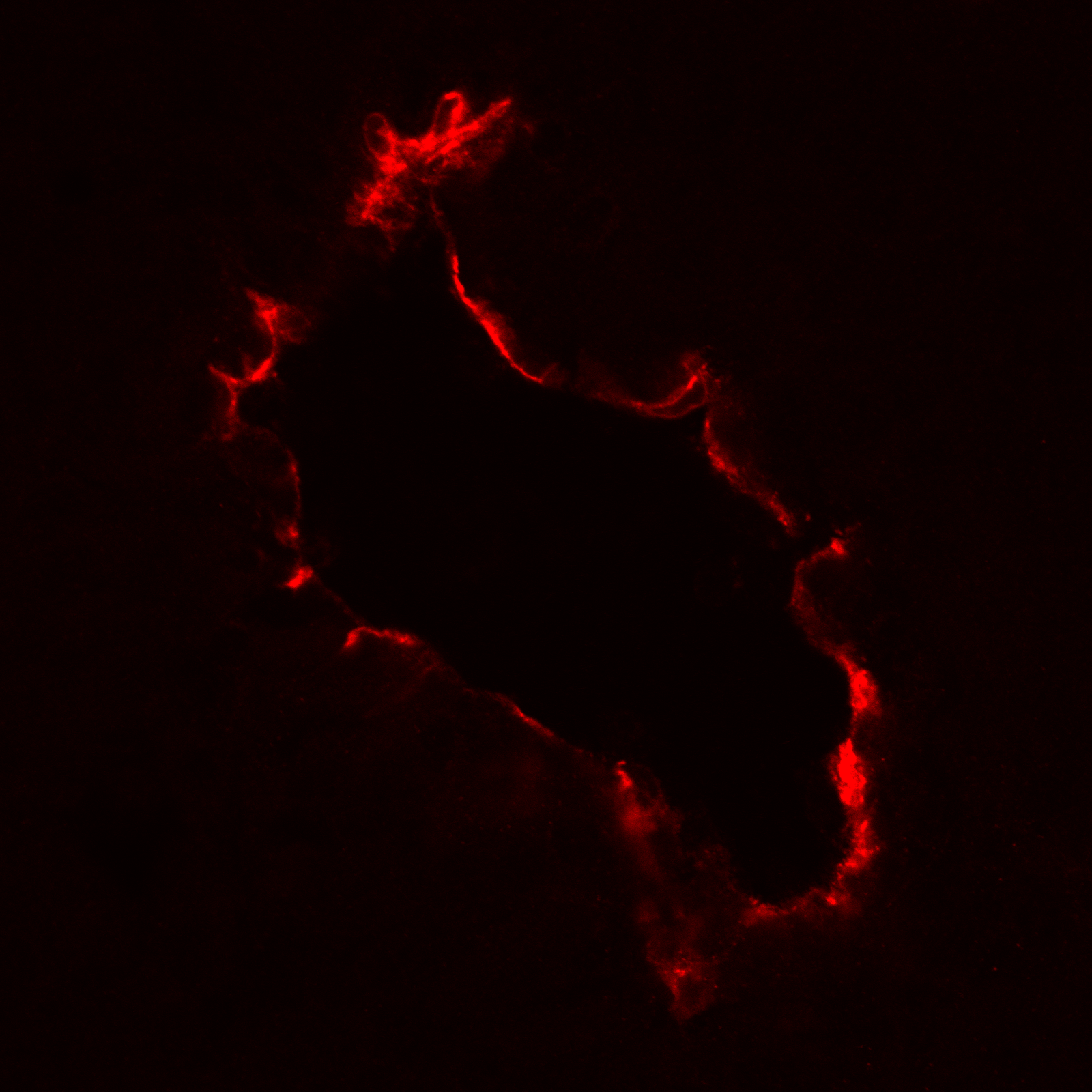

Supplement: Supplementary file 7 — Source Data for Figure 3 [file EMMM-12-e11223-s005.zip › Fig3/Fig.3C/AFR_6u_2_C003.tif]

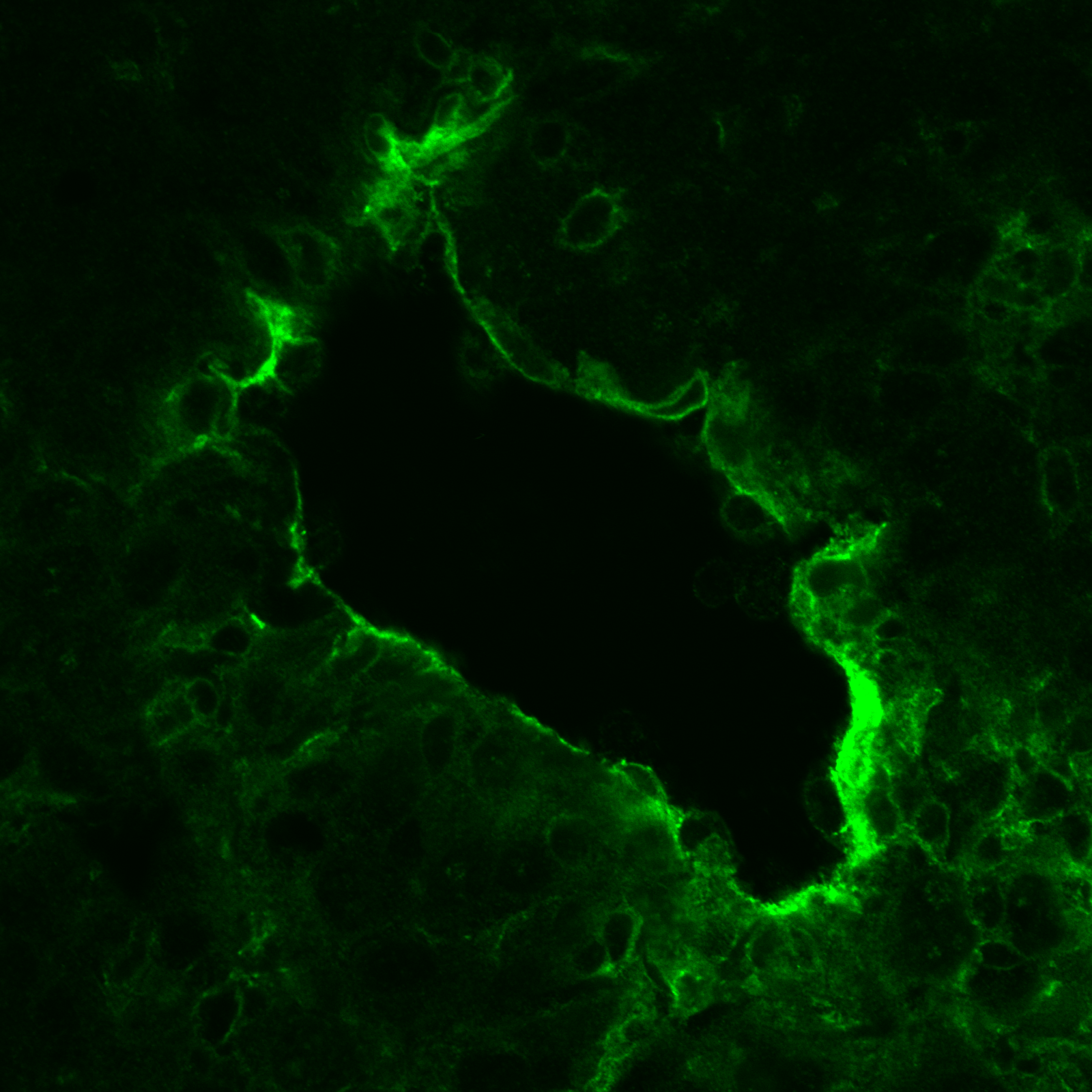

Supplement: Supplementary file 7 — Source Data for Figure 3 [file EMMM-12-e11223-s005.zip › Fig3/Fig.3C/AFR_6u_2_C002.tif]

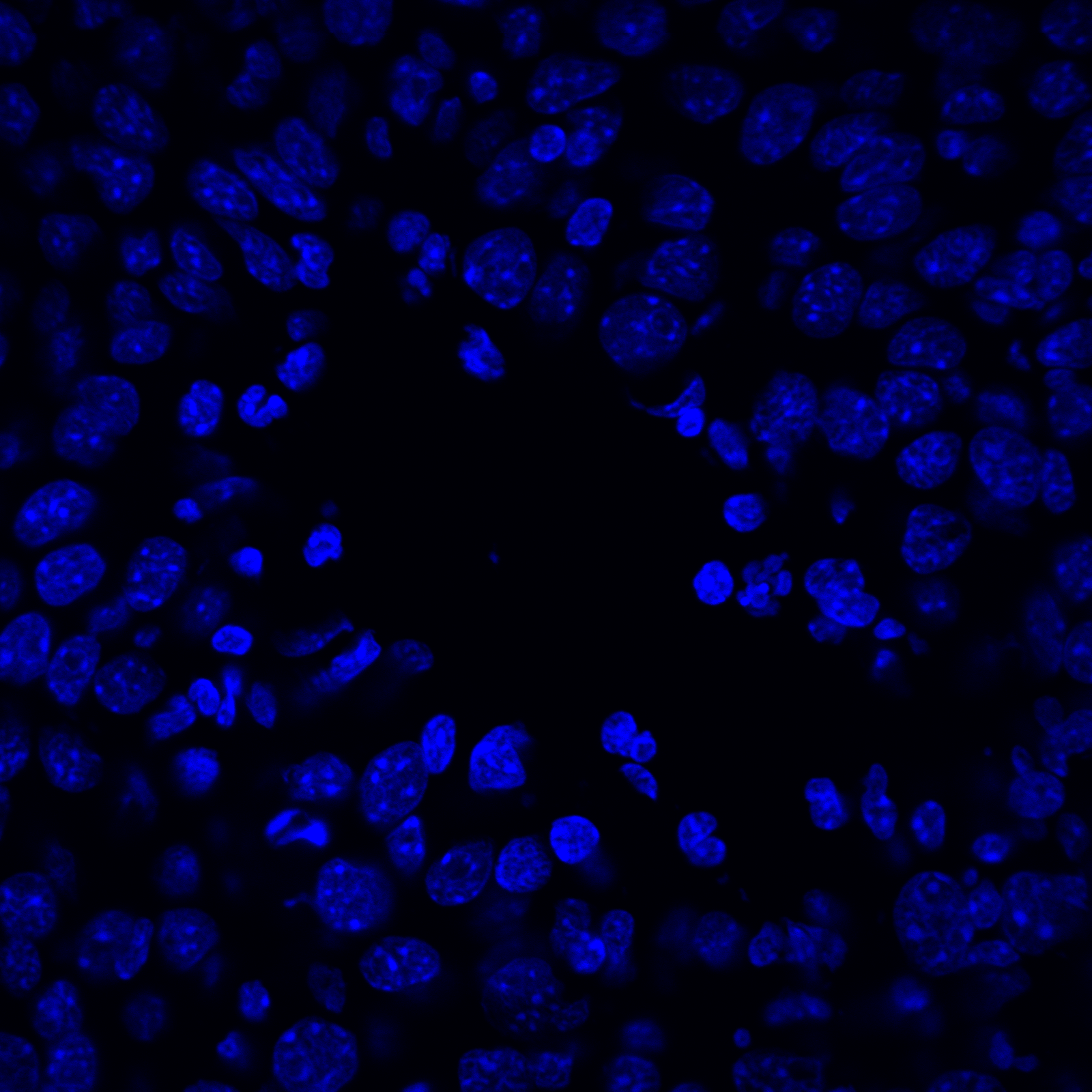

Supplement: Supplementary file 7 — Source Data for Figure 3 [file EMMM-12-e11223-s005.zip › Fig3/Fig.3C/AFR_6u_2_C001.tif]

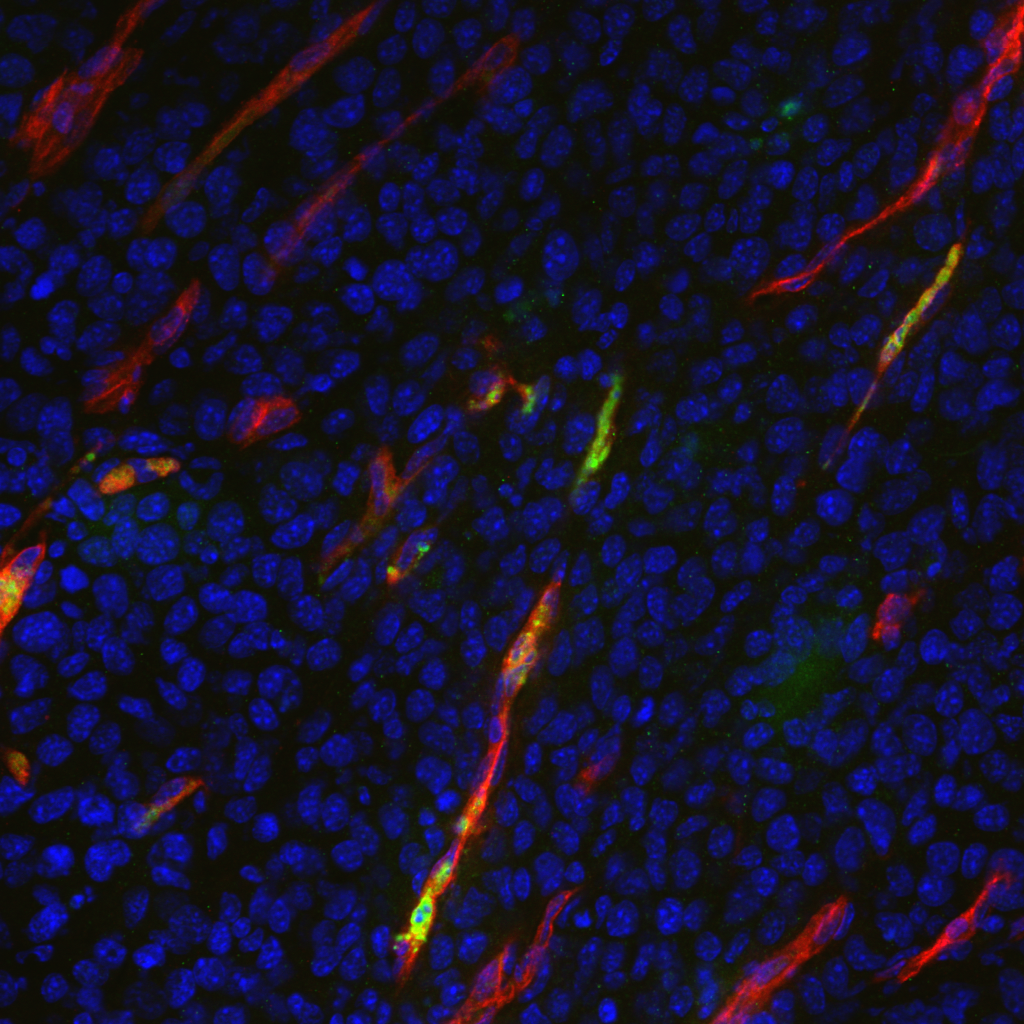

Supplement: Supplementary file 10 — Source Data for Figure 6 [file EMMM-12-e11223-s008.zip › Fig6/Fig.6C/combo_4u_20x_.tif]

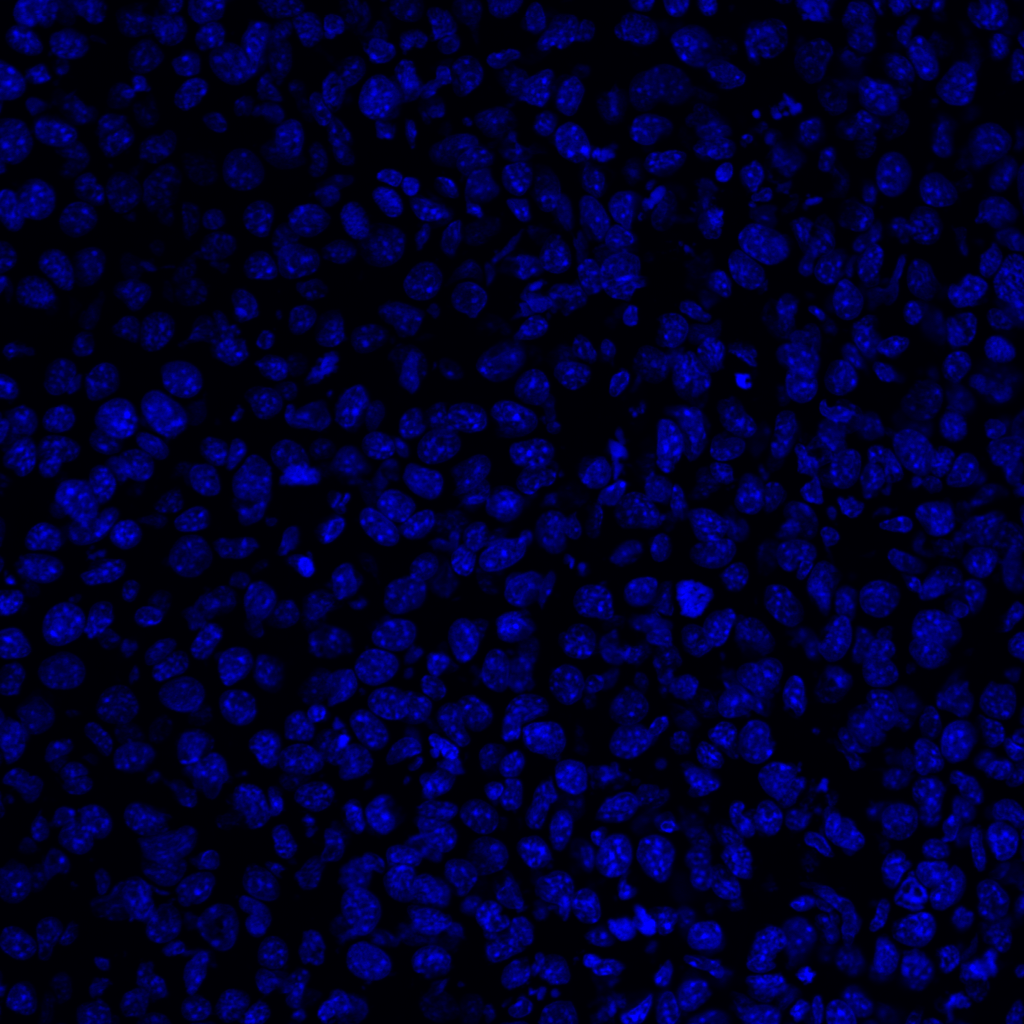

Supplement: Supplementary file 10 — Source Data for Figure 6 [file EMMM-12-e11223-s008.zip › Fig6/Fig.6C/combo_5u_20x_C001.tif]

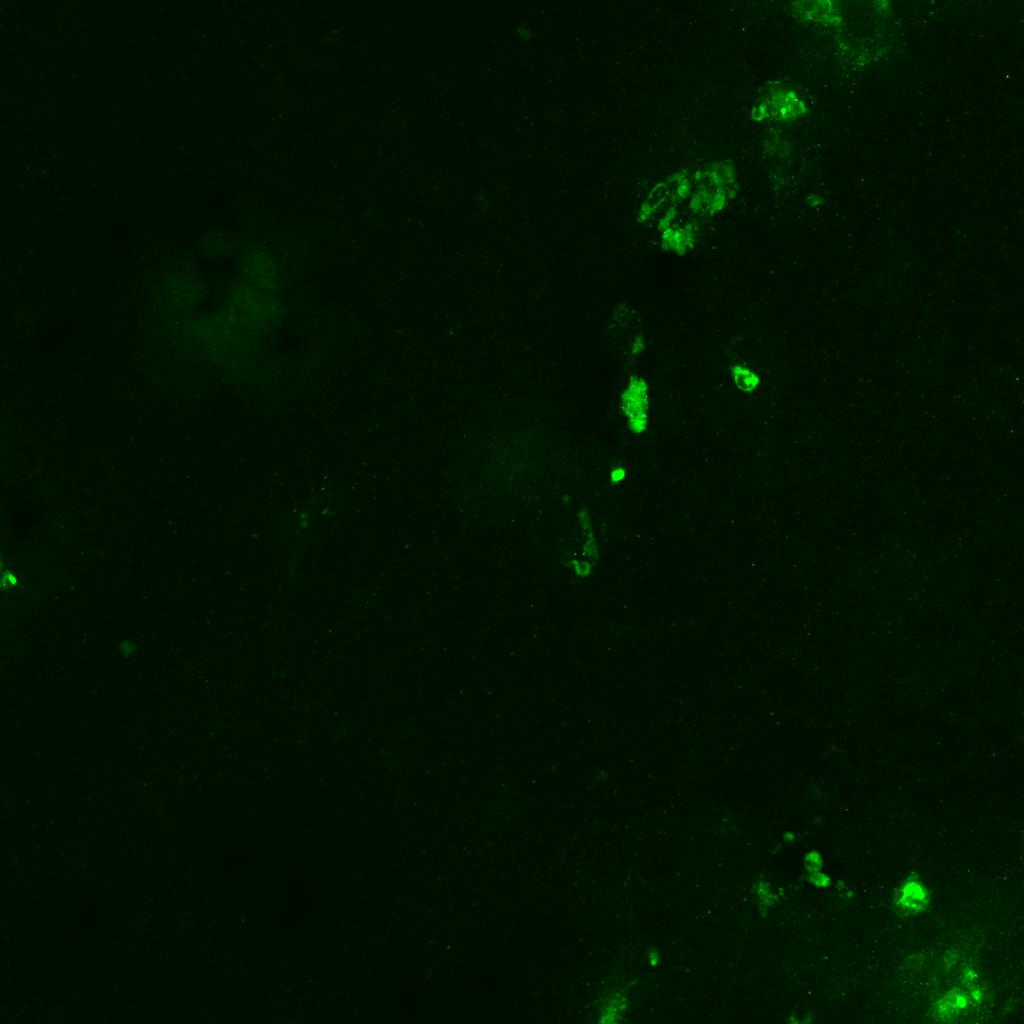

Supplement: Supplementary file 10 — Source Data for Figure 6 [file EMMM-12-e11223-s008.zip › Fig6/Fig.6C/combo_5u_20x_C002.tif]

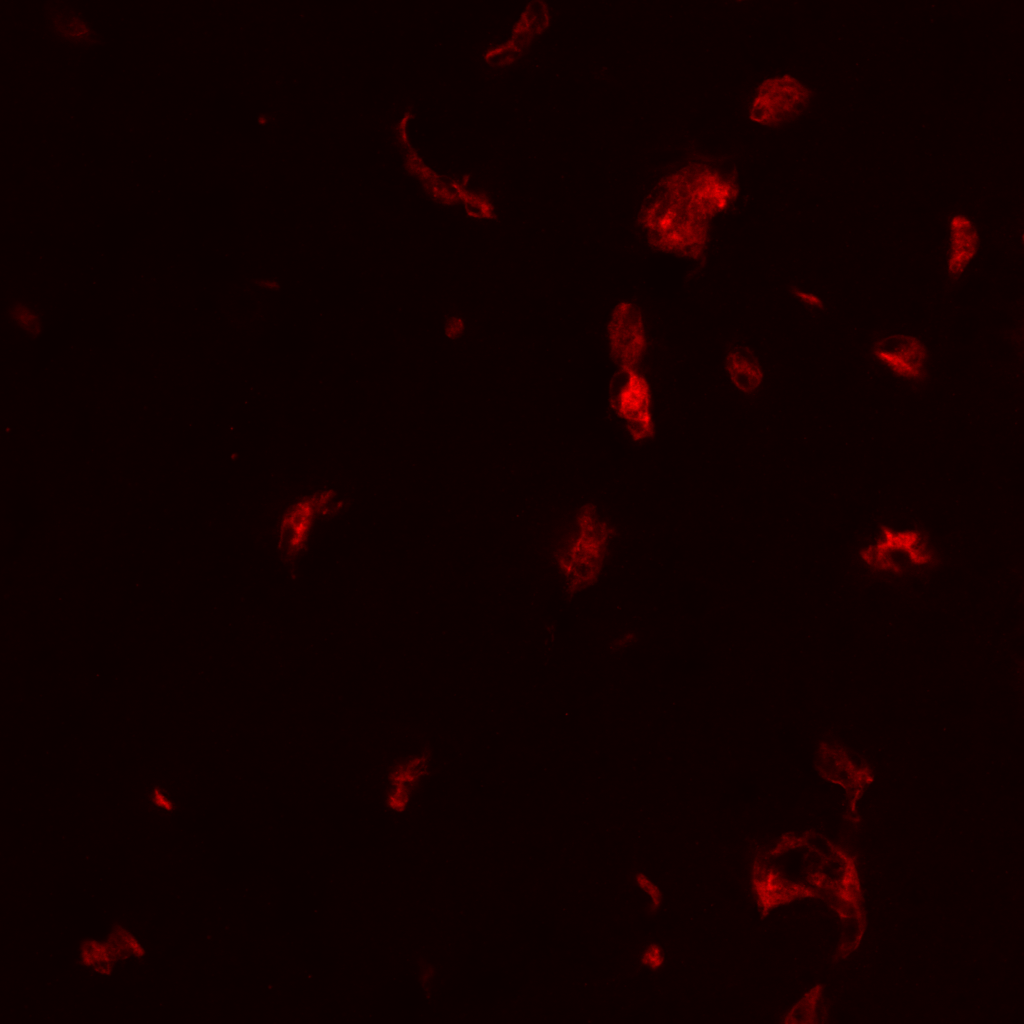

Supplement: Supplementary file 10 — Source Data for Figure 6 [file EMMM-12-e11223-s008.zip › Fig6/Fig.6C/combo_5u_20x_C003.tif]

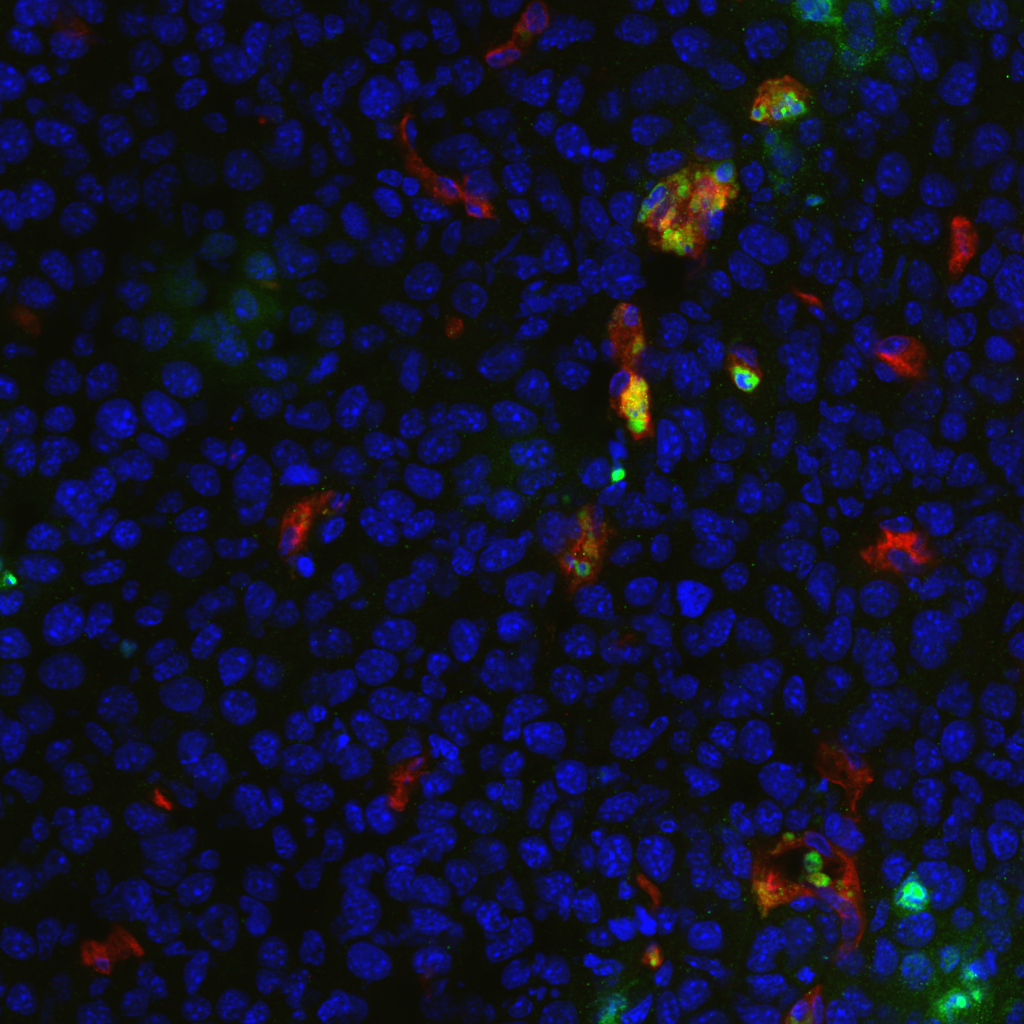

Supplement: Supplementary file 10 — Source Data for Figure 6 [file EMMM-12-e11223-s008.zip › Fig6/Fig.6C/combo_5u_20x_.tif]

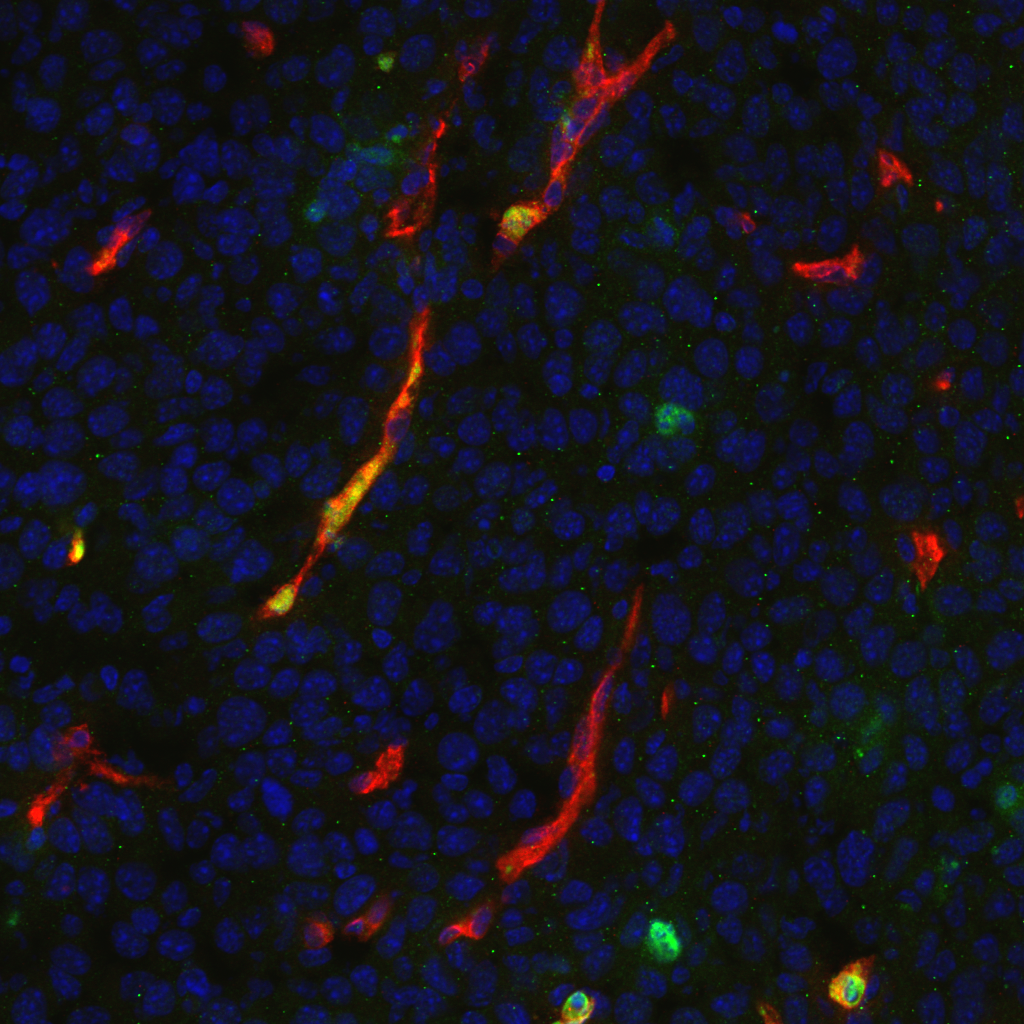

Supplement: Supplementary file 10 — Source Data for Figure 6 [file EMMM-12-e11223-s008.zip › Fig6/Fig.6C/combo_3u_20x_.tif]

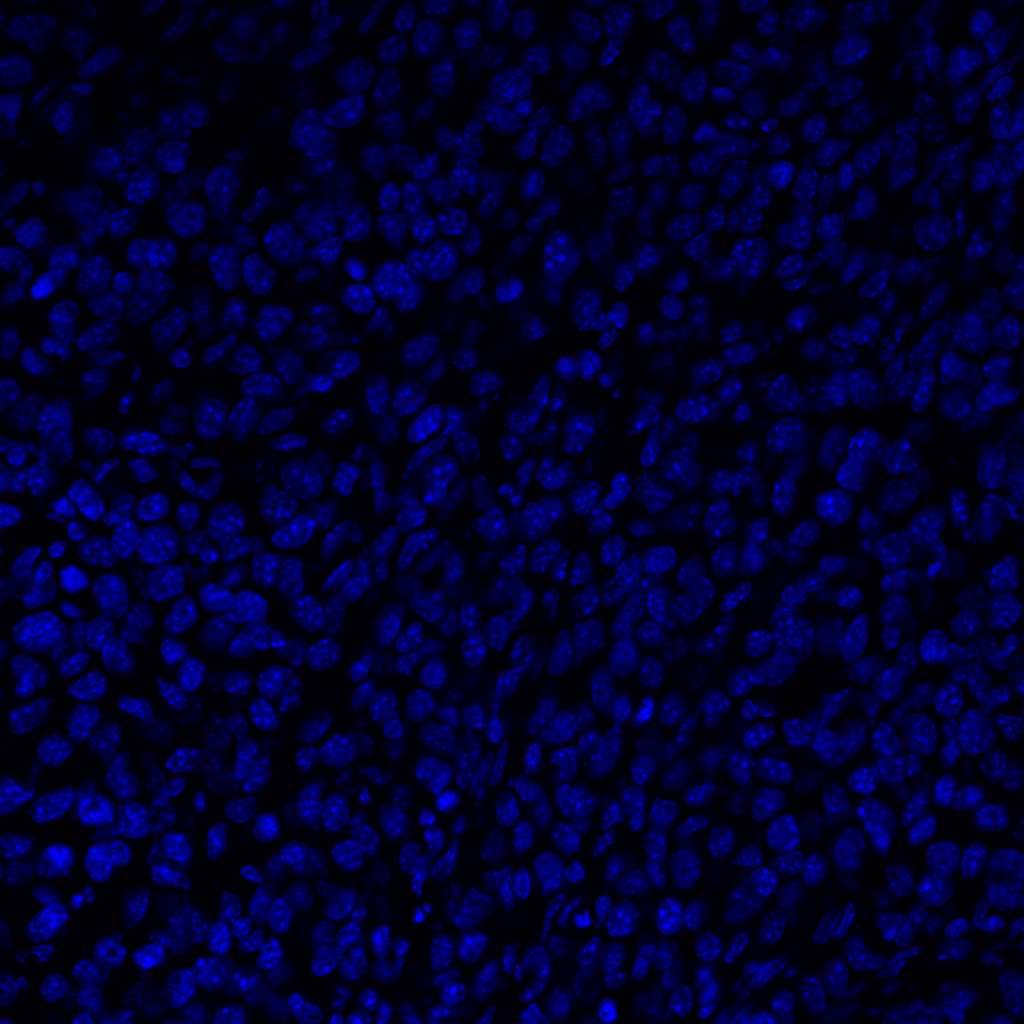

Supplement: Supplementary file 10 — Source Data for Figure 6 [file EMMM-12-e11223-s008.zip › Fig6/Fig.6C/combo_4u_20x_C001.tif]

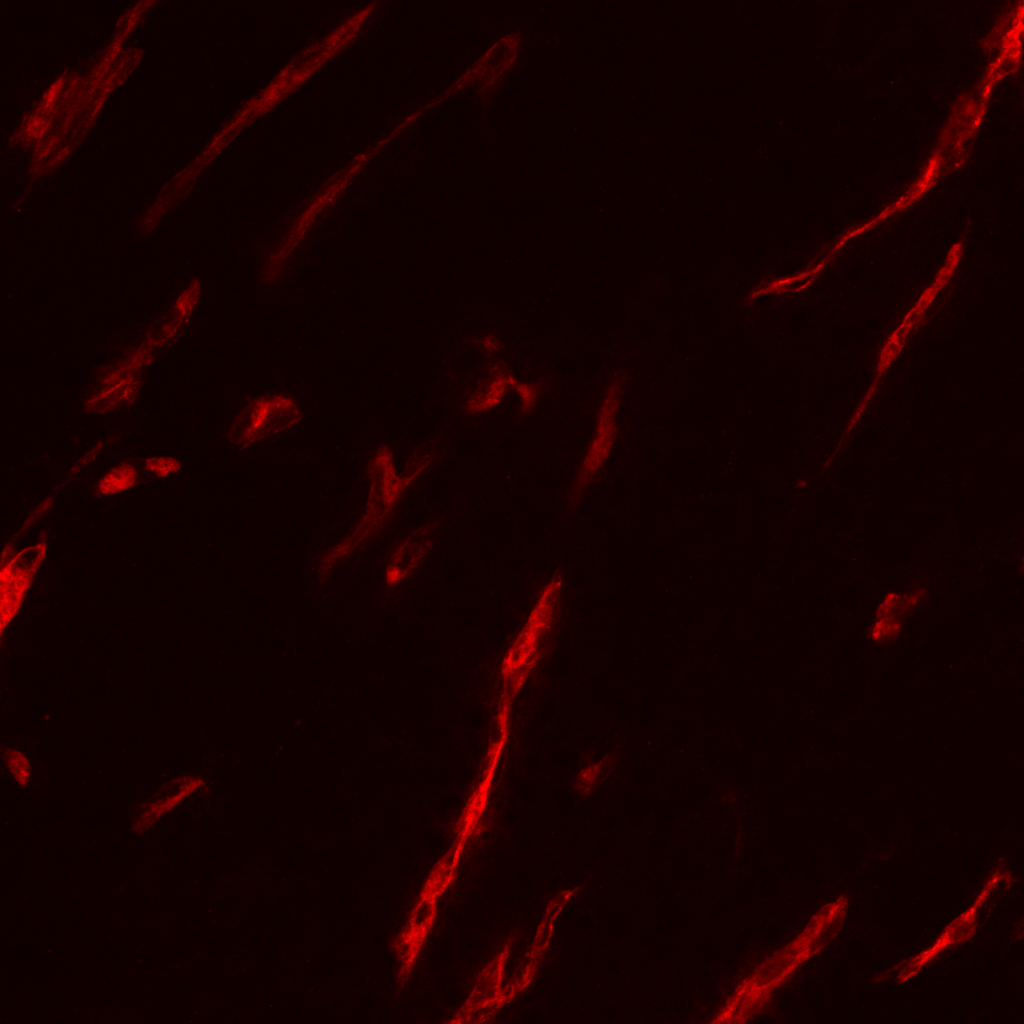

Supplement: Supplementary file 10 — Source Data for Figure 6 [file EMMM-12-e11223-s008.zip › Fig6/Fig.6C/combo_4u_20x_C003.tif]

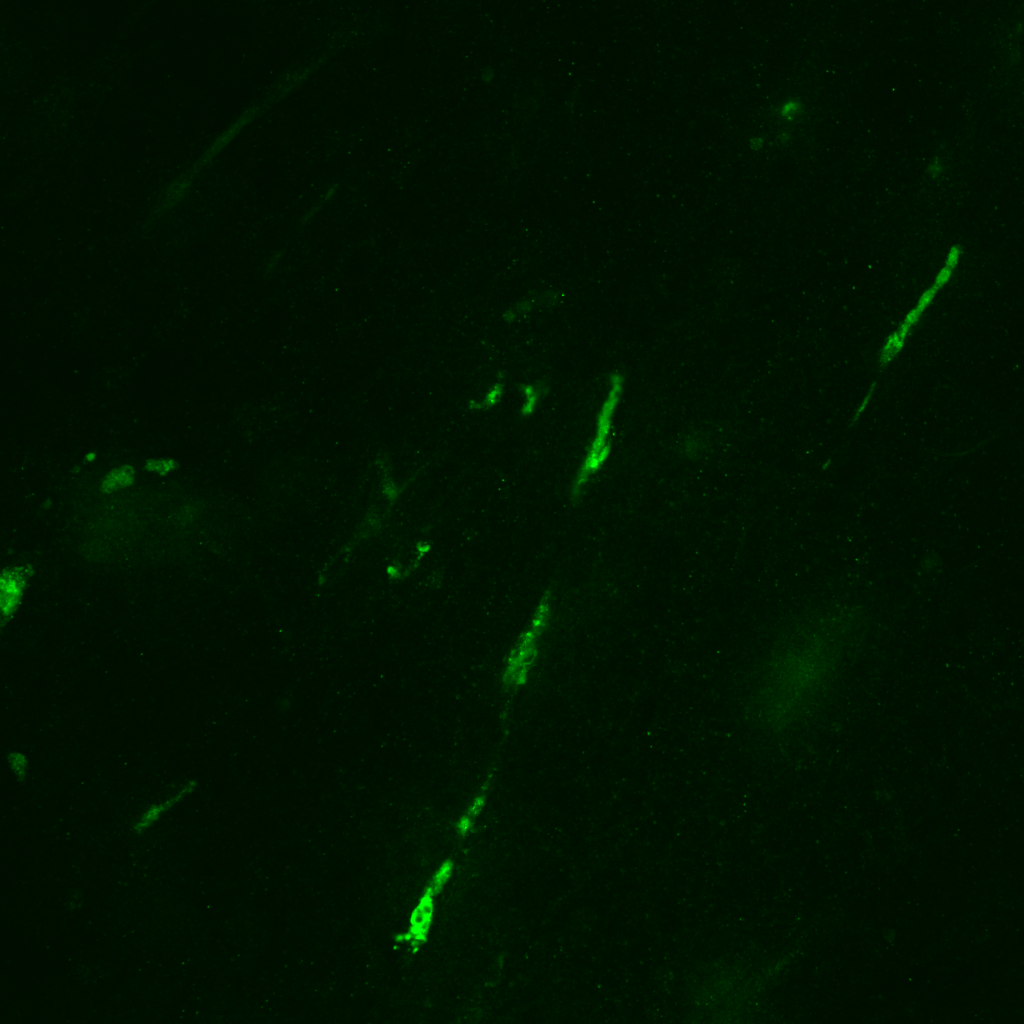

Supplement: Supplementary file 10 — Source Data for Figure 6 [file EMMM-12-e11223-s008.zip › Fig6/Fig.6C/combo_4u_20x_C002.tif]

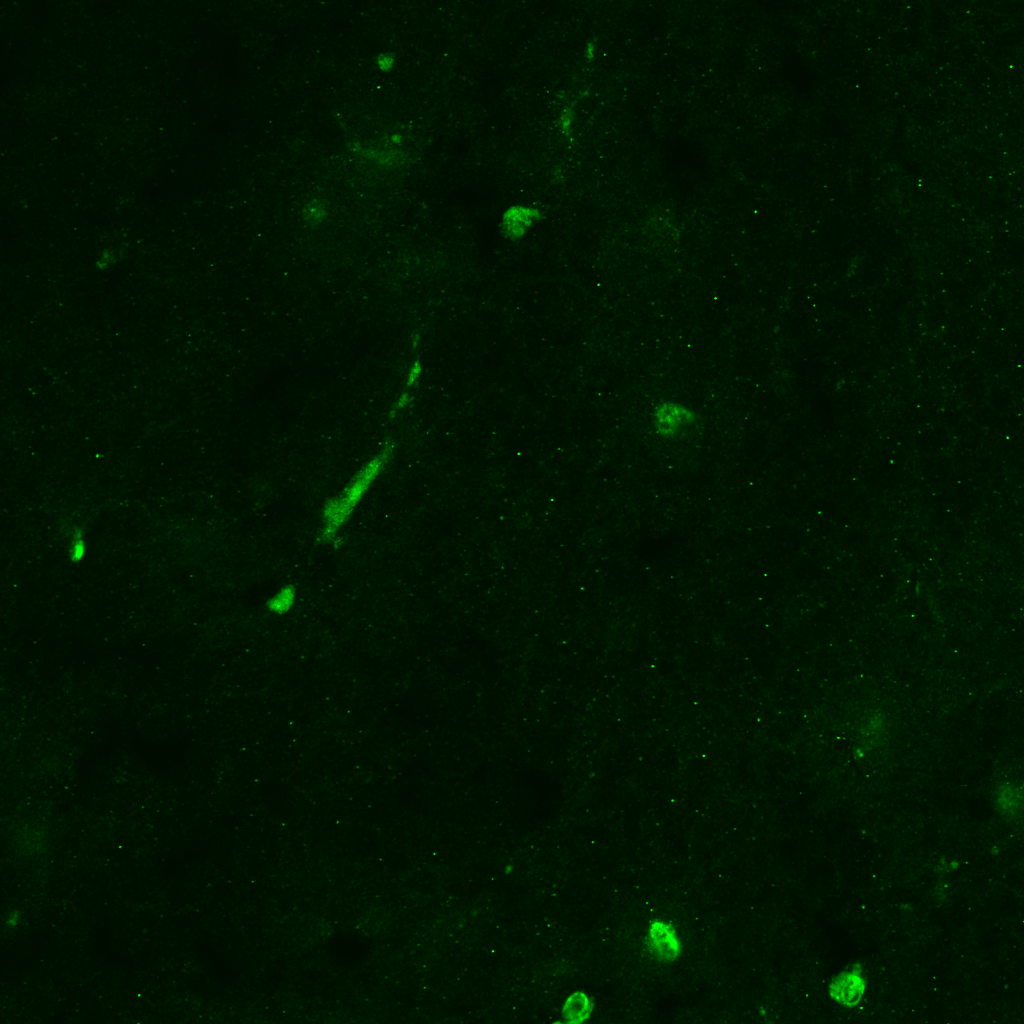

Supplement: Supplementary file 10 — Source Data for Figure 6 [file EMMM-12-e11223-s008.zip › Fig6/Fig.6C/combo_3u_20x_C002.tif]

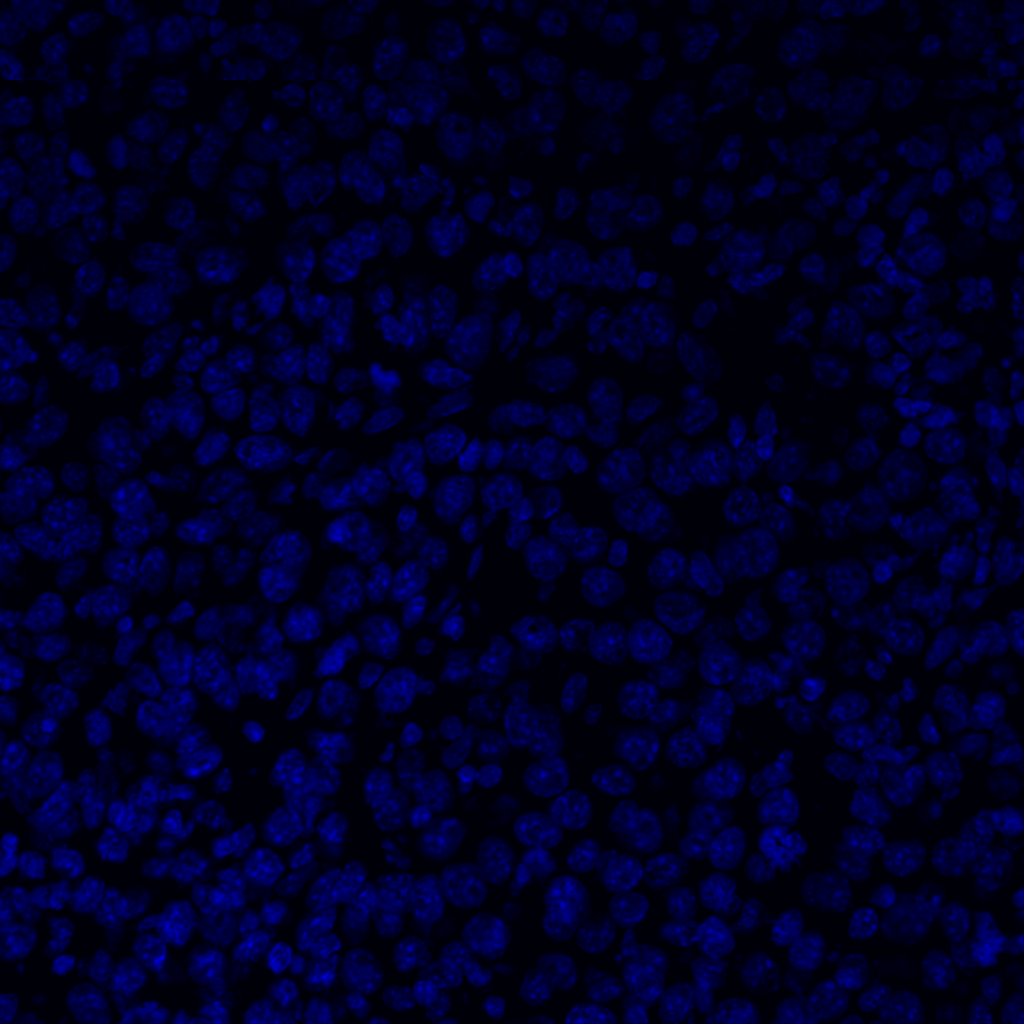

Supplement: Supplementary file 10 — Source Data for Figure 6 [file EMMM-12-e11223-s008.zip › Fig6/Fig.6C/ctrl_20x_C001.tif]

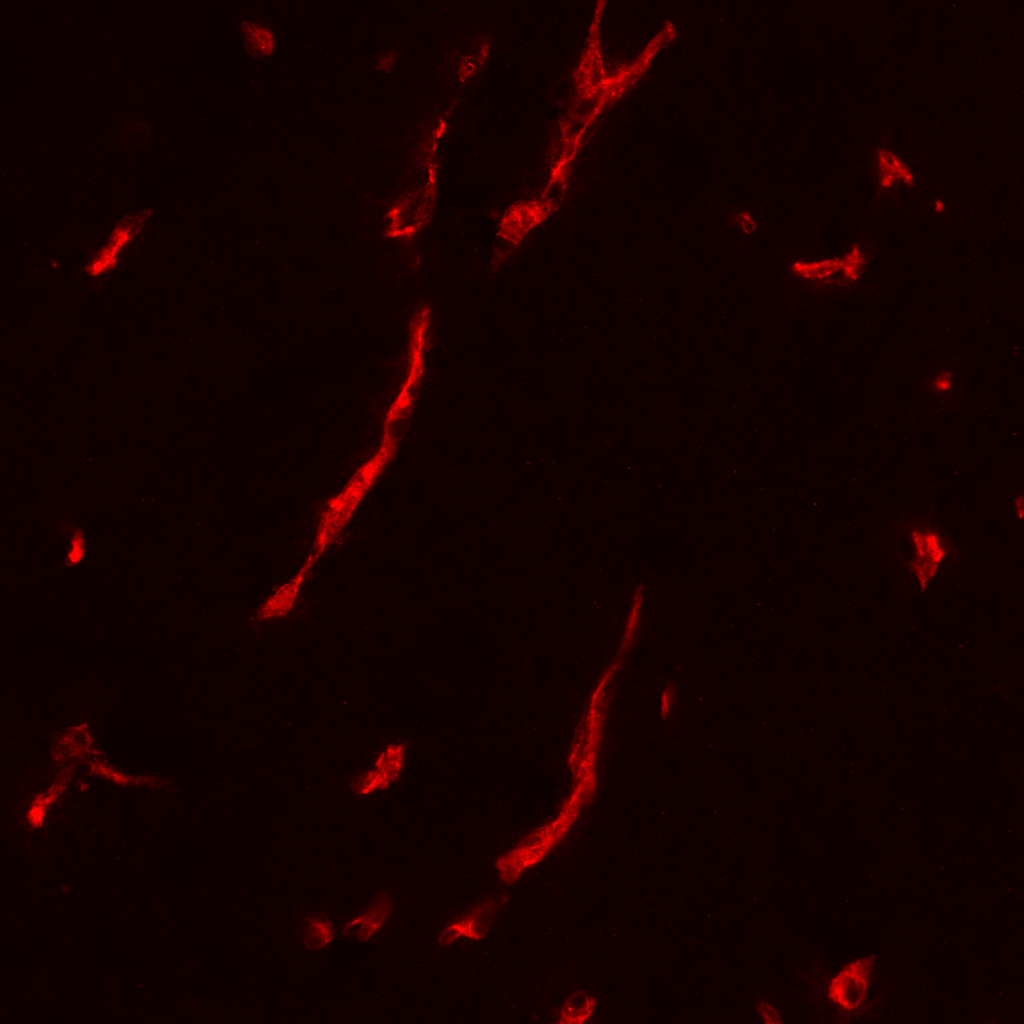

Supplement: Supplementary file 10 — Source Data for Figure 6 [file EMMM-12-e11223-s008.zip › Fig6/Fig.6C/combo_3u_20x_C003.tif]

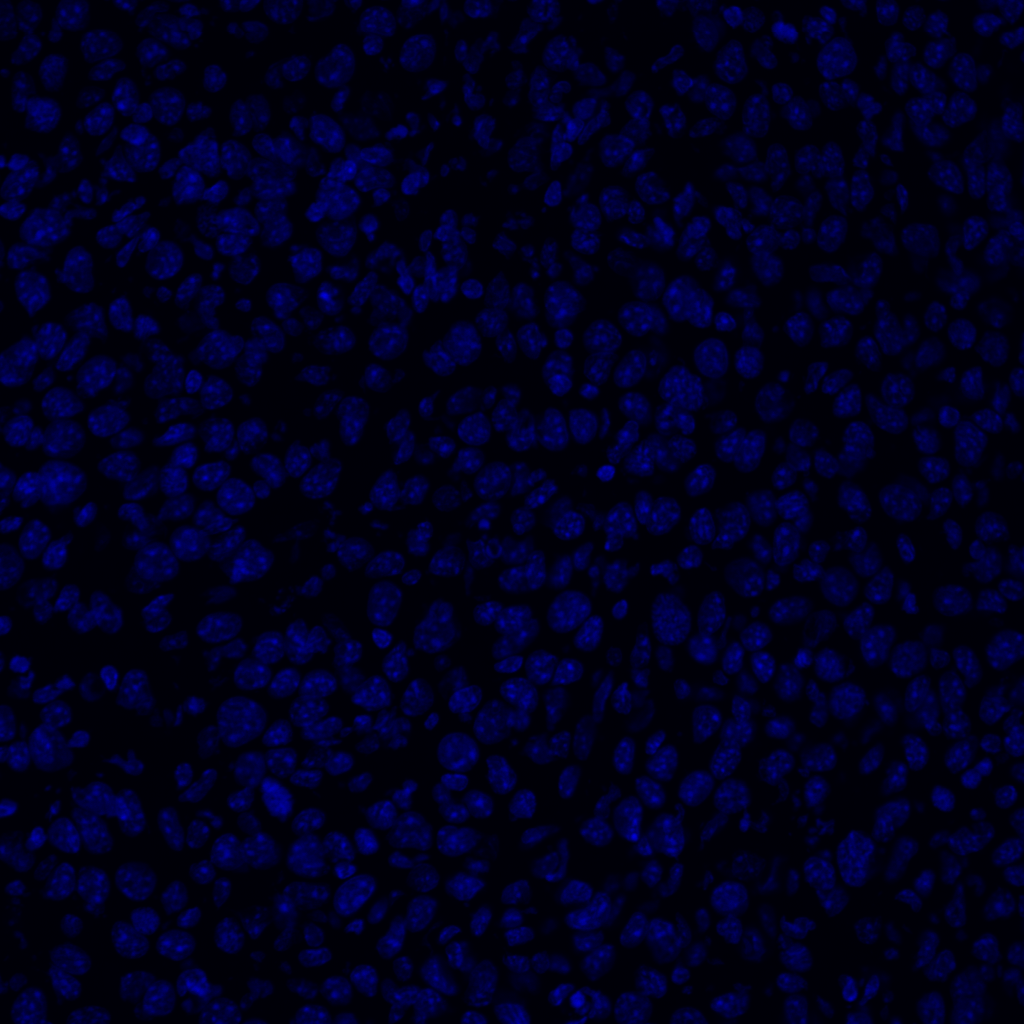

Supplement: Supplementary file 10 — Source Data for Figure 6 [file EMMM-12-e11223-s008.zip › Fig6/Fig.6C/combo_3u_20x_C001.tif]

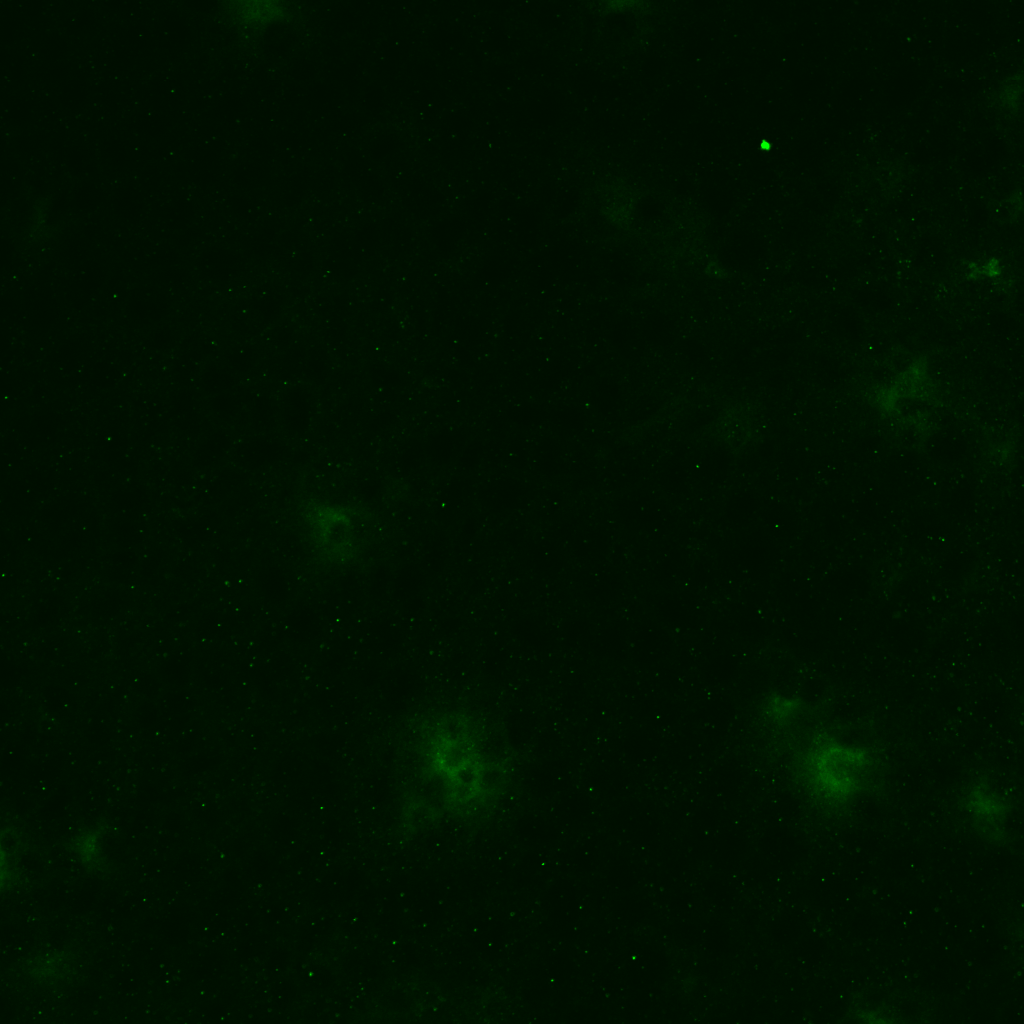

Supplement: Supplementary file 10 — Source Data for Figure 6 [file EMMM-12-e11223-s008.zip › Fig6/Fig.6C/ctrl_20x_C002.tif]

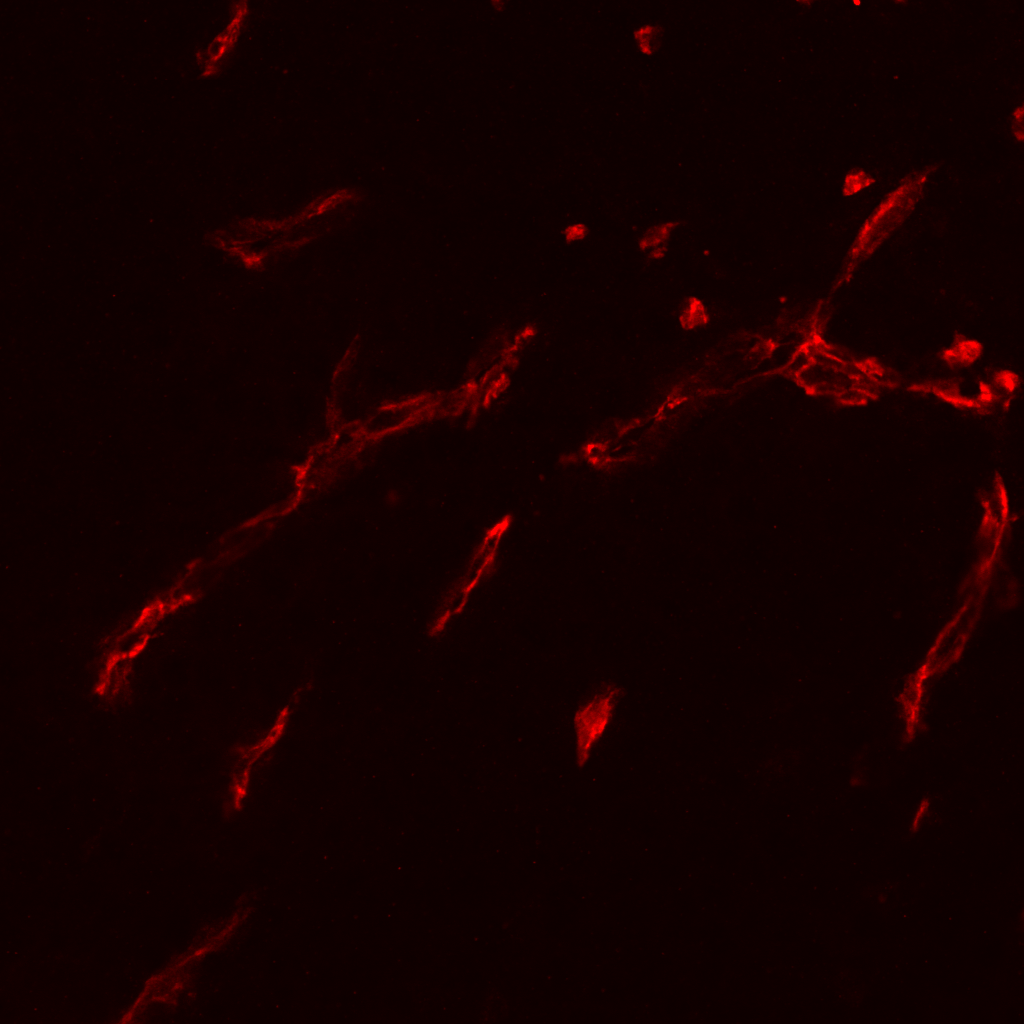

Supplement: Supplementary file 10 — Source Data for Figure 6 [file EMMM-12-e11223-s008.zip › Fig6/Fig.6C/ctrl_20x_C003.tif]

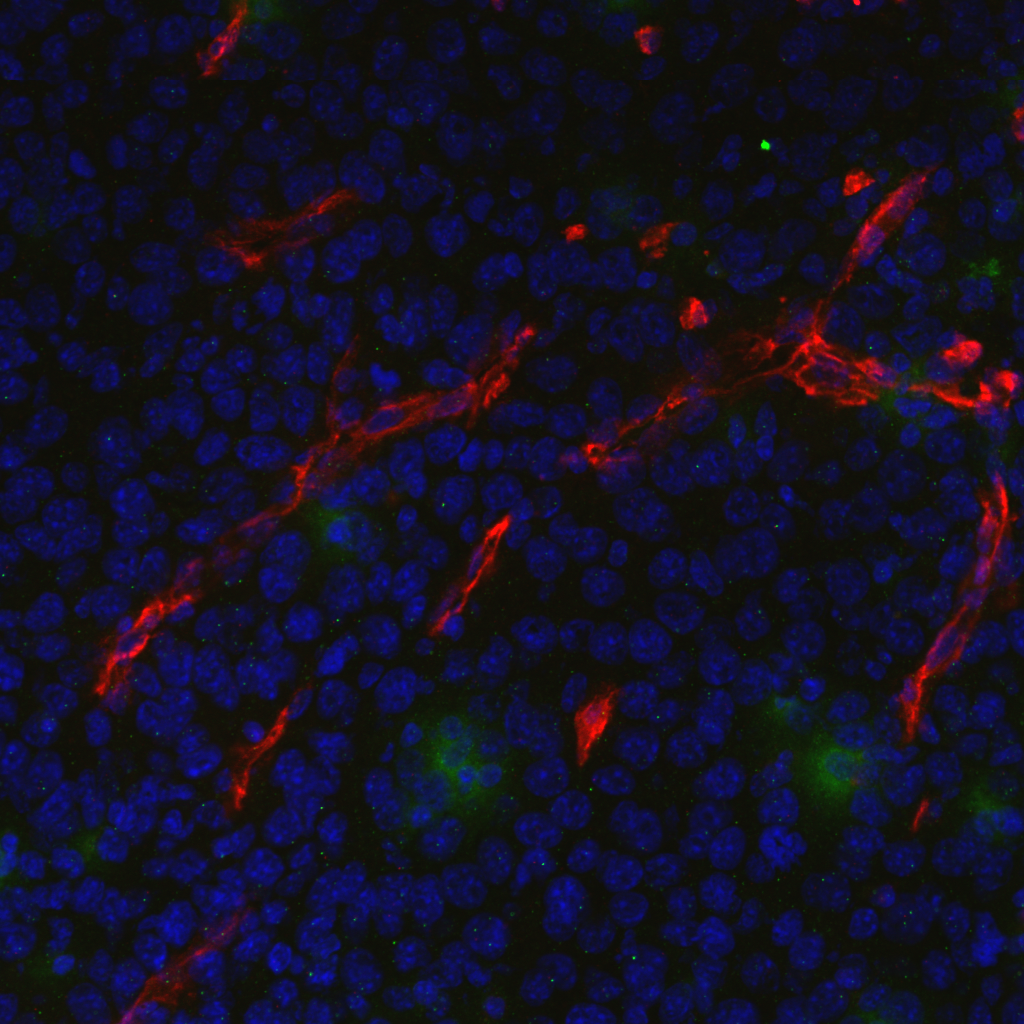

Supplement: Supplementary file 10 — Source Data for Figure 6 [file EMMM-12-e11223-s008.zip › Fig6/Fig.6C/ctrl_20x_.tif]
